# SLITRK2 knockdown rescues axonal and functional neuromuscular junction deficits in human iPSC-derived mutant FUS-ALS motor neurons

**DOI:** 10.1038/s41467-026-78419-7

**Published:** 2026-10-15

**Authors:** Rianne de Jongh, Anna F. Wiersema, Charlotte H. van Dijk, Roos C. Verdegaal, Mareine M. J. Smulders, Onur Basak, Jelle van Os, Sofie Peeters, Laura Olmedo-Martínez, Lauri M. Bloemenkamp, Keith M. Garner, Sandrine Da Cruz, Max Koppers, Alessandro Iuliano, W. W. M. Pim Pijnappel, Kevin P. Kenna, R. Jeroen Pasterkamp

**Affiliations:** 1 Department of Translational Neuroscience, University Medical Center Utrecht Brain Center, Utrecht University, Utrecht, The Netherlands; 2 VIB-KU Leuven Center for Neuroscience, Department of Neurosciences, KU Leuven, Leuven Brain Institute, Leuven, Belgium; 3 Cell Biology, Neurobiology and Biophysics, Department of Biology, Faculty of Science, Utrecht University, Utrecht, The Netherlands; 4 Department of Pediatrics, Department of Clinical Genetics, Center for Lysosomal and Metabolic Diseases, Erasmus MC University Medical Center, Rotterdam, The Netherlands; 5 Present Address: Department of Biochemistry and Biophysics, Stockholm University & Department of Cell and Molecular Biology, Karolinska Institutet, Biomedicum, Stockholm, Sweden; 6 Present Address: Center for Neurogenomics and Cognitive Research, Department of Functional Genomics, Vrije Universiteit Amsterdam, Amsterdam, The Netherlands

**Keywords:** Amyotrophic lateral sclerosis, Molecular neuroscience, Mechanisms of disease, Amyotrophic lateral sclerosis

## Abstract

Amyotrophic lateral sclerosis (ALS) is a fatal neurodegenerative disease characterized by motor neuron (MN) loss caused by early axonal and synaptic changes. However, the mechanisms underlying these changes remain incompletely understood. Here, we exploit FUS-ALS to interrogate cell compartment-specific changes in RNA expression and regulation in human induced pluripotent stem cell (iPSC)-derived MN. This approach identifies compartment-specific 3’ untranslated region (3’UTR) isoform expression in human healthy control MN axons. Further, it unveils altered 3’ UTR isoforms in ALS in different cellular compartments in genes such as *SNCA*, previously linked to neurodegeneration. Expression of SLITRK2 is most strongly and consistently upregulated across multiple FUS-ALS models, and its knockdown normalizes axonal defects linked to FUS-ALS. Using a fully iPSC-derived neuromuscular microfluidics protocol, we show that SLITRK2 knockdown in FUS-ALS MN rescues neuromuscular junction (NMJ) signaling without affecting NMJ innervation or MN hypo-excitability. Our study provides insight into compartment-specific 3’UTR expression in ALS, presents a robust iNMJ-on-a-chip model, and links elevated SLITRK2 to axonal and synaptic defects in FUS-ALS.

## Abstract (web summary)

The mechanisms driving early axonal and synaptic dysfunction in ALS remain unclear. Here, the authors identify compartment-specific RNA changes and identify SLITRK2 as a driver of axonal and synaptic defects in FUS-ALS using an iNMJ-on-a-chip model.

## Introduction

Amyotrophic lateral sclerosis (ALS) is a fatal adult-onset neurodegenerative disorder characterized by degeneration of upper and lower spinal motor neurons (MN), leading to progressive muscle weakness and atrophy. Ultimately, loss of respiratory function results in death of patients typically within 2–5 years after symptom onset^1, 2^. Accumulating evidence suggests that degeneration of neuromuscular junctions (NMJs) and distal axons is an early event in the pathogenesis of ALS^3, 4^. While these early events represent valuable therapeutic targets, the molecular mechanisms driving the selective vulnerability of distal axons and synapses in ALS remain incompletely understood. Maintaining neuronal viability and function in highly polarized cells with long cellular processes, such as MN and their axons, depends on tightly regulated local processes. These include efficient axonal transport and precise spatiotemporal control of RNA metabolism, including mRNA localization, stability, and local translation^5–7^. Post-transcriptional regulation mediated by elements within 3’ untranslated regions (3’ UTRs) is key to this spatial control^8^. For example, alternative polyadenylation (APA) generates mRNA isoforms with different 3’ UTR lengths^9^, enabling differential compartmentalization of transcripts within neurons^10–13^. Changes in these local RNA regulatory mechanisms can severely impact neuron function and survival, but remain largely unknown in the context of human MN and ALS.

There is a strong genetic contribution to ALS aetiology, with mutations in several genes encoding RNA-binding proteins (RBPs) being implicated. Among these, mutations in definitive ALS genes like *TDP-43*, *FUS*, and *ATXN2* are well-established, while a growing body of evidence also links other RBPs, such as *TAF15*, *EWSR1*, *hnRNPA1*, *hnRNPA2/B1*, *MATR3* and *TIA1* to the disease^14–16^. This has established dysregulation of RNA metabolism as a central pathogenic mechanism in ALS. Mutations in *FUS* (fused in sarcoma) are associated with particularly aggressive disease progression and a higher prevalence in juvenile-onset ALS (FUS-ALS)^17–19^. *FUS* encodes an RBP that predominantly resides in the nucleus, where it mediates key roles in DNA repair and RNA metabolism, including in transcription, pre-mRNA splicing and APA. FUS often mediates these functions by directly binding 3’ UTR sequences to regulate mRNA stability, degradation, and subcellular localization^20, 21^. In addition to nuclear functions, FUS dynamically shuttles between the nucleus and cytoplasm, contributing to the regulation of mRNA transport and local translation^22, 23^. This nucleocytoplasmic balance is disrupted by many of the reported ALS-associated *FUS* mutations, particularly those affecting the C-terminal nuclear localization signal (e.g., P525L). These mutations lead to cytoplasmic mislocalization of FUS and the formation of cytosolic inclusions^24^. Such mislocalization impairs the nuclear functions of FUS, including those related to splicing and APA regulation, while simultaneously causing toxic gain-of-function effects in the cytoplasm^19, 25–28^.

At the cellular level, *FUS* mutations cause structural and functional abnormalities in axons and at the NMJ in various in vitro and in vivo models, often preceding MN degeneration^29–36^. FUS regulates the stability, transport and translation of several mRNAs in the axon and NMJ, and *FUS* mutations can affect mRNA expression levels in axons^37–39^. *FUS* mutations also influence post-transcriptional modification and 3’-end processing factors, such as splicing, mRNA stability and 3’ UTR length^21, 40–44^. It is therefore plausible that ALS-associated mutations in *FUS* and other RBPs cause changes in the expression and distribution of specific (m)RNA isoforms in specific subcellular compartments, such as the axon. However, while previous studies have explored the axonal transcriptome of MN using various RNA-sequencing (RNA-seq) approaches^10, 39, 45–50^, our understanding of the distribution of specific RNA isoforms in MN axons, e.g., as a consequence of APA, is rather rudimentary. In addition, whether ALS mutations affect compartment-specific isoform expression or distribution is not known.

To further study changes in local gene expression in specific subcellular compartments of the human neuromuscular system and identify therapeutic targets, we exploited FUS-ALS human induced pluripotent stem cell (iPSC)-derived MN and developed a human iPSC-derived (i)NMJ-on-a-chip platform. To date, most humanized NMJ-on-a-chip models utilize primary myoblasts, immortalized muscle cell lines or muscle derived from alternative stem cell sources (e.g., mesangioblasts)^36, 51–55^ due to the persistent challenge to reliable generate mature, functional myotubes from iPSCs. To fill this void, we adapted a robust, transgene-free protocol to efficiently differentiate iPSC-derived myogenic progenitors into mature, spontaneously contracting myotubes^56–58^. This enabled the establishment of a fully iPSC-based iNMJ-on-a-chip model and one of the first studies in which such a model was used to assess functional ALS phenotypes.

In this study, axonal RNA was isolated from different FUS-ALS models and subjected to CEL-seq2^59^. This allowed simultaneous analysis of axonal mRNA expression changes and 3’ UTR isoform alterations. It identified compartment-specific 3’UTR isoform expression in healthy control MN axons and unveiled altered 3’ UTR isoforms in ALS in different cellular compartments. Expression of SLITRK2 was most strongly and consistently upregulated across multiple FUS-ALS models, and its knockdown normalized axonal defects linked to FUS-ALS. While previous work had shown changes in NMJ number and the ability of selective inhibitors to reverse these defects^36^, our results extend these observations by showing that targeting a single gene can also rescue functional deficits at the human NMJ. Interestingly, knockdown of SLITRK2, a protein implicated in neurite and synapse biology^60–65^, in FUS-ALS MN rescued NMJ signaling without affecting NMJ innervation or MN hypo-excitability. Together, our study provides insight into compartment-specific gene and 3’UTR expression changes in ALS, presents a fully iPSC-derived iNMJ-on-a-chip model, and links elevated SLITRK2 to axonal and functional synaptic defects in FUS-ALS.

## Results

### Isolation of axonal RNA from iPSC-derived motor neurons in microfluidic devices

**Fig. 1:**
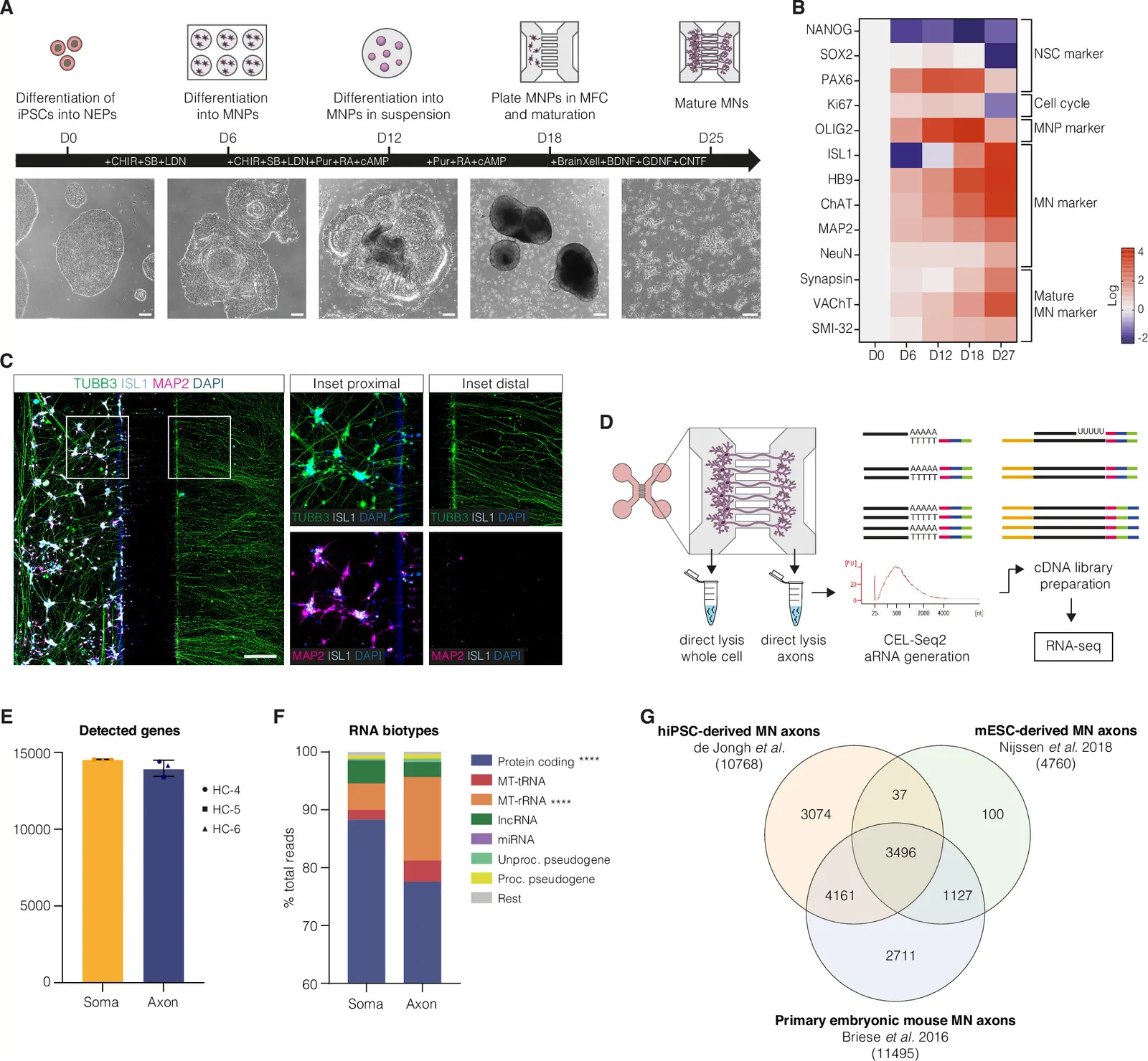
Generation and characterization of iPSC-derived motor neurons for axonal transcriptomic analysis. **A** Schematic showing the differentiation protocol of iPSCs into spinal MN, including subsequent culture within microfluidic devices. Representative brightfield pictures illustrate morphology at the indicated time points. Scale bar D0-D18: 200 μm, D25: 100 μm. NEP neuroepithelial progenitor, MNP motor neuron progenitor, MFC microfluidic chip.  **B** mRNA expression levels of several developmental and mature (neuronal) markers at different time points during differentiation (*n* = 1). Expression levels are normalized to the housekeeping genes *GAPDH* and *β-actin*. NSC neural stem cell, MNP motor neuron progenitor. **C** Expression of MN somatodendritic markers ISL1 and MAP2, and neurite marker TUBB3 in iPSC-derived MN cultured in microfluidic devices at D27. Scale bar: 200 μm. Insets: magnification of somatodendritic (proximal) and axonal (distal) compartments showing ISL1, MAP2 and TUBB3 expression. **D** Schematic illustrating the workflow for RNA isolation and sequencing from somatodendritic (whole cell) and axonal compartments of iPSC-derived MN. RNA was directly lysed from each compartment, followed by aRNA generation and cDNA preparation according to the CEL-seq2 protocol prior to RNA-seq. **E** Number of detected genes in somatodendritic and axonal samples. Data is presented as mean ± SD (*n* = 3 biological replicates, *t* = 1 technical replicate). Paired two-tailed T-test (*p* = 0.1903). **F** RNA biotype classification in somatodendritic and axonal samples. Data is presented as mean (*n* = 3 biological replicates, *t* = 1 technical replicate). Two-way ANOVA. **** *p* < 0.0001. **G** Venn diagram comparing the overlap between genes identified in our human iPSC-derived MN axons compared to those found in previously published datasets from mouse embryonic stem cell-derived MN axons^38^ and primary embryonic mouse MN axons^45^. Source data are provided as a Source Data file.

To investigate transcriptional changes in MN axons, we generated iPSC-derived spinal MN cultures in microfluidic devices using an established differentiation protocol^66^, which was modified to enhance maturation and survival (Fig. 1A). MN progenitors (MNPs) were plated in microfluidic devices at day 18 (D18) and robust expression of *HB9*, *ISL1*, *ChAT*, *MAP2*, and *NeuN* was observed by D27, with a concomitant decrease in *SOX2*, *PAX6*, *OLIG2*, and *Ki67*, indicating a postmitotic, mature state (Fig. 1B). Furthermore, expression of the mature MN markers *Synapsin*, *VAChT*, and *SMI-32* increased over time (Fig. 1B). The neuronal marker β-tubulin III (TUBB3) co-localized with the MN transcription factor ISL1 in the proximal compartment of the device and clearly labeled axons crossing into the distal compartment (Fig. 1C). MAP2, a dendritic marker^67^, was only detected in the proximal compartment. This confirmed the selective extension of MN axons, and not dendrites, into the distal compartment.

To characterize the model at the gene expression level, RNA was isolated from the somatodendritic (proximal) and axonal (distal) compartments of healthy control (HC) iPSC-derived MN cultures at D27. The somatodendritic fraction represents a whole-cell lysate as it contains material from cell bodies and axons. It is here referred to as the ‘somatodendritic’ compartment to distinguish it from the axonal compartment. MN were derived from three independent donors, and five devices were pooled per donor to minimize inter-device variability. RNA-seq was performed using a modified version of previously published methods^38^ and the CEL-seq2 protocol^59^ (Fig. 1D). The total number of mapped reads in axonal samples (1.77 × 10^6^ ± 0.27) was significantly lower as compared to somatodendritic samples (3.73 × 10^6^ ± 0.17)(Supplementary Fig. S1A). Nevertheless, a high and similar number of mapped genes were detected in somatodendritic (mean 14509 ± SD 17.35) and axonal samples (13896 ± 561.0)(Fig. 1E). In line with previous research^38, 46^, the axonal transcriptome contained significantly less protein coding RNA (*p* < 0.0001) and a higher proportion of mitochondrial ribosomal RNA (MT-rRNA) (*p* < 0.0001)(Fig. 1F). To assess the validity of our axon-seq data, we compared our dataset to two previously published axonal datasets derived from mouse embryonic stem cell (mESC)-derived MN^38^ and primary embryonic mouse MN^45^ (Fig. 1G and Supplementary Data 1). (4760 genes), we identified 3533 common genes and thus successfully captured 74.2% (3533/4760) of the genes reported in that reference set. An even more substantial overlap was observed with the larger primary embryonic mouse MN dataset (11495 genes), where our analysis identified 7657 common genes, accounting for 66.6% (7657/11495) of their reported axonal transcriptome. These results support the validity of our approach and suggest strongly conserved features of the axonal transcriptome across species.

### Compartment-specific gene expression and 3’ UTR isoforms in iPSC-derived motor neurons

**Fig. 2:**
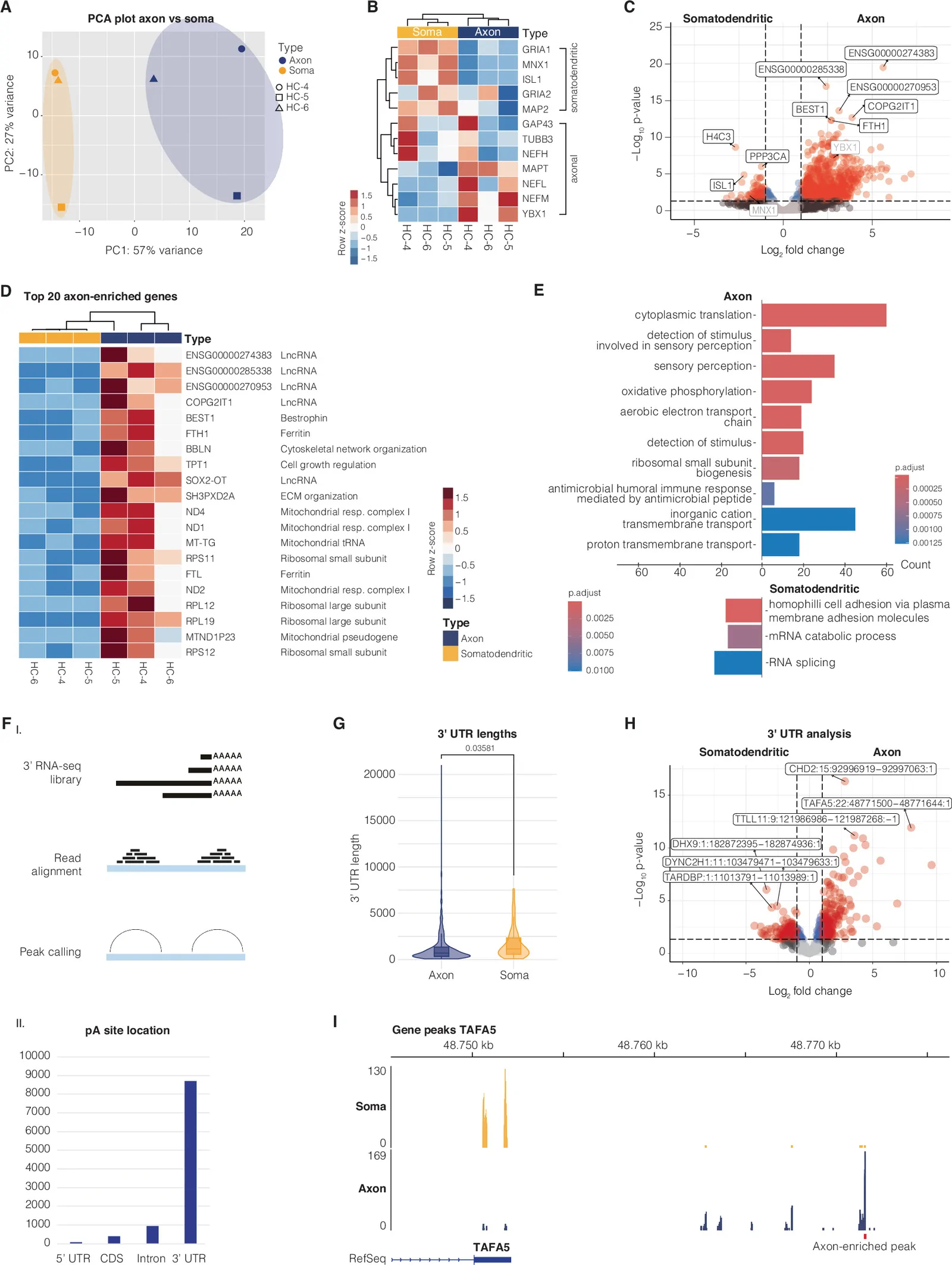
Compartment-specific gene expression and 3’ UTR isoforms in iPSC-derived motor neurons. **A** PCA displaying the transcriptomic separation of somatodendritic (orange) and axonal (blue) samples. **B** Heatmap showing normalized expression levels of established somatodendritic markers (*GRIA1*, *MNX1*, *ISL1*, *GRIA2*, *MAP2* and *GAP43*) and axonal markers (*TUBB3*, *NEFH*, *MAPT*, *NEFL*, *NEFM* and *YBX1*) across individual samples. **C** Volcano plot showing axonal-enriched (right) and somatodendritic-enriched (left) DEGs analyzed by DESeq2 (two-sided Wald test with Benjamini–Hochberg FDR correction; *p*adj < 0.05, |Log2FC | > 1). The top up- and downregulated genes are labeled, and the somatodendritic marker *MNX1* (Hb9) and axonal marker *YBX1* are highlighted in gray. **D** Heatmap displaying normalized expression levels of the top 20 most significantly enriched DEGs in the axonal compartment across all samples. Each gene is annotated with its corresponding functional class. **E** GO analysis of significantly enriched DEGs in the axonal (top) and somatodrendritic (bottom) compartments; performed using ClusterProfiler (v4.10.0) with a two-sided hypergeometric test and Benjamini–Hochberg FDR correction. *p*adj < 0.05. **F** (i) Schematic representation of the method used to identify polyadenylation sites via peak calling, enabled by 3′ RNA-seq library preparation. (ii) Polyadenylation site location of gene peaks identified by APA analysis, categorized by their genomic location (3’ UTR, CDS, intron, 5’ UTR). CDS = Coding sequence. **G** Violin plot illustrating the average 3’ UTR lengths for transcripts enriched in axonal and somatodendritic samples. The line within the violin plot represents the median, the box bounds represent the 25th to 75th percentiles, and the outer shape represents data density (*n* = 3). Paired two-tailed T-test. **H** Volcano plot showing axonal-enriched (right) and somatodendritic-enriched (left) 3’ UTR isoforms analyzed by DESeq2 (two-sided Wald test with Benjamini–Hochberg FDR correction; *p* < 0.05, |Log2FC | > 1). Genes with only one isoform or where all detected isoforms showed significant changes in the same direction were excluded. The top up- and downregulated genes are labeled. **I** IGV visualization of *TAFA5* gene peaks mapped to the RefSeq-Select annotation in axonal and somatodendritic compartments.

Principle component analysis (PCA) based on all expressed genes demonstrated a clear distinction between the two compartments (Fig. 2A). The top 10 genes contributing to PC1, and thus determining axonal identity, included several mitochondrial respiratory chain components, consistent with previous findings^38^, and genes involved in microtubule network regulation (Supplementary Fig. S1B). In addition, analysis of several established somatodendritic and axonal markers demonstrated differential expression between compartments (Fig. 2B). To further study compartment-enriched gene expression, differential gene expression (DEG) analysis was performed. This revealed 739 significantly enriched genes in axons and 139 genes in the somatodendritic compartment after FDR adjustment (Fig. 2C and Supplementary Data 1). The relative low number of somatodendritic-enriched genes might be attributed to the presence of axons in both compartments^48, 49^. The top 20 most abundant axon-enriched genes included long non-coding RNAs (lncRNAs), mitochondrial genes, and components of ribosomal subunits (Fig. 2D). Gene ontology (GO) analysis of axon-enriched genes highlighted pathways related to *cytoplasmic translation*, *sensory perception*, and *oxidative phosphorylation* (Fig. 2E). These findings align with previous studies of the axonal transcriptome^38, 45, 46, 48, 49^ and further validate the axon-seq approach.

Thus far, MN axon-seq studies had focused on differential RNA expression. However, CEL-seq2 also allows analysis of compartment-specific regulation of 3’ UTR isoforms via APA. To identify different 3’ UTR isoforms, peak calling for APA sites using the Sierra R package^68^ was performed (Fig. 2F). Initial validation confirmed that the majority of sequencing peaks mapped within annotated 3’ UTR regions (Fig. 2F) and 9829 sequencing peaks originating from 7406 genes were annotated. Consistent with observations in neuronal cells^12, 13^, 3’ UTR isoforms enriched in MN axons were, on average, shorter as compared to those enriched in the somatodendritic compartment (Fig. 2G). Next, we focused on genes exhibiting multiple distinct gene peaks to specifically identify compartment-specific isoforms. 4179 peaks originating from 1756 genes adhered to this criterion, and differential expression analysis identified compartment-specific enrichment. Following exclusion of genes for which all isoforms showed significant changes in the same direction, which likely reflects overall gene expression changes, 214 isoforms were found to be enriched in axons and 138 isoforms in the somatodendritic compartment (Fig. 2H and Supplementary Data 1). For example, *TAFA5* was among the top axon-enriched hits, and gene peaks with proximal polyadenylation sites in *TAFA5* were abundant in somatodendritic samples (Fig. 2I). *TAFA5* peaks in axonal samples mapped further downstream, presumably representing isoforms with extended 3’ UTRs, as observed in mouse tissue^69^. While further validation of the detected isoforms is needed, these data highlight compartment-specific isoform switching in human iPSC-derived MN axons.

### FUS-ALS mutations induce upregulation of *SLITRK2* in motor neuron axons

**Fig. 3:**
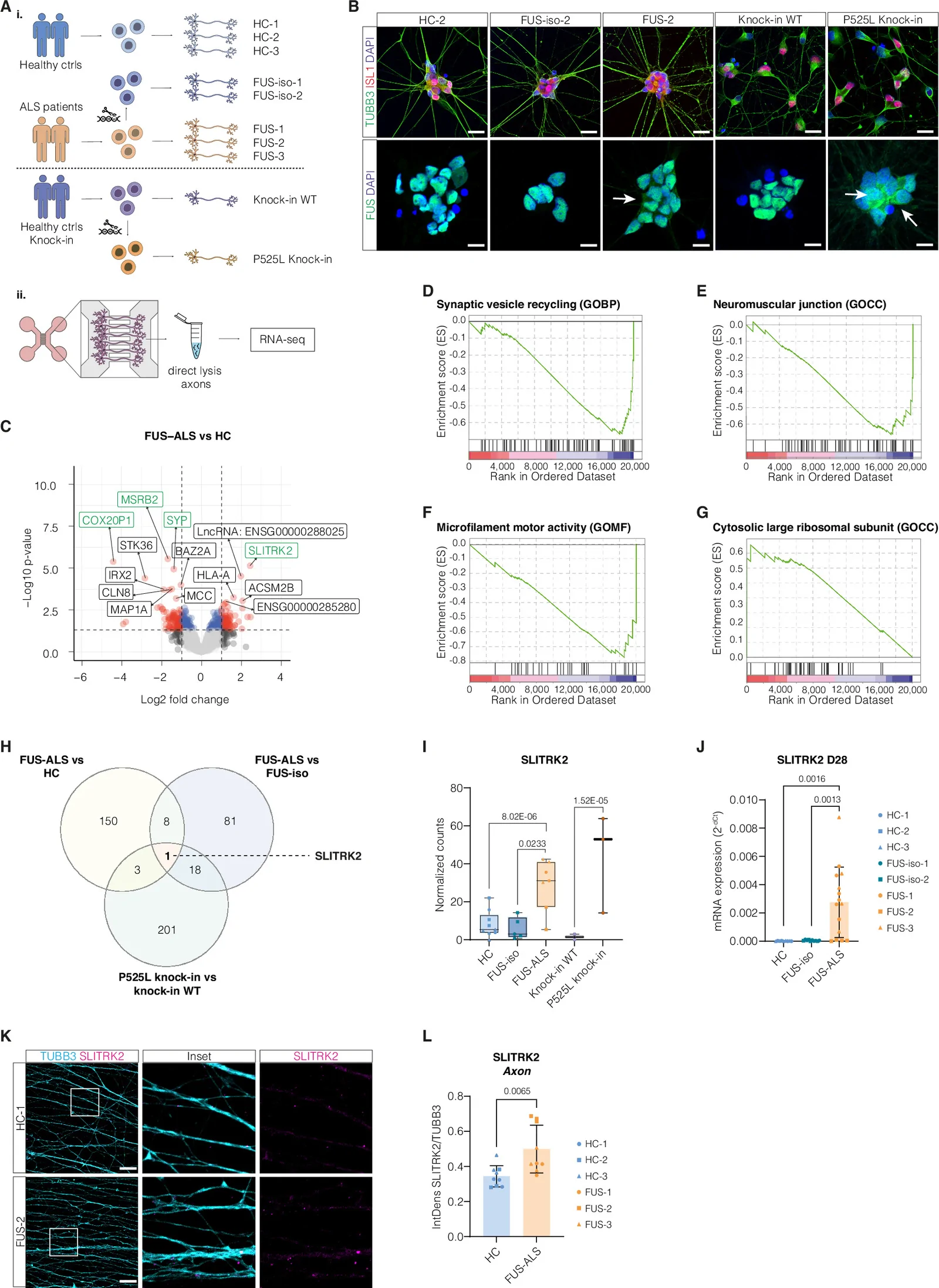
FUS-ALS mutations induce upregulation of *SLITRK2* in motor neuron axons. **A** Schematic overview of the experimental design. (i) iPSC-derived lines used in this study, including HC, FUS-ALS, FUS-iso, FUS^P525L^ knock-in and knock-in WT control lines. (ii) Schematic showing MN culture in microfluidic devices, and isolation of axonal RNA for sequencing. **B** Characterization of MN markers TUBB3 and ISL1 (upper) and FUS expression (lower) in MN derived from the iPSC lines used in this study. Arrows indicate cytoplasmic mislocalization of FUS. Scale bars: 20 μm (upper), 10 μm (lower). **C** Volcano plot showing DEGs in the axonal transcriptome of FUS-ALS compared to HC MN analyzed by DESeq2 (two-sided Wald test with Benjamini–Hochberg FDR correction; *p* < 0.05, |Log2FC | > 1). The top up- and downregulated genes are labeled. **D**–**G** Representative GSEA plots for significantly altered pathways comparing FUS-ALS vs HC MN axons. **H** Venn diagram depicting the shared DEGs identified in the three comparisons: (1) FUS-ALS versus HC, (2) FUS-ALS versus FUS-iso, and (3) FUS^P525L^ knock-in versus knock-in WT. *SLITRK2* was identified as the only commonly upregulated gene across all three analyses. **I** Box plot showing normalized read counts for *SLITRK2* in all experimental conditions. Center line: median, box bounds: 25th–75th percentiles, whiskers: min–max. Biological (*n*) and technical (t) replicates per group: HC (*n* = 3, *t* = 3,3,3), FUS-iso (*n* = 2, *t* = 2,3), FUS-ALS (*n* = 3, *t* = 2,2,3), Knock-in WT (*n* = 1, *t* = 2), P525L knock-in (*n* = 1, *t* = 3). Statistical analysis by DESeq2 (two-sided Wald test with Benjamini–Hochberg FDR correction). **J** *SLITRK2* mRNA expression levels (2^-dCT^) normalized to housekeeping genes *GAPDH* and *β-actin* determined by RT-qPCR in all experimental conditions. Data is presented as mean ± SD. HC (*n* = 2, *t* = 4,5), FUS-iso (*n* = 2, *t* = 6,4), FUS-ALS (*n* = 3, *t* = 6,4,5). One-way ANOVA performed on dCt values. **K** SLITRK2 protein expression in TUBB3 + MN axons of HC and FUS-ALS patients. The inset shows magnification of SLITRK2 protein expression. Scale bar: 20 μm. **L** Quantification of SLITRK2 expression in TUBB3 + MN axons. SLITRK2 expression was normalized to TUBB3 expression using integrated density measurements. Data is presented as mean ± SD of 3 biological replicates per group, 3 ROIs per imaged biological replicate. Unpaired two-tailed *T* test. Source data are provided as a Source Data file.

Having confirmed our ability to perform axon-seq and provide insight into MN axon gene expression and regulation, we next examined how ALS-associated mutations affect the axonal transcriptome. We exploited FUS-ALS mutations given their reported effects on gene expression, also locally, and the prominent axonal and synaptic defects found in the FUS-ALS neuromuscular system^19^. However, in contrast to previous axon-seq studies, which focused on single iPSC lines or wildtype lines carrying engineered ALS-FUS mutations^39, 50^, we generated spinal MN from three independent FUS-ALS patient-derived iPSC lines (harboring R521C, R521H and P525L mutations) (Fig. 3A and Supplementary Fig. S2A, B) and analyzed both gene expression and 3’ UTR use. Further, for two lines isogenic controls (FUS-iso) were included, in addition to three HC lines. To further validate the impact of *FUS* mutations, a HC line in which the P525L mutation was introduced, alongside its wild-type (WT) control, was included^70^ (Fig. 3A). Immunocytochemistry confirmed cytoplasmic mislocalization of FUS in *FUS* mutant, but not control, MN (Fig. 3B and Supplementary Fig. S2C).

Comparative axon-seq analysis of FUS-ALS and HC MN (*n* = 3 lines each) revealed 90 nominally significant upregulated and 102 downregulated genes in FUS-ALS as compared to HC axons (Fig. 3C, Supplementary Fig. S3A–D and Supplementary Data 2). GSEA of these DEGs (Supplementary Fig. S4A and Supplementary Data 3) revealed downregulation of pathways related to synaptic function, with *SYP* as a key downregulated gene, including *synaptic vesicle recycling* (Fig. 3C, D) and *neuromuscular junction function* (Fig. 3E). In addition, *microfilament motor activity* was also downregulated, featuring *MAP1A* as a top downregulated gene (Fig. 3C, F). Conversely, pathways associated with the *cytosolic large ribosomal subunit* were upregulated (Fig. 3G), driven by genes such as *RPL10*.

Then, to distinguish FUS-ALS mutation-driven changes from those caused by the general ALS genetic background, we first isolated and compared axonal RNA from FUS-ALS and corresponding isogenic control MN (*n* = 2). DEG analysis identified 84 nominally significant upregulated and 24 downregulated genes (Supplementary Fig. S4B and Supplementary Data 2). GSEA again revealed strong enrichment related to ribosomal function with *RPL10* as the key upregulated gene (Supplementary Fig. S4B, C). Further, a shared downregulation of neuromuscular junction-related genes was found (Supplementary Fig. S4D, E). As a second approach, MN axonal RNA from a FUS^P525L^ knock-in line (*n* = 1) and its WT control (*n* = 1) was analyzed. Comparison of the three different datasets showed limited overlap, perhaps due to the technical challenge of assessing low-count genes in axons and differences in the genetic background of the lines, but identified *SLITRK2* as a consistently upregulated gene in FUS-ALS axons (Fig. 3H, I). Interestingly, genetic variation associated with higher *SLITRK2* expression had previously been linked with the age of onset in C9ORF72-ALS patients^71^. However, if or how increased SLITRK2 levels contribute to ALS pathogenesis was not established.

SLITRK2 (Slit- and Trk-like family member 2) encodes a transmembrane protein belonging to the SLITRK family, whose expression is restricted to neural tissues^62, 72^. SLITRK2 is known to regulate neurite outgrowth^61, 62^ and synapse formation^60, 63–65^, which is interesting given the axonal and synaptic defects reported in FUS-ALS, C9ORF72-ALS and other ALS patient groups^73, 74^. First, the upregulation of *SLITRK2* was validated using RT-qPCR, showing increased levels of *SLITRK2* mRNA in FUS-ALS MN as compared to control (iso and HC; whole cell samples) (Fig. 3J). Second, to confirm that SLITRK2 protein upregulation occurs in MN axons, immunocytochemistry for SLITRK2, together with neuronal marker TUBB3, was performed on MN in microfluidic devices. The specificity of the SLITRK2 immunostaining was confirmed using different approaches (Supplementary Fig. S5A). Quantification demonstrated increased SLITRK2 protein levels in FUS-ALS axons as compared to HC (Fig. 3K, L). A trend towards SLITRK2 protein upregulation was also seen in FUS-ALS compared to FUS-iso MN axons and SLITRK2 was similarly increased in the somatodendritic compartment (Supplementary Fig. S5B–E). Thus, although the level of SLITRK2 mRNA increase was somewhat variable at the whole-cell level (Fig. 3J), robust upregulation of SLITRK2 protein was detected in the axonal and somatodendritic compartments (Fig. 3K, L and Supplementary Fig. 5D, E). Together, these results identify consistent and robust upregulation of SLITRK2 in ALS MN axons.

### Changes in *SNCA* and *SLITRK2* isoforms in FUS-ALS motor neurons

**Fig. 4:**
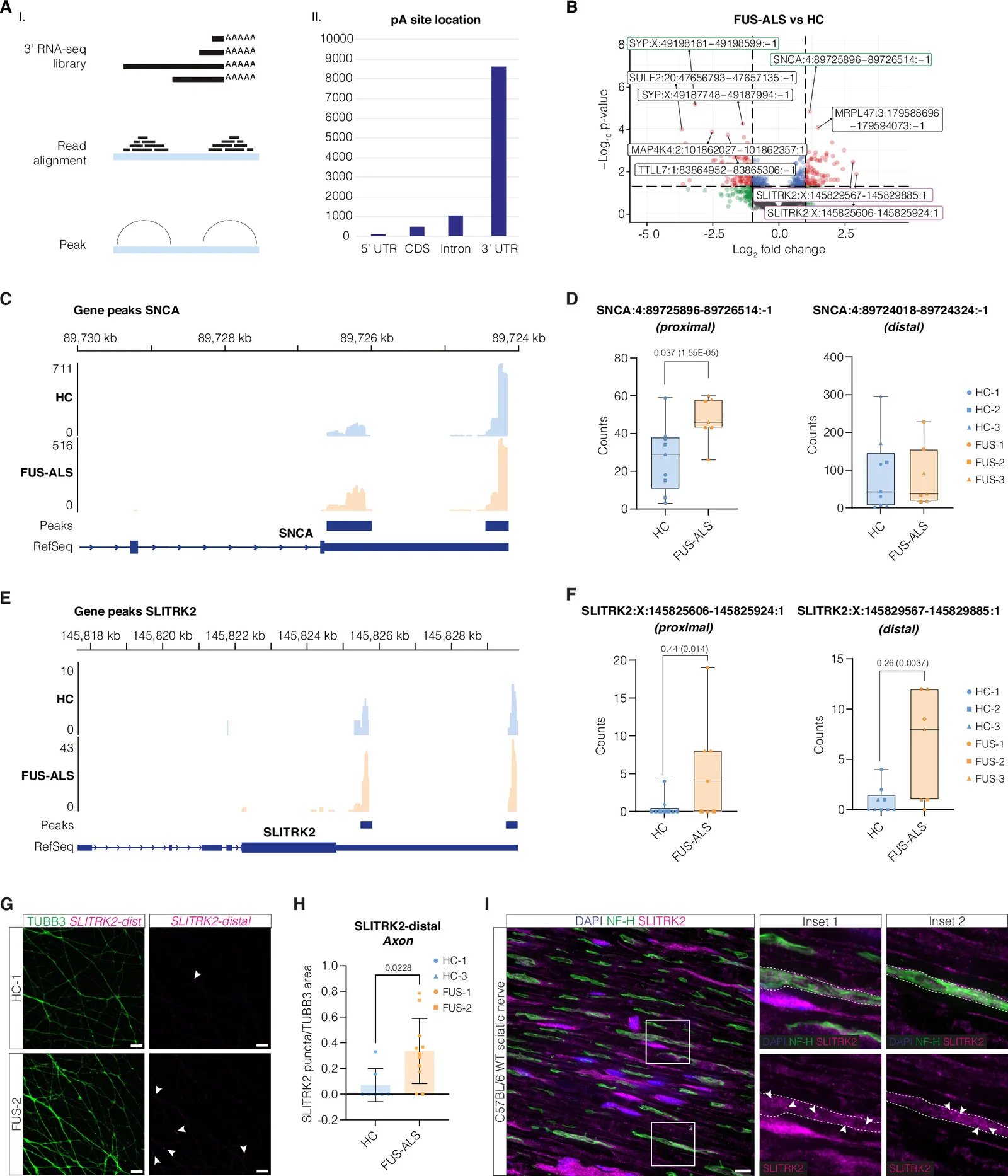
Changes in *SNCA* and *SLITRK2* isoforms in FUS-ALS motor neurons. **A** (i) Schematic of 3′ RNA-seq polyadenylation site (pA) identification via peak calling. (ii) Genomic distribution of identified gene peaks (3′ UTR, CDS, intron, 5′ UTR; CDS = coding sequence). **B** Volcano plot showing differential usage of 3’ UTR isoforms comparing the axonal transcriptome of FUS-ALS versus HC MN analyzed by DESeq2 (two-sided Wald test with Benjamini–Hochberg FDR correction; *p* < 0.05, |Log2FC | > 1). Top up- and downregulated gene peaks are labeled; gene peaks with *p*adj < 0.05 are highlighted in green. *SLITRK2* peaks are indicated in magenta. **C** IGV visualization of *SNCA* gene peaks mapped to RefSeq-Select annotation in FUS-ALS and HC MN axons, demonstrating short (proximal pA site usage) and long (distal pA site usage) 3’ UTR isoforms. **D** Boxplot showing counts of proximal (*SNCA:4:89725896-89726514:−1*) and distal (*SNCA:4:89724018-89724324:−1*) *SNCA* gene peaks in HC and FUS-ALS patient MN axons. Center line: median, box bounds: 25th–75th percentiles, whiskers: min–max. Biological (*n*) and technical (*t*) replicates per group: HC (*n* = 3, *t* = 3,3,3), FUS-ALS (*n* = 3, *t* = 2,2,3). Statistical analysis using DESeq2 (two-sided Wald test with Benjamini–Hochberg FDR correction). *p*-values with adjusted *p*-values in brackets. **E** IGV visualization of *SLITRK2* gene peaks mapped to RefSeq-Select annotation in FUS-ALS and HC MN axons, demonstrating short (proximal) and long (distal) 3’ UTR isoforms. **F** Boxplot showing counts of proximal (*SLITRK2:X:145825606-145825924:1*) and distal (*SLITRK2:X:145829567-145829885:1*) *SLITRK2* gene peaks in HC and *FUS*-ALS patient MN axons. Boxplot elements, replicates, and DESeq2 statistics are as described in (**D**). **G** Representative RNAscope images of distal (long) SLITRK2 3’ UTR isoform within TUBB3 + MN axons from HC and FUS-ALS lines. Scale bar: 20 μm. **H** Quantification of distal (long) *SLITRK2* isoform expression in HC and FUS-ALS MN axons, normalized to the TUBB3+ area. Data is presented as mean ± SD. HC (*n* = 2, *t* = 4,3), FUS-ALS (*n* = 2, *t* = 3,8). Two-tailed Mann-Whitney test. **I** Immunofluorescence of SLITRK2 Neurofilament H (NF-H), and DAPI (nuclei) expression in WT (C57BL/6) mouse sciatic nerve tissue. Insets show higher magnifications, with arrows highlighting SLITRK2 expression within NF-H + axons. Scale bar: 10 μm. Source data are provided as a Source Data file.

How ALS gene mutations affect 3’ UTR isoforms in MN, and especially in specific cellular compartments, remains largely unknown. As our analysis of axon-seq data from HC MN highlighted compartment-specific isoform expression (Fig. 2F–I), we next analyzed 3’ UTR isoforms in FUS-ALS MN, with an emphasis on key dysregulated genes such as *SLITRK2*. This analysis detected 9169 3’ UTR peaks originating from 6968 genes (Fig. 4A). To specifically focus on APA sites within genes, subsequent differential expression analysis considered 1942 genes exhibiting more than one distinct peak (4749 3’ UTR peaks in total). Comparison of FUS-ALS and HC axon samples showed 84 nominally significant upregulated and 135 downregulated 3’ UTR peaks (Fig. 4B and Supplementary Data 4). These peaks were detected in genes such as *SYP* (Supplementary Fig. S6A, B), which was significantly downregulated after FDR adjustment in FUS-ALS axons (Fig. 3C), and *SNCA* (α-synuclein)(Fig. 4C, D). Previous studies have linked *SNCA* isoforms with extended 3’ UTRs to pathological processes and common variants in these isoforms are associated with an increased risk of Parkinson’s Disease^75^. Our data revealed two *SNCA* peaks: a proximal peak corresponding to a shorter 3’ UTR isoform and a distal peak representing a longer isoform (Fig. 4C). The long isoform was predominantly expressed in HC MN axonal and somatodendritic compartments, with no ratio differences between axonal and somatodendritic compartments (Supplementary Fig. 6C, D). Interestingly, a significant upregulation of the proximal (shorter) isoform peak was detected in FUS-ALS as compared to HC axons, while the long isoform remained unchanged (Fig. 4D).

To next assess mutation-specific effects, FUS-ALS and FUS-iso 3’ UTR isoform data were generated, and their comparison identified 51 nominally significant upregulated and 26 downregulated peaks (Supplementary Fig. 6E and Supplementary Data 4). Intersecting these data with the results from the FUS-ALS versus HC comparison identified commonly dysregulated isoforms involved in synaptic function (*SLITRK2, NETO2*) and cytoskeleton and cell structure (*SEPTIN11, FARP1, TUBB4A, TTL*)(Supplementary Fig. 6F). Interestingly, changes in two peaks in *SLITRK2* exhibited the largest positive LFC difference in the FUS-ALS versus HC axon comparison (Fig. 4B and Supplementary Data 4). These peaks correspond to the known short (proximal polyadenylation site) and long (distal polyadenylation site) *SLITRK2* transcript isoforms (Fig. 4E, F). Analysis of *SLITRK2* transcript isoform expression in HC MN showed that both isoforms were predominantly expressed in soma compared to axons, although these differences did not reach statistical significance (Supplementary Fig. 6G, H). No difference in the ratio of the two isoforms between the somatodendritic and axonal compartments was found. Overall, these data identify compartment-specific changes in 3’ UTR isoform abundance for several transcripts in FUS-ALS MN.

To validate the observed upregulation of *SLITRK2* in FUS-ALS MN, RNAscope was performed to detect the long isoform (Fig. 4G). This analysis confirmed the presence of the long *SLITRK2* isoform in MN axons and its significant upregulation in FUS-ALS as compared to HC axons (Fig. 4H). Increased expression of the long isoform was also detected in the somatodendritic compartment (Supplementary Fig. 6I, J). To confirm the expression of SLITRK2 in motor axons in vivo, immunohistochemistry for SLITRK2 was performed on sections of the sciatic nerve of adult C57BL/6 WT mice (Fig. 4I). This showed SLITRK2 labeling of NF-H-positive motor axons, confirming expression of SLITRK2 in the axonal compartment.

### SLITRK2 knockdown rescues axonal deficits in FUS-ALS motor neurons

**Fig. 5:**
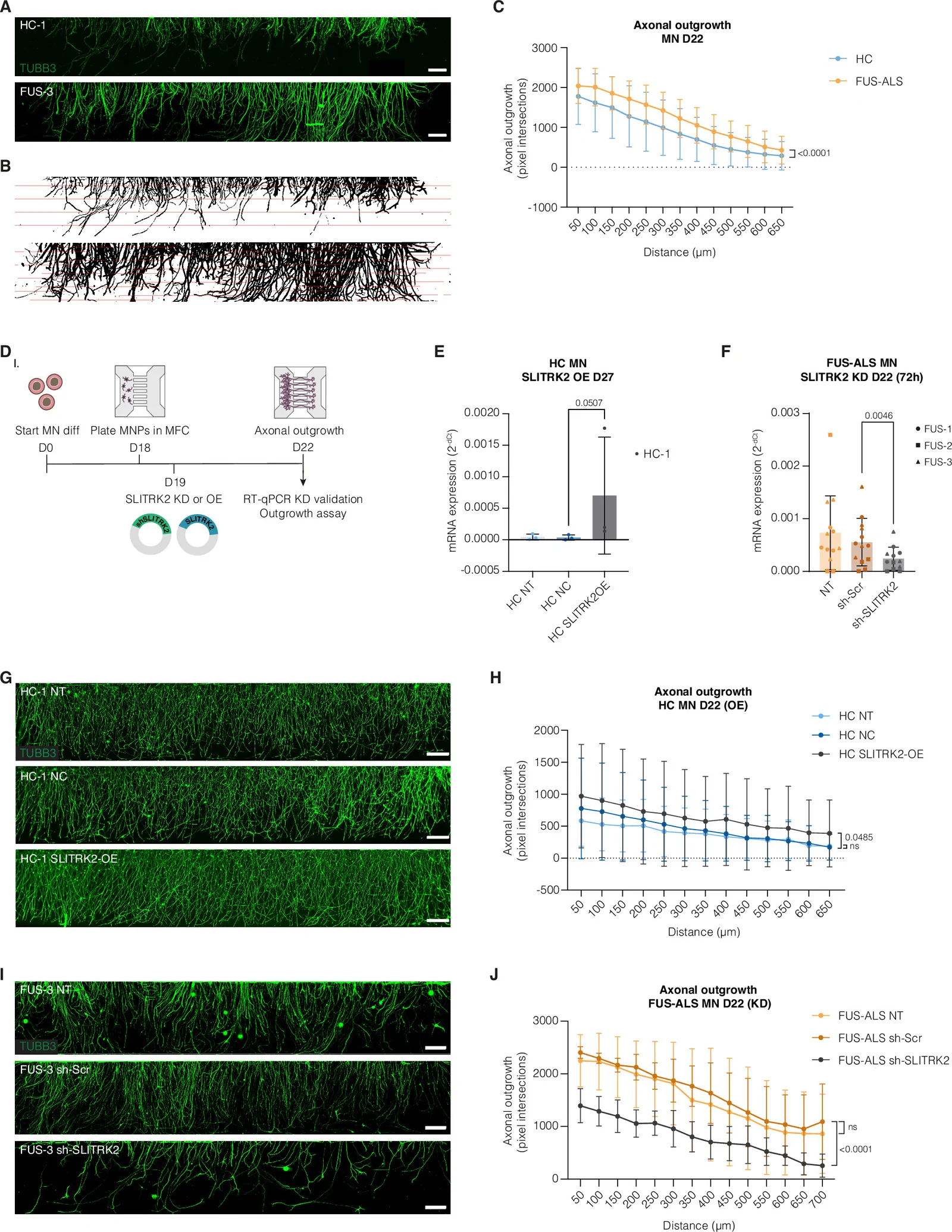
SLITRK2 knockdown rescues axonal deficits in FUS-ALS motor neurons. **A** Representative images of axonal outgrowth in the distal compartment of HC and FUS-ALS MN visualized by immunocytochemical staining for TUBB3. Scale bar: 200 μm. **B** Masks used for linear Sholl analysis of TUBB3 immunostaining with intersection lines at 50 μm intervals, starting from the microgroove exit. **C** Quantifications of axonal outgrowth, measured as the number of pixel intersections with Sholl intersection lines every 50 μm, in HC and FUS-ALS MN. Data is presented as mean ± SD (*n* = 3 biological replicates, *t* = 3 technical replicates). Paired two-tailed T-test. **D** Schematic overview of SLITRK2 knockdown (KD) or overexpression (OE) in iPSC-derived MN cultures. **E** *SLITRK2* mRNA expression levels (2^-dCT^) in non-transduced (NT), negative control (NC), and SLITRK2-OE treated HC MN at D27. Data is presented as mean ± SD (*n* = 1, *t* = 3). Kruskal-Wallis test. **F** *SLITRK2* mRNA expression levels (2^-dCT^) in non-transduced (NT), sh-Scr treated, and sh-SLITRK2 treated FUS-ALS MN at D22. Data is presented as mean ± SD (*n* = 3, *t* = 4). Mixed effect analysis. **G** Representative images of axonal outgrowth in the distal compartment of NT, NC, and SLITRK2-OE treated HC MN at D22, visualized by immunocytochemical staining for TUBB3. Scale bar: 200 μm. **H** Quantification of axonal outgrowth, measured as the number of pixel intersections with Sholl intersection lines every 50 μm, in NT, NC and SLITRK2-OE treated HC MN at D22. Data is presented as mean ± SD (*n* = 3, *t* = 1). Kruskal-Wallis test. **I** Representative images of axonal outgrowth in the distal compartment of NT, sh-Scr treated, and sh-SLITRK2 treated FUS-ALS MN at D22, visualized by immunocytochemical staining for TUBB3. Scale bar: 200 μm. **J** Quantification of axonal outgrowth, measured as the number of pixel intersections with Sholl intersection lines every 50 μm, in NT, sh-Scr treated, and sh-SLITRK2 treated FUS-ALS MN at D22. Data is presented as mean ± SD (*n* = 3, *t* = 1). One-Way ANOVA. Source data are provided as a Source Data file.

Given the consistent SLITRK2 upregulation in FUS-ALS axons (Fig. 3, 4) and its role in neurite outgrowth^61, 62^, we investigated the link between SLITRK2 dysregulation and axonal changes. Axonal outgrowth was assessed in compartimentalized HC, FUS-iso, and FUS-ALS MN cultures using linear Scholl analysis^36^. This revealed significantly increased axonal outgrowth in FUS-ALS MN compared to both HC and isogenic controls (Fig. 5A–C and Supplementary Fig. 7A–C), in line with previous work^37, 39^.

Next, the effect of SLITRK2 knockdown (KD) or overexpression (OE) on axonal outgrowth in HC MN was examined. HC MN were transduced with either SLITRK2-OE or sh-SLITRK2 lentiviral vectors at D19 and assayed for axonal outgrowth at D22 (Fig. 5D). SLITRK2 OE resulted in a significant increase of SLITRK2 levels as compared to negative control (NC) treated HC MN (Fig. 5E). SLITRK2 shRNAs, designed to target the proximal part of *SLITRK2* 3’ UTR region to ensure KD of both isoforms (Supplementary Fig. 7D), induced a significant reduction of *SLITRK2* levels as compared to scrambled control (sh-Scr) (Fig. 5F). KD of SLITRK2 in HC MN did not affect axonal outgrowth (Supplementary Fig. S7E, F), consistent with its low baseline expression. In contrast, SLITRK2 OE was sufficient to significantly increase axonal outgrowth (Fig. 5G, H), in line with its reported role in neurite growth.

Finally, to examine whether the increased levels of SLITRK2 contribute to enhanced axonal growth in FUS-ALS MN, lentiviral KD of SLITRK2 was performed in FUS-ALS MN. Remarkably, KD of SLITRK2 in FUS-ALS MN completely rescued the enhanced axonal outgrowth phenotype (Fig. 5I, J). These results show that increased SLITRK2 expression induces axonal phenotypes in FUS-ALS MN.

### Development of a iNMJ-on-a-chip model

In addition to regulating neurite growth, SLITRK2 controls synapse formation and function^60^. Changes in SLITRK2 expression in FUS-ALS MN may therefore affect the synapse between MN and skeletal muscle, the NMJ. To test this hypothesis, we first developed an MN-myotube co-culture in a microfluidic system. While previous work studying ALS phenotypes in microfluidic neuromuscular systems employed rodent-derived muscle cells^76–78^ or human primary skeletal myoblasts^51, 54, 55^, mesoangioblasts^36, 53^ or mesenchymal stem cells^52^, we set up a protocol to generate a fully iPSC-derived model by combining iPSC-derived MN and iPSC-derived myotubes. To generate multinucleated myofibers from iPSCs, myogenic progenitor purification was performed by adapting a previously published, transgene-free protocol^56, 79^. These purified progenitors were then differentiated into multinucleated skeletal muscle cells^56–58^ (Supplementary Fig. S8A). These cells expressed the early myogenic marker *MyoD* and showed a temporal increase in the expression of late developmental and mature SkM markers (Supplementary Fig. S8B). Further, expression of the adult MyHC isoforms *MYH1*, *MYH2*, and *MYH7*, characteristic of fast- and slow-twitch fibers respectively^80^, was strongest at D12. Immunocytochemistry confirmed the presence of multinucleated myotubes expressing various MYH isoforms (MF20 antibody) and the sarcomeric component α-actinin (Supplementary Fig. S8C). Furthermore, these myotubes exhibited spontaneous contractions (Supplementary Video S1), indicating the formation of functional sarcomeres and terminal differentiation.

**Fig. 6:**
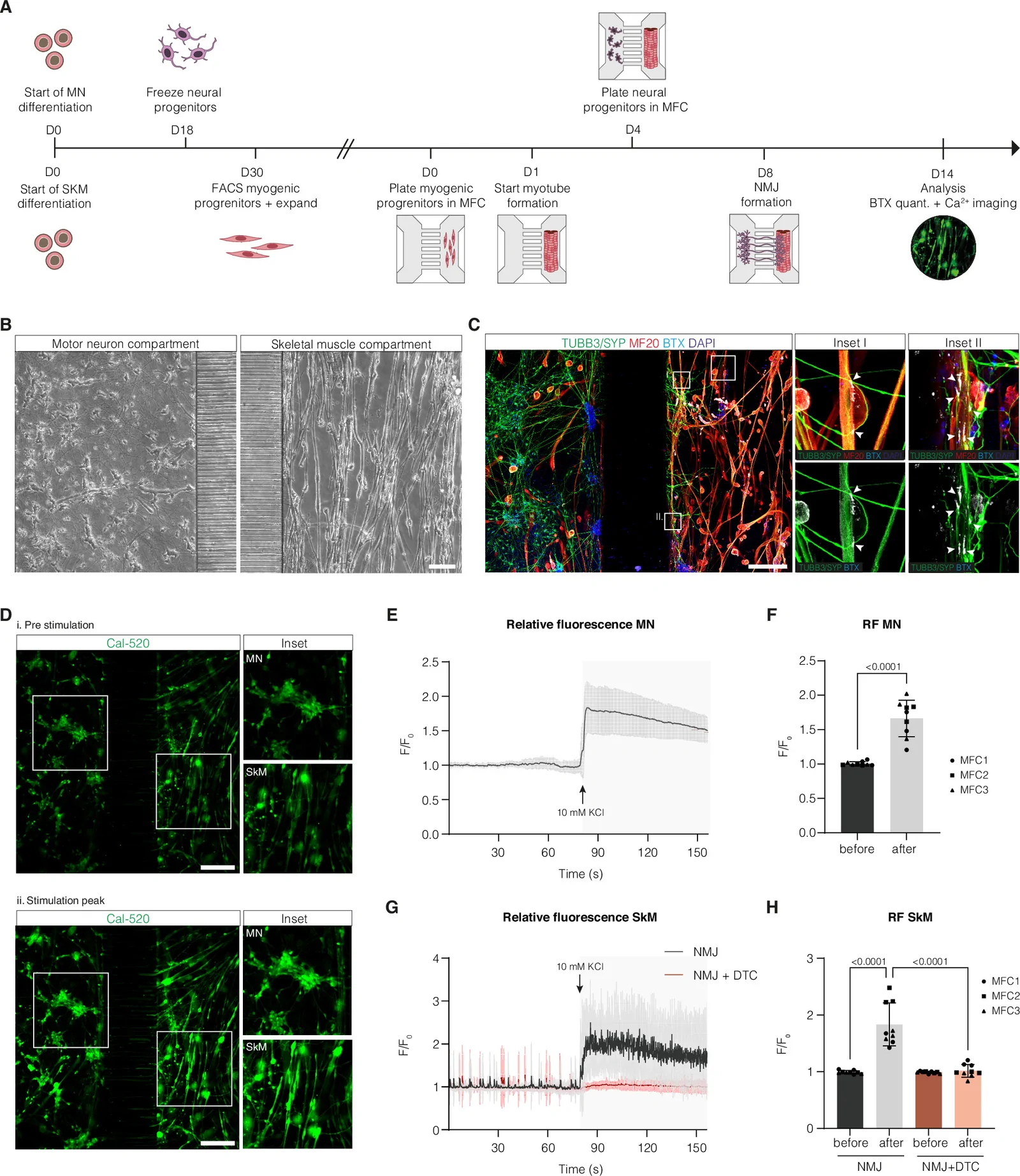
Development of a iNMJ-on-a-chip model. **A** Schematic overview of the co-culture protocol and differentiation timeline. iPSCs are differentiated into MN progenitors and myogenic progenitors in parallel and frozen after respectively 18 days and 30+ days in culture. At D0, myogenic progenitors are seeded in one side of the microfluidic device. Myotube formation is initiated the day after. At D4, MN progenitors are seeded on the opposite side, spinal MN differentiation is directly initiated. After 4 days in co-culture, axons reach the myotube compartment via the microchannels and NMJ formation is initiated. MN and myotubes are cultured together for 10 days. **B** Brightfield picture of representative NMJ-on-a-chip culture, left: MN compartment; right: skeletal muscle compartment. Scale bar: 100 μm. **C** Confocal images of the iNMJ-on-a-chip showing MN including presynapse in green (TUBB3/SYP), skeletal muscle in red (MF20) and NMJs in gray (BTX). Insets: magnification of NMJ formation with arrows indicating colocalization of pre- (TUBB3/SYP) and postsynaptic (BTX) markers. Scale bar: 200 μm. **D** Live imaging pictures of intracellular Ca^2+^ in MN and skeletal muscle before (i.) and during (ii.) stimulation with 10 mM KCl. Insets show magnification of unstimulated (i.) and stimulated (ii.) MN and skeletal muscle. Scale bar: 200 μm. **E** Relative fluorescence of Ca^2+^ intensity in healthy MN before and after (gray box) stimulation with 10 mM KCl (arrow). **F** Quantification of relative fluorescence in MN before and after stimulation with 10 mM KCl. **G** Relative fluorescence of Ca^2+^ intensity in healthy skeletal muscle before and after (gray box) stimulation with 10 mM KCl (arrow) and after 10 min treatment with NMJ blocker DTC. **H** Quantification of relative fluorescence in skeletal muscle before and after MN stimulation with 10 mM KCl and without or with treatment with DTC. * All data in E-H is presented as mean ± SD (*n* = 3 biological replicates, *t* = 3 technical replicates). F: Paired two-tailed *T* test, H: Kruskal-Wallis test. Source data are provided as a Source Data file.

To establish co-cultures, iPSCs were differentiated in parallel into MN (until D18 of the differentiation protocol) or myogenic progenitors (Fig. 6A). Myogenic progenitors were plated in the muscle (distal) compartment of the microfluidic device (Supplementary Fig. S9A) and allowed to fuse for 3 days. Subsequently, MN were plated in the proximal compartment. By D8 after plating, myotube contractions were observed, indicating successful innervation by MN. Co-cultures were maintained until D13 of the myotube maturation protocol and D28 of the MN differentiation protocol (Fig. 6A, B). NMJ formation was confirmed by colocalization of presynaptic markers (TUBB3 and SYP) and α-bungarotoxin (BTX)-positive AChR clusters on myotubes (Fig. 6C) and by live-cell calcium (Ca^2+^) imaging. iPSC-derived MN and myotubes from the same donor were co-cultured and loaded with the Ca^2+^-sensitive dye Cal-520 at D14. MN stimulation with 10 mM KCl induced strong Ca²⁺ signals in MN (Fig. 6D–F) and downstream myotubes (Fig. 6D, G, H). These results supported functional synaptic transmission at the NMJ, which was confirmed by the ability of d-tubocurarine (DTC), a competitive nicotinic AChR antagonist, to block induced myotube Ca²⁺ responses (Fig. 6G, H) without affecting MN activation (Supplementary Fig. S9B, C) or spontaneous Ca²⁺ activity in myotube monocultures (Supplementary Fig. S9D, E).

Together, these results demonstrate our ability to generate fully iPSC-derived, functional NMJs in compartmentalized devices, with MN activity driving myotube responses.

### SLITRK2 knockdown rescues impaired neuromuscular transmission in FUS-ALS co-cultures

**Fig. 7:**
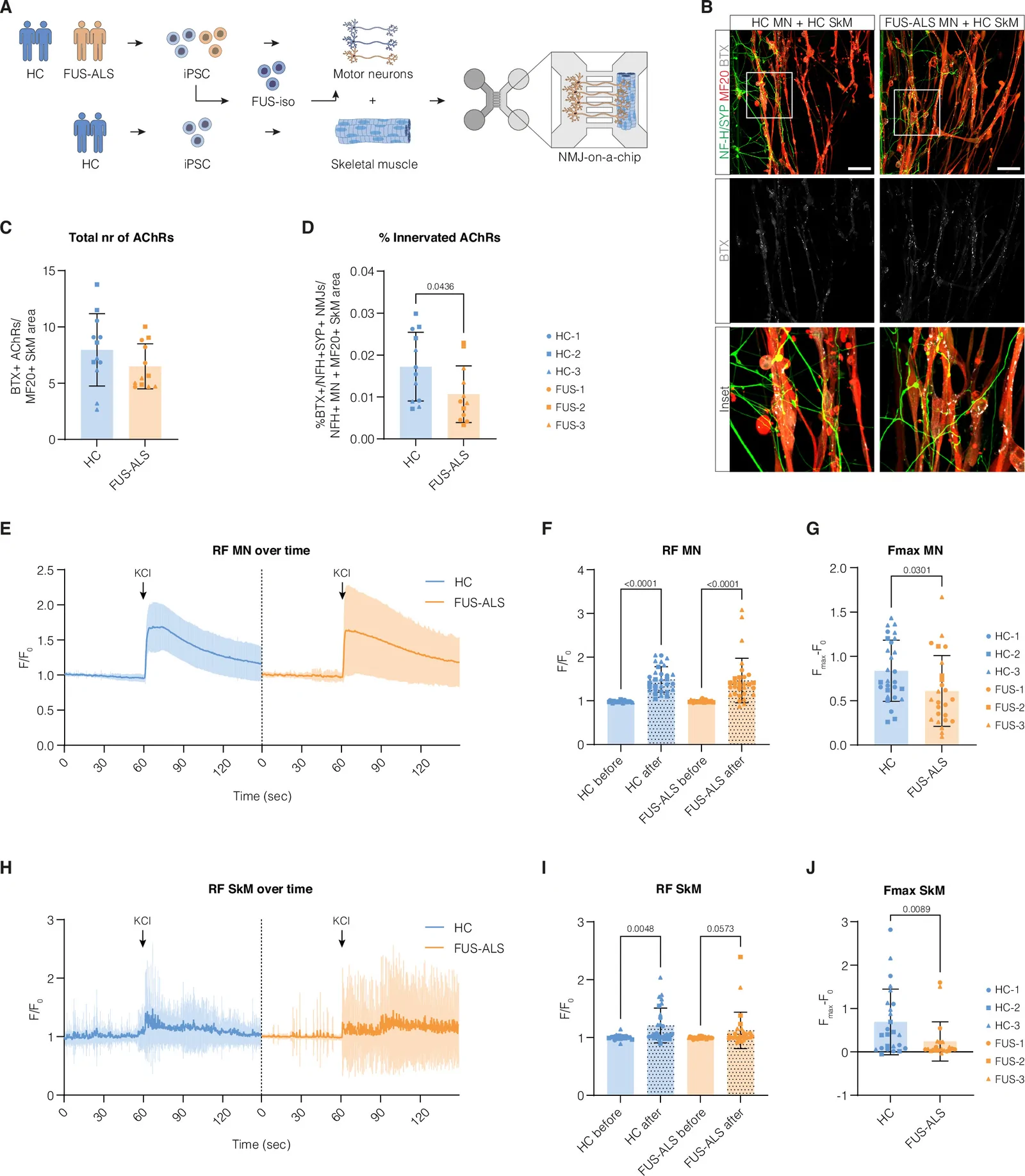
FUS-ALS co-cultures exhibit impaired NMJ integrity and function. **A** Schematic of our iNMJ-on-a-chip co-culture setup combining HC, FUS-iso or FUS-ALS iPSC-derived MN with HC iPSC-derived skeletal muscle to assess NMJ formation and function. **B** Immunofluorescence images of HC (left) or FUS-ALS (right) MN co-cultured with HC myotubes. MN are positive for NF-H and SYP (green), myotubes for MF20 (magenta) and AChRs for BTX (cyan). Insets highlight NMJs as defined by BTX and NF-H-SYP colocalization. Scale bar: 100 μm. **C** Quantification of total AChRs count in HC myotubes co-cultured with HC or FUS-ALS MN, normalized to the total % area of MF20. Data is presented as mean ± SD (*n* = 3 biological replicates, *t* = 4 technical replicates). Two-tailed Mann-Whitney test (*p* = 0.1432). **D** Quantification of innervated NMJs (BTX/NF-H/SYP colocalizations) in HC and FUS-ALS MN co-cultures with HC myotubes, normalized to the sum of the total % area of NF-H/SYP and MF20. Data is presented as mean ± SD (*n* = 3, *t* = 4). Unpaired two-tailed T-test. **E** Relative fluorescence (*F*/*F*_0_) of Ca^2+^ intensity over time in HC and FUS-ALS MN co-cultured with HC myotubes, before and after MN stimulation with 10 mM KCl (arrow). Data is presented as mean ± SD (*n* = 3, *t* = 4). **F** Quantification of relative fluorescence (*F*/*F*_0_) in HC and FUS-ALS MN before and after MN stimulation. Data is presented as mean ± SD. HC (*n* = 3, *t* = 9,9,12), FUS-ALS (*n* = 3, *t* = 12,6,12). Kruskal-Wallis test. **G** Quantification of *F*max (*F*_max_-*F*_0_) in HC and FUS-ALS MN co-cultured with HC myotubes. Data is presented as mean ± SD. HC (*n* = 3, *t* = 9,7,12), FUS-ALS (*n* = 3, *t* = 12,3,10). Unpaired two-tailed T-test. **H** Relative fluorescence (*F*/*F*_0_) of Ca^2+^ intensity over time in HC myotubes co-cultured with HC and FUS-ALS MN, before and after MN stimulation with 10 mM KCl (arrow). Data is presented as mean ± SD (*n* = 3, *t* = 4). **I** Quantification of relative fluorescence (*F*/*F*_0_) in HC myotubes co-cultured with HC and FUS-ALS MN. Data is presented as mean ± SD. HC (*n* = 3, *t* = 9,7,12), FUS-ALS (*n* = 3, *t* = 12,3,12). Unpaired two-tailed T-test. Kruskal-Wallis test. **J** Quantification of Fmax (*F*_max_-*F*_0_) in HC myotubes co-cultured with HC and FUS-ALS MN. Data is presented as mean ± SD. HC (*n* = 3, *t* = 9,7,9), FUS-ALS (*n* = 2, *t* = 11,10). Two-tailed Mann-Whitney test. Source data are provided as a Source Data file.

To test the consequence of elevated SLITRK2 at the level of the NMJ, we first assessed whether FUS mutations affect NMJ integrity in the iNMJ-on-a-chip model, as reported in other models^34, 36^. FUS-ALS, or control MN, were co-cultured with HC iPSC-derived myotubes (Fig. 7A). No significant differences in the number of BTX-positive AChR clusters on myotubes in FUS-ALS and HC or FUS-iso cultures were detected (Fig. 7B, C and Supplementary Fig. S10A, B). To quantify NMJ numbers, co-localized NMJs were normalized to total neuronal and myotube area to compensate for axonal outgrowth and the number of myotubes. This analysis showed that the percentage of innervated NMJs was reduced in FUS-ALS as compared to HC cultures (Fig. 7D and Supplementary Fig. S10C). Interestingly, the enhanced axonal outgrowth phenotype seen in FUS-ALS mono-cultures (Fig. 5) was absent in NMJ co-cultures (Supplementary Fig. S10D–G). To assess whether these structural changes lead to impaired neuromuscular communication, live-cell Ca^2+^ imaging was peformed. KCl stimulation elicited Ca^2+^ transients and a significant increase in the relative fluorescence (RF) of the Ca^2+^ intensity in MN across all conditions (Fig. 7E, F and Supplementary Fig. S11A, B). However, the peak Ca^2+^ response (*F*max) was significantly reduced in FUS-ALS compared to HC MN (Fig. 7G and Supplementary Fig. S11C). This suggests that FUS-ALS MN are hypo-excitable, as reported previously for FUS-ALS MN in other models^81–83^. In HC co-cultures, evoked MN activity triggered corresponding Ca^2+^ transients in myotubes (Fig. 7H, I and Supplementary Fig. 11D, E). However, the response to MN activation in HC myotubes was impaired in the presence of FUS-ALS MN, indicated by a lower *F*max in the myotubes (Fig. 7I). A similar, but non-significant, reduction in postsynaptic activation was also observed in comparison to FUS-iso co-cultures (Supplementary Fig. 11F). These results show that *FUS* mutations impair both NMJ formation and function, leading to reduced synaptic connectivity and neuromuscular transmission.

**Fig. 8:**
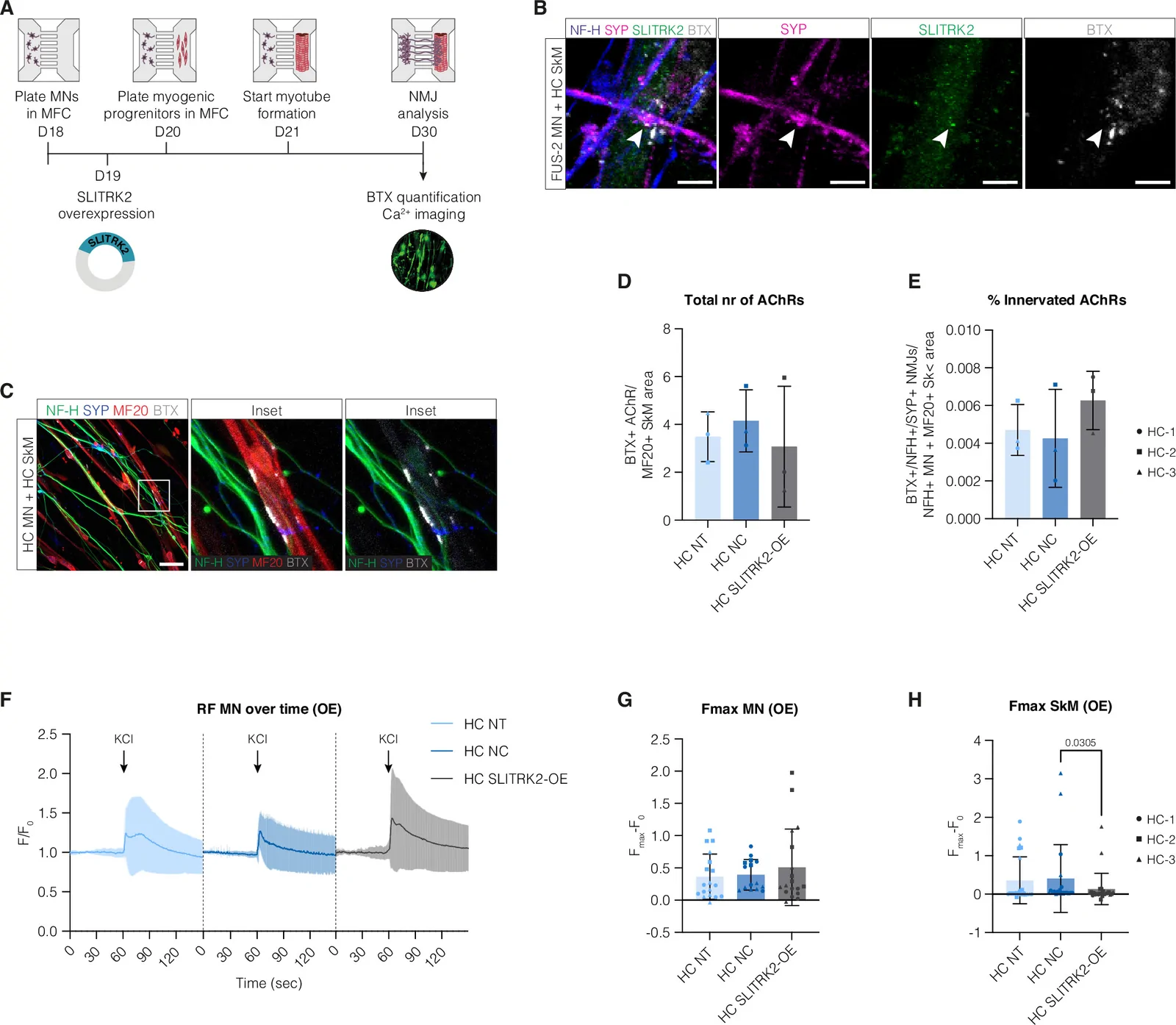
SLITRK2 overexpression in HC MN recapitulates FUS-ALS-associated synaptic deficits. **A** Schematic of SLITRK2 overexpression (OE) experiments in iNMJ-on-a-chip cultures. HC MN were transduced with SLITRK2-OE lentivirus at D19 of differentiation. HC myogenic progenitors were plated into adjacent compartments at D20, and subsequently differentiated into myotubes. NMJ function was assessed at D30. **B** Representative immunofluorescence staining for SLITRK2, NF-H, SYP and BTX in FUS MN with HC SkM co-cultures. Arrows point to SLITRK2 expression at the NMJ (SYP + /BTX + ). Scale bar: 5 μm. **C** Immunofluorescence images of HC MN with HC myotubes co-cultures. MN are positive for NF-H (green) and SYP (blue), myotubes for MF20 (red) and AChRs for BTX (gray). Inset highlights NMJs defined by BTX, NF-H and SYP colocalization. Scale bar: 50 μm. **D** Quantification of total AChRs count in co-cultures of NT, NC, and SLITRK2-OE treated HC MN with HC myotubes, normalized to the total % area of MF20. Data is presented as mean ± SD (*n* = 3 biological replicates, *t* = 1 technical replicate). One-Way ANOVA (*p* = 0.7565). **E** Quantification of innervated NMJs (BTX/NF-H/SYP colocalizations) in NT, NC treated, and SLITRK2-OE treated HC MN co-cultures with HC myotubes, normalized to the sum of the total % area of NF-H and MF20. Data is presented as mean ± SD (*n* = 3, *t* = 1). One-Way ANOVA (*p* = 0.4491). **F** Relative fluorescence (*F*/*F*_0_) of Ca^2+^ intensity over time in NT, NC and SLITRK2-OE treated HC MN co-cultures with HC myotubes, before and after MN stimulation with 10 mM KCl (arrow). Data is presented as mean ± SD. **G** Quantification of *F*max (*F*_max_-*F*_0_) in NT, NC and SLITRK2-OE treated HC MN co-cultured with HC myotubes. Data is presented as mean ± SD. HC (*n* = 3, *t* = 6,6,6), HC NC (*n* = 3, *t* = 6,3,6), HC SLITRK2 OE (*n* = 3, *t* = 6,6,6). Kruskal-Wallis test (*p* = 0.6504). **H** Quantification of *F*max (*F*_max_-*F*_0_) in HC myotubes co-cultured with NT, NC treated, and SLITRK2-OE treated HC MN. Data is presented as mean ± SD. HC (*n* = 3, *t* = 7,8,8), HC NC (*n* = 3, *t* = 8,4,8), HC SLITRK2 OE (*n* = 3, *t* = 8,8,8). Kruskal-Wallis test. Source data are provided as a Source Data file.

As a first step to determine whether enhanced SLITRK2 expression may contribute to neuromuscular dysfunction in FUS-ALS, HC MN were transduced with a lentiviral vector expressing SLITRK2, and co-cultured with HC myogenic progenitors (Fig. 8A). In line with our data showing SLITRK2 expression in MN axons (Fig. 3I, K, L; Fig. 4E–H), immunohistochemistry detected SLITRK2 at the presynapse of NMJs in MN-HC myotube co-cultures (Fig. 8B). Structural analysis of the NMJ, by immunostaining for NF-H, SYP and BTX, did not reveal differences in NMJ number or innervation upon SLITRK2 OE (Fig. 8C–E). Viral vector-induced expression of SLITRK2 in MN did not alter the intrinsic excitability of MN (Fig. 8F, G and Supplementary Fig. S12A), but significantly reduced postsynaptic myotube activation upon MN stimulation (Fig. 8H and Supplementary Fig. S12B, C). This recapitules the transmission defects observed in FUS-ALS cultures.

**Fig. 9:**
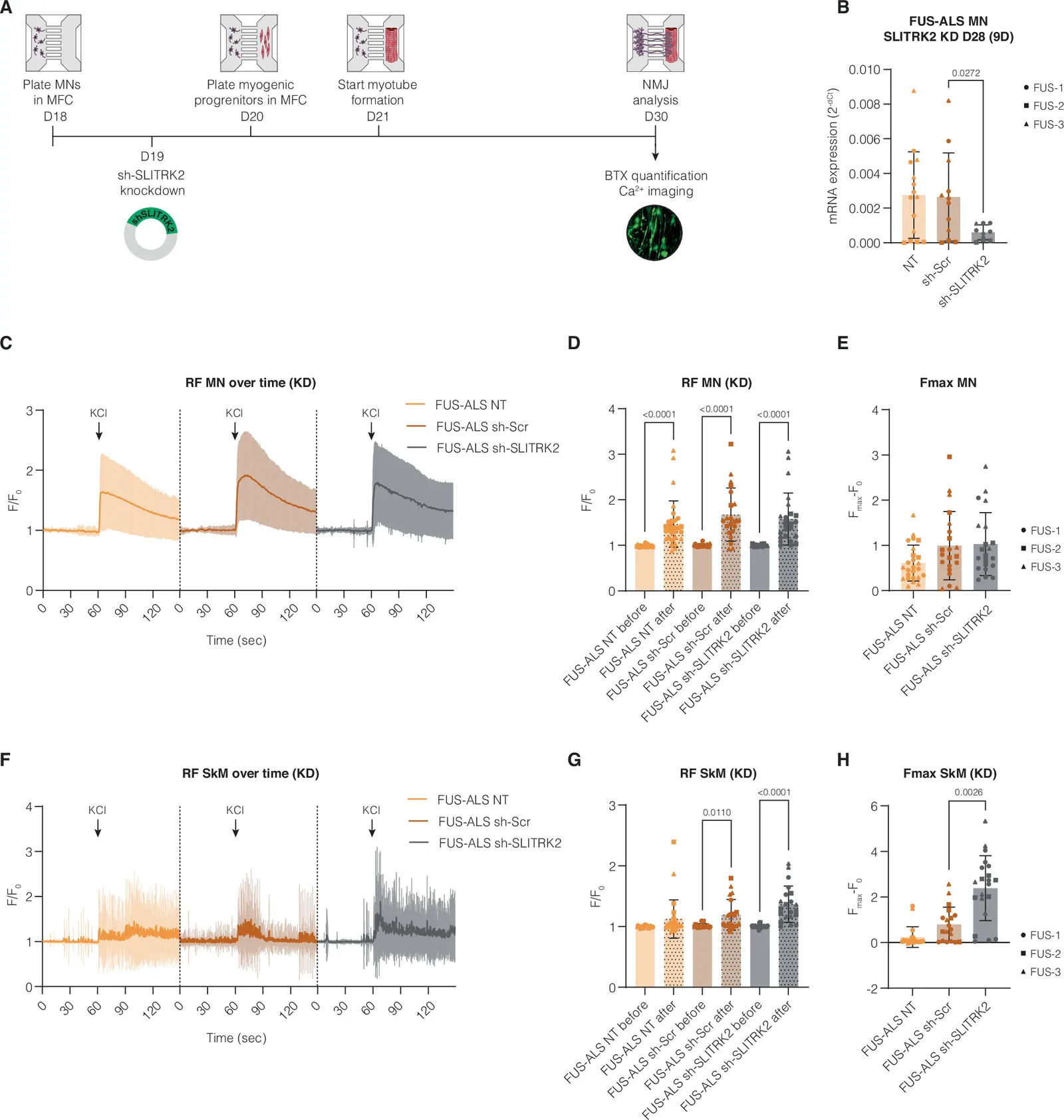
SLITRK2 knockdown rescues impaired neuromuscular transmission in FUS-ALS co-cultures. **A** Schematic overview of the SLITRK2 knockdown (KD) rescue experiment in iNMJ-on-a-chip cultures. FUS-ALS MN were treated with lentivirus expressing sh-SLITRK2 or sh-Scr one day after plating in microfluidics devices (at D19 of differentiation). HC myogenic progenitors were plated into the adjacent compartment at D20, and subsequently differentiated into myotubes. NMJ function was assessed at D30 of the MN differentiation protocol. **B** *SLITRK2* mRNA expression levels (2^-dCT^) in NT, sh-Scr treated, and sh-SLITRK2 treated FUS-ALS MN at D28. Data is presented as mean ± SD. Biological (*n*) and technical (*t*) replicates per group: FUS-ALS (*n* = 3, *t* = 6,4,5), FUS-ALS scr (*n* = 3, *t* = 5,3,4), FUS-ALS shSLITRK2 (*n* = 3, *t* = 4,3,3). Mixed effect analysis. **C** Relative fluorescence (*F*/*F*_0_) of Ca^2+^ intensity over time in NT, sh-Scr treated, and sh-SLITRK2 treated FUS-ALS MN co-cultures with HC myotubes, before and after MN stimulation with 10 mM KCl (arrow). **D** Quantification of the relative fluorescence (*F*/*F*_0_) in NT, sh-Scr treated, and sh-SLITRK2 treated FUS-ALS MN before and after MN stimulation. **E** Quantification of *F*max (*F*_max_-*F*_0_) in NT, sh-Scr treated, and sh-SLITRK2 treated FUS-ALS MN co-cultured with HC myotubes. (*p* = 0.0538). **F** Relative fluorescence (*F*/*F*_0_) of Ca^2+^ intensity over time in HC myotubes co-cultured with NT, sh-Scr treated, and sh-SLITRK2 treated FUS-ALS MN, before and after MN stimulation with 10 mM KCl (arrow). **G** Quantification of the relative fluorescence (*F*/*F*_0_) in HC myotubes co-cultured with NT, sh-Scr treated, and sh-SLITRK2 treated FUS-ALS MN. **H** Quantification of *F*max (*F*_max_-*F*_0_) in HC myotubes co-cultured with NT, sh-Scr treated, and sh-SLITRK2 treated FUS-ALS MN. *All data in C-H is presented as mean ± SD (*n* = 3, *t* = 4). D + E + G + H: Kruskal-Wallis test. Source data are provided as a Source Data file.

Based on these results, we next assessed whether reversing the pathological upregulation of SLITRK2 in FUS-ALS MN would restore NMJ function in FUS-ALS co-cultures (Fig. 9A). Sustained knockdown of *SLITRK2* in MN was confirmed at D28 (Fig. 9B). KCl-induced MN depolarization resulted in a significant increase in RF in FUS-ALS MN in both sh-Scr and sh-SLITRK2 conditions (Fig. 9C, D). Analysis of the peak Ca^2+^ response (*F*max) in MN revealed that *F*max remained low in FUS-ALS MN following SLITRK2 KD (Fig. 9E). In contrast, postsynaptic responses in HC myotubes were significantly increased upon SLITRK2 KD in FUS-ALS MN, with increased Ca²⁺ signal intensity and restored *F*max compared to sh-Scr-treated co-cultures (Fig. 9F–H). These functional changes were not accompanied by an obvious increase in NMJ innervation (Supplementary Fig. 13A–C).

Together, these results show that SLITRK2 KD does not reverse hypoexcitability phenotypes in FUS-ALS MN. However, reducing elevated SLITRK2 levels can normalize reduced NMJ signaling at the functional level in FUS-ALS, most likely by improving the efficacy of existing synaptic connections rather than by increasing NMJ innervation.

## Discussion

Accumulating evidence identifies changes in the distal axon and NMJ as early pathogenic events in ALS. The mechanisms underlying these changes likely include defects in local gene expression and translation^6^. Here, we determined compartment-specific 3’UTR isoform expression in HC human MN axons and exploited FUS-ALS to establish the impact of ALS mutations on local gene and isoform expression. Our data provide insight into compartment-specific 3’UTR expression in control and ALS MN, present a fully iPSC-derived iNMJ-on-a-chip model, and link elevated SLITRK2 to functional synaptic defects in ALS.

Human in vitro models that accurately recapitulate NMJ dysfunction in neuromuscular disorders are essential for dissecting pathogenic mechanisms and drug discovery^84^. Here, we developed an iNMJ-on-a-chip model which combines iPSC-derived MN with mature, contractile iPSC-derived myotubes within a compartmentalized microfluidic device. This represents a key advance, as most 2D humanized NMJ-on-a-chip models have thus far relied on alternative myotube sources^36, 51–55^ or iPSC-derived myogenic cells obtained from external sources^85^. Our platform allows the generation of models in which all cell types are iPSC-derived and thus can harbor genetic disease backgrounds of choice. Microfluidic platforms offer precise control of the cellular microenvironment, facilitating studies of NMJ formation, synaptic transmission, and other disease mechanisms, such as axonal transport^74^. Further, its compartmentalized design allows targeted compound testing on either diseased MN or myotubes. This is particularly valuable for assessing cell type-specific effects of candidate drugs, e.g., compounds may improve neuronal function but negatively impact myotubes^86^.

APA changes in human axons or as a result of *FUS* mutations remain unknown. We therefore implemented an axon-seq approach to assess compartment-specific isoform switching in control and diseased MN. Several genes in HC MN displayed compartment-specific 3’ UTR isoforms. For example, *TAFA5*, a brain-specific chemokine, exhibited enrichment of distinct isoforms in axonal versus somatic compartments. This is interesting as isoform differences can critically impact processes such as RNA distribution and stability^8, 13^. Our data further show that ALS-associated FUS mutations induce specific APA changes in MN axons. A compelling example of such a change was the upregulation of an *SNCA* transcript isoform utilizing a more proximal polyadenylation site, resulting in a shorter 3’ UTR. This proximal region contains predicted FUS binding sites, confirmed by crosslinking immunoprecipitation (CLIP) of FUS targets^21, 50, 87^. Mutant FUS specifically binds the 3’ UTR region of *SNCA*^87^, suggesting that *FUS* mutations could directly induce this isoform change. *SNCA* mutations cause PD, and SNCA is a major constituent of Lewy bodies found in PD patients. Interestingly, extended *SNCA* 3’ UTRs have been associated to neurodegenerative processes and increased risk of PD^75, 88^. The observation that SNCA expression is increased in the spinal cord of ALS patients and SOD1-ALS mice supports the hypothesis that SNCA dysregulation may contribute to ALS more generally^89, 90^. Our work indicates that FUS-ALS is associated with axon-specific changes in 3’ UTR isoforms in MN and provides a first unbiased analysis of such changes in ALS.

To increase our understanding of the molecular mechanisms underlying axonal and synaptic changes in ALS, we focused on SLITRK2. SLITRK2 is a member of the SLITRK family of proteins with roles in synapse formation and neurite outgrowth. SLITRK proteins have been extensively characterized in the central nervous system, but their role in MN and peripheral projections is incompletely understood^60–65, 72^. Our study identified increased expression of *SLITRK2* as the most consistent and robust change in FUS-ALS MN and their axons. This observation was experimentally confirmed in the axonal compartment at both the mRNA and protein level. Our analysis of *SLITRK2* isoforms in HC MNs revealed that while the long 3’ UTR isoform (distal polyadenylation site) is the predominant species in axons, both short and long isoforms are expressed and upregulated in FUS-ALS MN axons.

To begin to understand how ALS-associated FUS mutations may affect SLITRK2 expression, we explored different possible mechanisms. Several published FUS CLIP-seq studies were performed but did not report *SLITRK2* as a direct FUS-binding target^21, 44, 91–100^. To confirm this observation, we performed immunoprecipitation (IP) of FUS followed by qPCR. These experiments failed to detect FUS-*SLITRK2* binding (Supplementary Fig. 14A–F). Next, we assessed whether SLITRK2 is deregulated as a consequence of altered microRNA (miRNA) expression known to be induced by FUS mutations. However, none of the miRNAs predicted to bind SLITRK2 were altered in FUS-ALS MN axons (data not shown). Finally, we examined the axonal distribution of FUS and observed pronounced mislocalization of FUS to neurites in FUS-ALS (Supplementary Fig. 14G, H). These observations suggest that FUS mutations may indirectly affect SLITRK2, for example, by changing the localization of upstream gene expression regulators, several of which have been shown to bind FUS and mislocalize in FUS-ALS.

In line with our observation of increased SLITRK2 levels in FUS-ALS, a common variant associated with higher *SLITRK2* expression had been reported to correlate with an earlier age of onset in C9ORF72-ALS patients. This allele was linked to decreased levels of the synaptic vesicle proteins SV2 and SYP in the frontal cortex of these patients, hinting at a link between elevated *SLITRK2* levels and synaptic alterations in ALS^71^. To functionally establish a pathogenic role of increased SLITRK2, we performed (1) OE of SLITRK2 in HC MN to mimick the changes observed in FUS-ALS MN, and (2) KD to normalize SLITRK2 expression in FUS-ALS MN followed by analysis of FUS-ALS-mediated axonal and synaptic phenotypes. First, we exploited a previously reported axon growth phenotype in FUS-ALS MN monocultures. Increased neurite growth was described previously in iPSC-derived FUS-ALS MN cultures^37, 39^ and in cultures using different ALS genetic backgrounds, such as SOD-1-ALS^101–104^. SLITRK2 OE induced increased axon growth in HC MN, while SLITRK2 KD reversed enhanced axonal growth observed in FUS-ALS MN, implicating this transmembrane protein in ALS-associated axonal defects. This effect aligns with the ability of SLITRK2 to promote neurite outgrowth from dopaminergic neurons^61^. In neuronal cell lines, i.e., Neuro2A and PC12, SLITRK2 has been found to inhibit neurite growth^62^, hinting at cell-type specific effects.

As axonal deficits may trigger synaptic changes, we next assessed NMJs using the iNMJ-on-a-chip platform. Previous studies reported reduced NMJ numbers and impaired NMJ maturation in co-cultures of FUS-ALS MN and wild-type mesoangioblast-derived myotubes^34, 36^. Using iPSC-derived FUS-ALS MN and wild-type iPSC-derived skeletal muscle cells, we detected a reduction in the number of innervated NMJs in FUS-ALS co-cultures but no change in the number of AChR clusters. MN-specific OE of SLITRK2 in HC MN-myotube co-cultures did not affect NMJ number or innervation. In line with this, SLITRK2 KD in FUS-ALS MN did not reverse the observed reduced NMJ innervation. While a reduction in the innervation of NMJs appears to contradict the enhanced outgrowth of axons observed in MN mono-cultures, no increase in MN axon growth was observed in FUS-ALS co-cultures. It is possible that the presence of wild-type myotubes normalizes this defect or that this ALS phenotype transitions in time, as has been shown for other defects^104, 105^.

Lowering SLITRK2 levels in FUS-ALS MN significantly reversed the reduction in postsynaptic responses in myotubes, as assessed by live Ca^2+^-imaging. Interestingly, while FUS-ALS MN were hypo-excitable in our cultures, as reported previously for FUS-ALS MN in other models^81–83^, this defect was not reversed by SLITRK2 KD. Similarly, SLITRK2 OE did not affect MN excitability but reduced postsynaptic myotube responses. As outlined above, previous work had reported structural NMJ defects in FUS-ALS co-cultures^34, 36^. Our present study not only confirms but also significantly extends these previous findings by for the first time, showing that FUS mutations can cause functional defects in neurotransmission at the human NMJ.

A plausible explanation for the rescue effects of reducing SLITRK2 on NMJ transmission is that this intervention increases the efficacy of existing synaptic connections. SLITRK2 has previously been shown to modulate synaptic neurotransmission through BDNF/TrB signaling. BDNF-TrB-FL (full length) signaling is essential for neuronal survival and presynaptic function^106–108^. BDNF expressed by contracting skeletal muscle ^139^ modulates neurotransmission, such as activity-induced ACh release^109^. Truncated TrkB isoforms (TrkB-T) competitively inhibit phosphorylation of TrkB-FL upon BDNF binding and suppress this signaling pathway^110, 111^. SLITRK2 overexpression can decrease TrkB-FL and increase truncated TrkB-T levels^65^. It has been shown that, in line with our findings, chronic inhibition of BDNF/TrkB signaling causes neuromuscular transmission failure without inducing morphological changes at the NMJ^112^.

Together, our data and the iNMJ-on-a-chip model provide a valuable framework for further dissecting compartment-specific RNA isoform changes that may drive early dysfunction at the distal axon and NMJ. Given that elevated *SLITRK2* expression has also been genetically linked to C9ORF72-ALS, its therapeutic relevance may extend beyond FUS-ALS. Moreover, dysregulated *SLITRK2* expression has been associated with neuromuscular defects in myotonic dystrophy type 1 (DM1). Although how ALS-associated mutations in FUS cause upregulation of *SLITRK2* mRNA remains incompletely understood, our results identify *SLITRK2* as a promising target for restoring synaptic function in ALS.

## Methods

### iPSCs

#### iPSC generation and characterization

The iPSC lines used in this study, either generated at Utrecht Medical Center, Utrecht or provided by others^113–117^, were reprogrammed from skin fibroblasts. A detailed list of the iPSC lines used is provided in Supplementary Table S1. All experimental procedures involving iPSCs were approved by the Medical Ethical Committee of the University Medical Center Utrecht through biobank protocol 16–436. Donors gave written informed consent. iPSCs were characterized for pluripotency markers by immunocytochemistry using the StemLight Pluripotency Antibody Kit (Cell Signaling, 9656S) and for stem cell markers by quantitative reverse transcription PCR (qRT-PCR). Pluripotency was further assessed by differentiation of iPSCs into the three germ layers using the STEMdiff^TM^ Trilineage Differentiation Kit (Stemcell Technologies, 05230) and stained for markers of the three germ layers. Cells were karyotyped by G-band staining. The *FUS* mutation in the FUS-ALS patient iPSC-line FUS3 was confirmed by genomic DNA isolation with the genomic DNA extraction kit (Qiagen, 69504) and subsequent PCR-amplification using the *FUS* outer primers (FUS_outer_fw; FUS_outer_rv)(Supplementary Table S2). Sanger sequencing was carried out by Macrogen Europe using the *FUS* forward primer (FUS_fw).

#### iPSC culture and maintenance

iPSCs were cultured feeder-free on Geltrex-coated (Thermofisher, A1413202) dishes in StemFlex medium (Thermofisher, A3349401) and maintained at 37 °C with 5% CO_2_. Cells were passaged weekly with 0.5 mM EDTA and cultured in StemFlex medium supplemented with 5 µM ROCK-inhibitor Y-27632 (Axon Biochemicals, 1683). All lines were maintained in culture up to 60 passages and frequently tested for mycoplasma contamination using a Lonza testing kit (Lonza, LT07-318).

### Differentiation of iPSCs into spinal motor neurons

To generate spinal MN, iPSCs were differentiated following an adapted version of a previously established protocol^66^. Briefly, iPSCs were dissociated with 0.5 mM EDTA and split at a ratio of 1:10-1:15 on Geltrex-coated 6-well plates in StemFlex medium. The differentiation into neural epithelial progenitors was initiated 48 hours after passaging by switching to neuronal differentiation medium 1 (NDM1, Supplementary Table S3) for 6 days with medium changes every other day. After 6 days, neural epithelial progenitors were induced into MN progenitors by switching to NDM2 (Supplementary Table S3) for an additional 6 days, with daily medium changes. At D12, MN progenitors were dissociated with Dispase (Stemcell Technologies, 07923) for 5 min at 37 °C and cultured in suspension in 10 cm dishes in NDM3 (Supplementary Table S3) to induce MN differentiation with medium changes every other day. At D18, MN were dissociated with Accutase (Thermofisher, A11105-01) for 30–45 min at 37 °C into single cells and either frozen for cryopreservation or plated on 20 µg/ml Poly-D-lysine (PDL) (Sigma, P0899) and 10 µg/ml Laminin (Thermofisher, 23017-015)-coated plates, coverslips or microfluidic devices (see section: “Generation of iNMJ-on-a-chip cultures”). MN were cultured in NDM4 (Supplementary Table S3) for 9 days to induce maturation with medium changes every 2 to 3 days.

### RNA isolation and RT-qPCR

Total RNA was extracted using the RNeasy mini kit (Qiagen, 217084) following the manufacturer’s protocol, which included on-column DNAse treatment (Qiagen, 79254). RNA concentration and purity were measured in a NanoDrop 2000 Spectrophotometer. For cDNA synthesis, the SuperScript IV Reverse Transcriptase kit (Thermofisher, 18090010) was used. 200 ng RNA was incubated with 1 µl random hexamer primers, 1 µl 10 mM dNTPs and RNAse free H_2_O to a total volume of 20 µl for 5 min at 65 °C followed by 2 min incubation on ice. Next, a mixture containing 4 µl first strand buffer, 1 µl DTT, 1 µl RNAse inhibitor and 1 µl Superscript SSIV Reverse Transcriptase was added to each sample and incubated for 10 min at 23 °C, 10 min at 53 °C and 10 min at 80 °C. For RT-qPCR, 4 ng cDNA was mixed with 2x FastStart Universal SYBR Green Master (Rox; Roche, 4913914001) and 0.5 µl 10 µM of forward and reverse primer, which were generated using the NCBI primer tool (Supplementary Table S2). The reaction was run in a QuantStudio 6 Flex System qPCR cycler (Applied Biosystems) using the standard PCR program. Each sample was run in triplicate, and the 2^-(Δ)ΔCT method was used to calculate relative expression with Ct values normalized to the mean Ct values of two housekeeping genes.

### Microfluidic chamber preparation

Polydimethylsilxane (PDMS) microfluidic chambers were prepared according to a previously published protocol^78^ with the following chamber dimensions: channels: length 750 µm, width 8 µm; grooves: length 300 µm, width 15 µm. Briefly, microfluidic chamber molds were casted with a mixture of silicone elastomer base and curing reagent (10:1) using the SYLGARD^TM^ 184 Silicone Elastomer Kit (Dow, 1673921), and cured at 60 °C overnight. After curing, the chambers were carefully removed from the mold, and 5 mm wells were punched on either side of the channels. The chambers were cleaned with adhesive tape and sterilized with 70% ethanol. Immediately following plasma cleaning, the chambers were attached to 24 mm coverslips using gentle pressure and heated to 60 °C to enhance adhesion to the glass.

### Plating of D18 iPSC-derived motor neurons in microfluidic devices

Prior to seeding the MN, the microfluidic devices were first coated with PDL (20 µg/ml) for 1 hour at 37 °C, followed by 3 wash steps with Milli-Q, and replaced by Laminin (10 µg/ml) for another 1 h at 37 °C. D18 MN were dissociated or thawed (see: “Differentiation of iPSCs into spinal motor neurons”) and seeded at 150.000 cells/chamber in 6 µl/chamber NDM4 in the proximal chamber, while 6 µl/chamber NDM4 was added to the distal chamber to prevent migration of the cells through the microgrooves. Approximately 1 h after seeding, when MN were attached, additional NDM4 medium was added to the wells of the MN chamber to reach maximum volume. The medium was changed every 2 to 3 days.

### Immunocytochemistry on iPSC-derived motor neurons

MN were fixed with 4% paraformaldehyde (PFA) (Thermofisher, 50-980-487) for 20 min at 4 °C. Following fixation, cells were permeabilized with 0.1% Triton-X-100 (Sigma, 10789704001) in PBS for 5 min at RT. Cells were blocked in blocking buffer (5% Normal Donkey Serum, 1% Bovine Serum Albumin, 0.1% Triton-X-100 in PBS) for 30 min at RT. Cells were then incubated overnight at 4 °C with primary antibodies (Supplementary Table S4) in blocking buffer. The next day, cells were washed 3x with 0.1% Tween (Sigma, P1754) in PBS (PBS-T) for 15 min at RT and subsequently incubated with secondary antibodies (Supplementary Table S4) in blocking buffer for 1 hour at RT. Cells were washed 2x with PBS for 10 min at RT, followed by a 10-minute incubation with DAPI at RT to stain for nuclei. A final wash was performed twice with PBS for 10 min at RT, and after the last wash, cells were mounted with FluorSave (Sigma, 345789). Imaging was performed using a Zeiss LSM 880 Confocal Microscope equipped with Zen 3.3 image acquisition software (Zeiss) or using a Thunder Imaging System (Leica).

### RNA isolation, library preparation by CEL-Seq2, and sequencing

MN somas and axons were harvested at D27 of the MN differentiation protocol, following 9 days of culture in the microfluidic devices. RNA was isolated separately from the axonal and/or somatodendritic compartments of 5 microfluidic devices. To collect axonal RNA, 30 µl of lysis buffer, consisting of 2% Triton X-100 (in MilliQ) supplemented with 1.5 µ/µl of RNAse inhibitor, was flushed through the distal compartment of each device, pooled together and immediately snap-frozen on dry ice. Somatodendritic RNA was then collected from the proximal compartment using the same procedure. The lysates were concentrated from 150 µl to 75–90 µl using a centrifugal concentrator. RNA was purified using AMPure XP Beads and directly eluted in 1.1 µl CEL-seq2 primer 1 to initiate RNA amplification using the CEL-seq2 method^59^. Briefly, RNA was subjected to reverse transcription, 2^nd^ strand synthesis, in vitro transcription, and fragmentation. Fragmented RNA was purified using AMPure XP Beads, and RNA concentration and quality were assessed using an Agilent Bioanalyzer. Samples were stored at − 80 °C until further use. Library preparation was initiated with 5 µg of amplified, fragmented RNA. RNA was reverse transcribed, and PCR amplified for 8 (somatodendritic samples) or 12 cycles (axonal samples) using a uniquely indexed RNA PCR primer for library labeling. The resulting cDNA was purified using AMPure XP Beads, and cDNA quality and fragment size were assessed using an Agilent Bioanalyzer. cDNA concentration was quantified using the Qubit dsDNA High Sensitivity Assay. cDNA libraries were pooled at a concentration of 1 ng/µl and subjected to paired-end sequencing on an Illumina Nextseq 2000 (somatodendritic versus axonal samples; 2 x 50 bp), a NovaSeq X Plus Series (somatodendric versus axonal samples, 2 × 150 bp) or an Illumina Novaseq 6000 platform (FUS-ALS versus HC/FUS-iso axonal samples; 2 x 150 bp), generating approximately 20–30 M paired-end reads per library.

### RNA-seq data read alignment and gene count matrix generation

Read alignment and count matrix generation were performed on the local high-performance computing cluster. Cell barcode and UMI detection were performed using UMI-tools^118^. Reads were trimmed using Cutadapt to remove low-quality reads and adapter sequences^119^. The settings were *--nextseq-trim* = *15* and *-minimum-length 50* for quality trimming and *-a “GATCGTCGGACTGTAGAACTCTGAAC;min_overlap* = *13”* to remove the Illumina adapter. Fastq files were aligned to the reference genome (GRCh38) using STAR^120^. Deduplication of aligned bam files was performed using UMI-tools. Feature count matrices were generated using Subread’s command-line featureCounts function and Ensembl’s gene annotation version 98^121^. Genes with low counts (minimum of 5 reads in at least 3 samples) were removed from the dataset.

### RNA-seq data differential expression analysis

Normalization of counts and differential expression analysis was performed using the DESeq2 package (v1.42.0) in RStudio (v4.3.1). For the somatodendritic versus axonal compartment analysis, the DESeq2 model included “~Compartment” to account for differences between axonal and somatodendritic samples. Exploratory data analysis, including principle component analysis (PCA) and heatmap generation, was conducted on the regularized log transformation (rld) counts data. Differentially expressed genes (DEGs) were defined as those with a |Log2 fold change| ( | LFC | ) > 1 (FC > 2) and adjusted *p*-value  < 0.05. Genes were filtered for a baseMean > 10. Functional interpretation of DEGs was conducted using the Gene Ontology (GO) databases and the ClusterProfiler package (4.10.0) in R. GO term enrichment analysis for biological processes was performed using the *EnrichGO* function with an adjusted *p*-value < 0.05 (Benjamini-Hochberg correction).

For analysis of FUS-ALS vs HC/FUS-iso axonal samples, raw counts were first normalized using the DESeq2 package, incorporating “~Batch + Condition” in the model. Samples with < 1.000.000 mapped reads were excluded from the analysis. Variance-stabilizing transformation (vst) was applied to the counts data for exploratory data analysis, PCA and heatmap generation. To ensure axonal samples were not cross-contaminated with cell soma material, several somatodendritic and axonal markers were validated in the axonal samples. Samples containing low expression of axonal markers were excluded from the analysis. Subsequently, differential expression analysis was performed using DESeq2, with principal components 1 and 2 (PC1 and PC2) included in the design formula ( ~ Batch + PC1 + PC2 + Condition) to account for potential batch effects and variance captured by PCA. Genes were filtered for a baseMean > 10. DEGs were defined as those with |LFC | > 0.58 (FC > 1.5) and *p*-value < 0.05. To determine statistically overrepresented genes, gene set enrichment analysis (GSEA) was performed using GSEA software v4.3.2^122, 123^ and the C5.go.v2024.1.Hs.symbols.gmt gene set (filter min = 15, max = 200) from MSigDB^124^.

### RNA-seq data peak calling for 3’ UTR isoform identification

To identify APA sites, peak calling was performed on the RNA-seq data for HC somatodendritic vs axonal samples and the RNA-seq data for FUS-ALS vs HC/FUS-iso axonal samples. Read alignment, trimming, and deduplication were performed as described previously. Reads were then filtered for putative internal priming artefacts using polyAfilter with the settings *COVLEN* = *300* and *MINSNRLEN* = *10*, allowing for one mismatch. The filtering was performed considering both exons and introns^125^. Aligned read files for each sample were merged into a single BAM file. Peak calling was performed using the *FindPeaks* function from the Sierra R package^68^. Default settings were used, with the exception of *min.cov.cutoff* = *400* and *min.peak.cutoff* = *200*. Ensembl version GRCh38.98 was used as the reference list. To ensure accurate detection of peaks located at the terminal ends of transcripts, the 3’ UTR annotation was extended by 500 bp downstream. Reads covering peaks were quantified using featureCounts. To specifically focus on APA sites within genes, subsequent differential expression analysis considered only genes exhibiting more than one distinct peak. Differential expression analysis of the identified peaks was performed using the DESeq2 package in R, incorporating “~Line + Compartment” (HC axonal versus somatodendritic) or “~Batch + Condition” (FUS-ALS versus HC/FUS-iso axonal) in the model to correct for technical batch variation. DEGs were defined as those with a |Log2 fold change| ( | LFC | ) > 1 (FC > 2) and *p*-value  < 0.05. Genes were filtered for a baseMean > 10.

### RNAscope in situ hybridization

RNAscope was performed using the RNAscope Multiplex Fluorescent Reagent Kit v2 (ACD Bio-Techne, 323100) according to the manufacturer’s protocol for cultured adherent cells, with an adjusted Protease III dilution of 1:20. The coding region of *SLITRK2* was targeted using the commercially available Hs-SLITRK2-C1 probe (ACD Bio-Techne, 1567271-C1). This was combined with a custom-designed probe (Hs-SLITRK2-O1-C2, ACD Bio-Techne) targeting nucleotides 5472-7208 of the *SLITRK2* transcript (NM_032539.5) to specifically detect the long 3’ UTR. Signal detection was performed using TSA Vivid Fluorophores 570 (ACD Bio-Techne, 323272) and 650 (ACD Bio-Techne, 323273). Following RNAscope, immunocytochemistry was performed as described above. Z-stack images were acquired using a Zeiss LSM 880 confocal microscope equipped with an EC Plan-Neofluar 40x/1.30 Oil DIC objective (Zeiss, 420462-9900-000). Probe quantification was performed on blinded images by manually counting probes through the z-stack using ImageJ software (Fiji version 2.14.0/1.54 f).

### Mouse strains

All animal experimental procedures were conducted according to the KU Leuven ethical guidelines and approved by the KU Leuven Ethical Committee for Animal Experimentation (ECD number: Neuro-021/2025). Mice (C57BL/6) were maintained in standard housing conditions, with a temperature of 22 ± 2 °C and humidity of 40–60%, on a diurnal 12 h light/12 h dark cycle and with continuous access to food and water.

### Immunohistochemisty on mouse sciatic nerve tissue

12 months old male mice were perfused intracardially and fixed with 4% PFA in 0.1 M phosphate buffer (pH 7.2). Sciatic nerves were dissected, postfixed for 3 h in the same fixative and transferred to a 30% sucrose phosphate buffer for at least 2 days. Proximal sciatic nerve regions were embedded in optimal cutting temperature compound (Sakura) and snap-frozen in isopentane (2-methylbutane) cooled at − 40 °C on dry ice. Mounted sciatic nerve sections (10 μm) were incubated in blocking solution 1.5% Bovine Serum Albumin, 0.5% Tween-20 in PBS) for 1 h at RT. RT. Sections were then incubated for 48 h at 4 °C with primary antibodies against SLITRK2 (Supplementary Table S4, 1:100) and Neurofilament H (NF-H, Supplementary Table S4, 1:500) diluted in 0.3% Triton-X-100 in PBS. Following primary antibody incubation, sections were washed with PBS and subsequently incubated with secondary antibodies (donkey anti-rabbit Alexa-647, donkey anti-chicken Cy3, 1:500) for 1 h at RT. Sections were washed with PBS, followed by a 10 min incubation with DAPI at RT to stain for nuclei. A final wash was performed with PBS, and sections were mounted with ProLong Gold mounting medium (Invitrogen). Imaging was performed using a Nikon Ti2 Cicero or Nikon C2 laser scanning confocal microscope, and images were processed using ImageJ software (NIH).

### SH-SY5Y cell culture and differentiation

SH-SY5Y cells (ATCC® CRL-2266™; RRID:CVCL_0019) were cultured in DMEM High Glucose (Thermo Fisher, 11-965-092) supplemented with 10,000 U/ml Penicillin-Streptomycin (Thermo Fisher, 15140122), 200 mM L-glutamine (Life Technologies, 25030024), and Fetal Bovine Serum (TICO EUROPE, FBS EU500). Cells were maintained at 37 °C with 5% CO2 and passaged weekly using Trypsin at a 1:10 ratio. To differentiate SH-SY5Y cells into post-mitotic, mature neuron-like cells, the medium was switched to a differentiation medium (Supplementary Table S5) based on a modified protocol from Chiocchetti et al. ^126^. Cells were differentiated for 11 days, with the medium refreshed three times a week

### Immunoprecipitation

Motor neurons or SH-SY5Y were harvested in IP lysis buffer (Supplementary Table S6), passed through a pre-chilled 25 G needle (BD Biosciences, 303175) to ensure complete lysis, and incubated for 10 min on ice. Lysates were centrifuged at 16000 x *g* for 15 min at 4 °C. The resulting supernatant was divided into two equal aliquots, after reserving 20 μl fractions for both RNA and protein input analysis. Aliquots were incubated overnight at 4 °C with either 0.6 μg/μl rabbit anti-FUS (Proteintech, 11570-1-AP) or 1.0 μg/μl rabbit anti-IgG (R&D Systems, AB-105-C) serving as a negative control. Concurrently, 30 μl of Protein G Dynabeads (Invitrogen, 10003D) were blocked in lysis buffer (without fresh additives) supplemented with 3% Bovine Serum Albumin for 1 h. Following magnetic separation to remove the blocking buffer, the antibody-lysate mixtures were added to the beads and incubated for 3 h at 4 °C. Beads were subsequently washed five times with complete lysis buffer at 4 °C. After the final wash, the bead suspension was partitioned: 100 μl was collected in sample buffer for Western blot analysis, and 400 μl was collected in QIAzol lysis reagent for subsequent RNA isolation and RT-qPCR.

### Western blot analysis

For Western blot analysis, samples were mixed with 1x NuPAGE loading buffer (Invitrogen, NP0007) and β-mercaptoethanol and denatured by heating for 5 min at 95 °C. For each sample, 30 μg of protein was loaded alongside a 10–250 kDa PageRuler Plus Prestained Protein Ladder (Thermo Fisher, 26620) onto a 10% SDS-PAGE gel. Proteins were separated by gel electrophoresis for 15 min at 80 V, followed by 1 h at 150 V. Following separation, proteins were transferred onto a 0.45 μm pore size Nitrocellulose C membrane (Cytiva, 10600002) using a Wet/Tank Transfer Blotting System (Bio-Rad, 170390) for 1 h at 100 V. Total protein staining was performed using the Revert Total Protein Stain kit (LI-COR Biosciences, D36302-D2) according to the manufacturer’s protocol. Membranes were blocked in blocking buffer (5% ELK in TBS containing 0.1% Tween-20 (TBS-T)) for 1 h at RT, and subsequently incubated overnight at 4 °C with rabbit anti-FUS (Proteintech, 11570-1-AP) primary antibody diluted in blocking buffer. The following day, blots were incubated with horseradish peroxidase (HRP)-conjugated secondary antibodies (GAR-PO: Jackson Immunoresearch, 111-035-003; 1:50.000) in blocking buffer for 1 h at RT. Membranes were then washed three times in TBS-T and once in Milli-Q water. Blots were incubated in SuperSignalTM West Dura Extended Duration Substrate (Thermo Scientific, 34076) for 5 min and imaged using the UVITEC Cambridge Western blot imaging system (BioSPX).

### Axonal outgrowth analysis

Axonal outgrowth was analyzed as previously described^36^. Briefly, 4 days after plating MN in microfluidic devices, cells were fixed and stained for TUBB3 as described in the immunocytochemistry section. Tile scan images of TUBB3 were taken at 10x magnification of the axonal compartment, using a Thunder Imaging System (Leica) equipped with a HC PL FLUOTAR 10x/0.32 PH1 objective. MN axons were isolated using Ilastik 1.4.0 Pixel Classification software^127^. To quantify neurite outgrowth, a linear Scholl analysis script^36^ was used to calculate the number of pixel intersections per intersection line every 50 µm using ImageJ v1.53t software.

### Differentiation of iPSCs into skeletal muscle

To generate skeletal muscle, iPSCs were differentiated following an adapted version of a previously published protocol^56^. Briefly, iPSCs were dissociated with 0.5 mM EDTA (Thermofisher, 15-575-020) and split at a ratio of 1:12 on Geltrex-coated 6-well plates in StemFlex medium. The differentiation into myogenic progenitors was initiated 24 hours after passaging by switching to myogenic differentiation medium (Supplementary Table S7) supplemented with 8 µm CHIR99021 (Sigma, SML1046) for 2 days. Following this, CHIR99021 was removed, and myogenic differentiation medium was supplemented with 20 ng/ml FGF2 (PeproTech, 100-18B) for 14 days. For an additional 14 days, cells were then cultured in myogenic differentiation medium only with daily medium changes. At D30, C-MET-positive (1:50, R&D Systems, FAB3582A) and HNK-1-negative (1:100, eBioscience, 12-0577-42) myogenic progenitors were purified by FACS and cultured in myogenic proliferation medium (Supplementary Table S7) supplemented with 100 ng/nl FGF2 on plates coated with extracellular matrix (ECM) (Sigma, E1270) diluted 1:200 in myogenic proliferation medium. To reduce cell death, the medium was supplemented with 1x RevitaCell Supplement (Gibco, A26455-01) during cell collection and the first 24 h of culture after FACS. Myogenic progenitors were expanded with daily medium refreshment. Upon reaching 90% confluency, cells were either frozen for cryopreservation or passaged using 2x diluted TrypLE (Gibco, 12604-021) in PBS. To initiate differentiation into multinucleated myotubes, myogenic progenitors were cultured until they reaching 90% confluency, at which point myogenic proliferation medium was switched to myogenic maturation medium (Supplementary Table S7) supplemented with 10 µM DAPT (Sigma, D5942), 10 µM Forskolin (Cayman Chemical Company, 11018-50), 10 µM Dexamethasone (Cayman Chemical Company, 11015-1) and 10 µM SB43 (Axon Biochemicals, 1661) to enhance maturation efficiency^58^ for a duration of 12 days, with medium changes every 2 to 3 days.

### Generation of iNMJ-on-a-chip cultures

For the generation of iNMJ-on-a-chip cultures, microfluidic chambers were prepared as described above. Myogenic progenitors were either dissociated when reaching 90% confluence or thawed and seeded (see “Differentiation of iPSCs into skeletal muscle”) in the myotube chamber, coated with ECM (1:200). Myogenic progenitors were seeded at 100.000 cells/chamber in 6 µl/chamber myogenic proliferation medium supplemented with 100 ng/ml FGF2, while a minimum of 6 µl PBS was added to the MN chamber to avoid migration of the cells through the microgrooves. Approximately 2 h after seeding, when myogenic progenitors were attached, wells of the myotube chamber were filled up with myogenic proliferation medium supplemented with 100 ng/ml FGF2. 24 h after seeding, at D0, the formation of multinucleated myotubes was initiated by switching to myogenic differentiation medium supplemented with 10 µM DAPT, 10 µM Forskolin, 10 µM Dexamethasone and 10 µM SB43. At D3, myogenic progenitors that migrated towards the MN chamber were removed. After washing the MN chamber with PBS, migrated cells were dissociated with Trypsin-EDTA (0.05%; Thermofisher, 25300062) for 15 min at 37 °C and removed by washing with PBS. Next, the MN chamber was coated with PDL (20 µg/ml) for 1 h at 37 °C, followed by 3 wash steps with Milli-Q, and replaced by Laminin (10 µg/ml) for another 1 h at 37 °C. MN at D18 were dissociated or thawed (see “Differentiation of iPSCs into spinal motor neurons”) and seeded at 150.000 cells/chamber in 6 µl/chamber NDM4 in the MN chamber. Approximately 1 h after seeding, when MN were attached, additional NDM4 medium was added to the wells of the MN chamber to reach maximum volume. The medium was changed every 2 to 3 days.

### Calcium fluorescent imaging

#### Live cell calcium imaging

To measure MN and myotube activity, live cell calcium imaging was conducted at D30 of MN differentiation and D10 of myotube maturation. MN and myotubes were incubated with 5 µM Cal-520 (Bio-Connect, AB171868) in respectively artificial cerebral spinal fluid (aCSF) or myogenic maturation medium for 60 min at 37 °C. aCSF consisted of 124 mM NaCl, 2.5 mM KCl, 10 mM HEPES, 11 mM Glucose, 26 mM NaHCO_3_, 1 mM NaH_2_PO_4_, 1.3 mM MgCl_2_ and 2.5 mM CaCl_2_·2H_2_O. Cal-520 fluorescence was recorded using a Thunder Imaging System (Leica) with a time interval of 235 ms/frame, at 10x magnification using the HC PL FLUOTAR 10x/0.32 PH1 objective. MN were stimulated with 10 mM KCl in aCSF without MgCl_2_ to prevent NMDA receptor blockade^128^. Fluorescence activity was measured 60 sec before and 90 sec after stimulation. In addition, myotubes were treated with 25 μM of the acetylcholine receptor (AChR) competitive antagonist tubocurarine hydrochloride pentahydrate (DTC) (Sigma, T2379) for 10 min in myogenic maturation medium. Fluorescence activity was measured again 60 sec before and 90 sec after MN stimulation with 10 mM KCl in aCSF without MgCl_2_.

#### Data analysis

Data analysis was performed using ImageJ v1.53t software. Prior to analysis, the background subtraction function was applied to all captured images to reduce background noise. For MN, 3 random regions of interest (ROIs) containing MN clusters were measured per condition. For myotubes, 3 ROIs were set around MN-responsive myotube bundles to specifically assess NMJ innervation. Within each ROI, the raw integrated density was subtracted from the integrated density for every frame to calculate the fluorescent signal per frame (*F*). To determine *relative fluorescence* (*F*/*F*_0_), F was divided by the baseline fluorescence intensity (*F*_0_) for each frame. F_0_ was determined by averaging F over the first 30 s of frames before MN stimulation. *F/F*_0_ was analyzed for 60 s of frames before and after stimulation. Further analysis parameters included *maximum peak value* (*F*_MAX_-*F*_0_)Δ*F*/*F*_0_*F*/*F*_0_*F*/*F*_0_*F*_MAX_. In addition, the frequency of fluorescence peaks was calculated for myotube bundles, as a measurement for *contraction frequency*, using the Find_peaks plugin and subsequently the Find_fluopeaks plugin in ImageJ. The threshold was determined per batch, resulting in values ranging from 400 to 2200. Peaks were manually counted 60 s before and 60 s after stimulation of MN and divided by the total time to calculate *contraction frequency*. *Delta frequency* was defined as the increase in frequency after MN stimulation, calculated as the frequency after stimulation minus the frequency before stimulation.

### Immunocytochemistry on iNMJ-on-a-chip cultures

To perform immunocytochemistry on iNMJ-on-a-chip cultures, cells were fixed at D12 after the initiation into multinucleated myotubes with 4% PFA for 20 min at 4 °C. Cells were permeabilized with 0.1% Triton-X-100 in PBS for 5 min at RT and blocked in 3% Bovine Serum Albumin (BSA) in PBT for 30 min at RT. For visualization of AChRs on myotubes, a pre-incubation step with α-bungarotoxin (BTX) conjugate (Thermofisher, B35450) in 3% BSA in PBT for 1 h at RT was included. After an additional 3 washes with PBS for 5 min at RT, cells were incubated overnight at 4 °C with primary antibodies (Supplementary Table S4) in 0.1% BSA in PBT. The next day, cells were washed 3x with PBT for 15 min at RT and then incubated with secondary antibodies (Supplementary Table S4) in 0.1% BSA in PBT for 1 hour at RT. Cells were washed 2x with PBS for 10 min at RT, followed by incubation with DAPI for 10 min at RT to stain for nuclei, and again washed 2x with PBS for 10 min at RT. After the last wash, cells were mounted with FluorSave (Sigma, 345789). Imaging was performed on an Olympos IX81S1F-ZDC2 Confocal Microscope equipped with Olympus Fluoview Version 4.1 imaging software.

### NMJ quantification

NMJ innervation was analyzed 10 days after co-culture. Cultures were fixed and stained for α-bungarotoxin (BTX), βIII-tubulin (TUBB3)/synaptophysin (SYP) and MF20 as described in the immunocytochemistry section. Z-stack tile scan confocal images were taken at 20x magnification of the myotube compartment, directly where the microchannels end, using an Olympos IX81S1F-ZDC2 Confocal Microscope equipped with an UPLSAPO 20x objective and Olympus Fluoview Ver. 4.1 imaging software. NMJs were identified through co-localization of TUBB3/SYP and BTX by counting the number of colocalizations manually through each z-stack blindly. The number of innervated myotubes was normalized to the sum of the total % area of MN and skeletal muscle, with a threshold set on 1188 for TUBB3/SYP and 450 for MF20.

### Plasmid cloning

To specifically knock down *SLITRK2*, we generated a short-hairpin (sh)RNA targeting *SLITRK2*, using pLKO.1 as a backbone (#10878, Addgene). The pLKO.1 vector was digested with the restriction enzymes EcoRI (NEB, R3101S) and AgeI (NEB, 3552S) in Cut Smart buffer (NEB, B7204), and the digested backbone was gel-purified from a 0.8% agarose gel. The annealed oligonucleotides (Supplementary Table S8) were then ligated into the digested vector using T4 Polynucleotide Kinase (NEB, M0201), T4 DNA ligase (Roche, 10799009001), 10 mM ATP, and T4 DNA ligase buffer (Roche). The ligated vector was transformed into competent Stbl3 cells (ThermoFisher, C737303), and colonies were selected on ampicillin plates. Positive clones were validated by Sanger sequencing using a forward primer (GGGCCTATTTCCCATGATTCC) to confirm the correct insertion of the shRNA oligonucleotide sequence.

### Lentivirus production

HEK293T cells (ATCC® CRL-3216™; RRID:CVCL_0063) were seeded into two 15 cm culture dishes and transfected using polyethylenimine (PEI) with 96 µg of the pLKO.1 shRNA construct along with third-generation packaging plasmids (19.2 µg pRSV-rev, 28.16 µg pMD2g-Env and 38.40 µg pMDL9-RRE)(kindly provided by Prof. Dr. Didier Trono). Lentivirus was harvested from the culture media 48 h after transfection by ultracentrifugation. The isolated virus was stored at − 70 °C in 1x PBS containing 0.5% BSA. Lentivirus titer was determined by quantifying GFP-positive HEK293T cells 72 h post-infection, with a dilution series of the viral stock. The titer (TU/mL) was calculated using the following formula: titer (TU/mL) = (average number of GFP-positive cells across 5 fields of view * dilution factor * 60 (field-to-well area correction factor).

### SLITRK2 knockdown and overexpression in iPSC-derived motor neurons

For knockdown (KD) and overexpression (OE) experiments, iPSC-derived MN were seeded at 200.000 cells/chamber at D18 of differentiation into PDL- and Laminin-coated microfluidic devices, as described in “Plating of iPSC-derived motor neurons D18 in microfluidic devices” section. The next day, MN were transduced (MOI1) with lentivirus carrying the pLKO.1-shSLITRK2 construct for KD or lentivirus carrying the pLV[Exp]-EGFP/Puro-EF1A > hSLITRK2 construct (Vectorbuilder, VB260120-1473eew) for OE in NDM4. Negative control (NC) conditions were established using either a scrambled shRNA construct or lentivirus carrying the pLV[Exp]-EGFP/Puro-EF1A > ORF_Stuffer construct (Vectorbuilder, VB010000-9389rbj). 24 h post-infection, a full medium change was performed. KD and OE of *SLITRK2* was confirmed by RT-qPCR, following the protocol described in the “RNA isolation and RT-qPCR” section and using the primers listed in Supplementary Table S2. MN samples for RNA collection were harvested at 72 h and 9 days post-infection, corresponding to the time points at which the axonal outgrowth assay and calcium imaging were performed, respectively.

For microfluidic cultures used in the axonal outgrowth assay, transduced MN were fixed for 20 min in 4% PFA at D22 of differentiation and further processed as described in the “Axonal outgrowth analysis” section. For cultures used in calcium imaging experiments, myogenic progenitors were seeded in the myotube chamber of the microfluidic devices one day after MN infection at a density of 100.000 cells/chamber. Cells were plated in myogenic proliferation medium (Supplementary Table S7) supplemented with 100 ng/ml FGF2. The next day, myogenic proliferation medium was replaced by myogenic maturation medium (Supplementary Table S7) supplemented with 10 µM DAPT (Sigma, D5942), 10 µM Forskolin (Cayman Chemical Company, 11018-50), 10 µM Dexamethasone (Cayman Chemical Company, 11015-1), 10 µM SB43 (Axon Biochemicals, 1661) to enhance maturation, and 5 µM araC (Sigma, C1768) to inhibit further proliferation. The medium was changed every 2 to 3 days. Calcium imaging was performed at D30 of MN differentiation and D10 of myogenic maturation as described in the “Calcium fluorescent imaging” section.

### Statistic and reproducibility

All statistical analyses were performed using GraphPad Prism software version 9.5.1 or version 10. Data was tested for normal Gaussian distribution using the Kolmogorov-Smirnov normality test. Outliers were identified using the ROUT method with Q = 0.1-1%. For all normally distributed measurements, (Un)Paired T-tests, One-Way ANOVAs or Two-Way ANOVAs with Tukey’s test for multiple comparisons were used to determineF significance depending on the number of conditions. For non-normally distributed measurements, respectively, a Mann-Whitney test or Kruskal-Wallis test with Dunn’s test for multiple comparisons was used to determine significance. The significance level was set to *p* < 0.05 (two-sided). Results are presented as mean ± SD. Full details on statistical analysis can be found in Supplementary Data 5. All immunofluorescence experiments were repeated in at least three independent biological replicates unless indicated otherwise, and representative images were presented in the manuscript. All NMJ and RNAscope quantifications were conducted blinded to genotype and experimental condition. The remaining analyses were performed without blinding, given the lack of technical feasibility or were evaluated using (semi-)automated analysis.

### Reporting summary

Further information on research design is available in the Nature Portfolio Reporting Summary linked to this article.

## Supplementary information

supplementary Information file

Description of Additional Supplementary File

Supplementary Data 1

Supplementary Data 2

Supplementary Data 3

Supplementary Data 4

Supplementary Data 5

Video S1

Reporting summary

Transparent Peer Review file

## Source data

source data

## Data Availability

All data supporting the findings of this study are provided within the paper and its supplementary information. Raw and processed RNA-seq data generated in this study have been deposited in the NCBI Gene Expression Omnibus (GEO) under accession code GSE308345 [1008 https://www.ncbi.nlm.nih.gov/geo/query/acc.cgi?acc=GSE308345] and are publicly available. A source data file is provided with this paper. All additional information will be made available upon request to the authors. Source data are provided in this paper.
