# Supplementary material for: SLITRK2 knockdown rescues axonal and functional neuromuscular junction deficits in human iPSC-derived mutant FUS-ALS motor neurons
Source: Nat Commun. 2026 Oct 15;17:11662. doi: 10.1038/s41467-026-78419-7 (PMC13759529; doi:10.1038/s41467-026-78419-7)
Supplement: Supplementary file 1 [file 41467_2026_78419_MOESM10_ESM.pdf]

nature portfolio

Peer Review File

SLITRK2 knockdown rescues axonal and functional neuromuscular junction deficits in human iPSC-derived mutant FUS-ALS motor neurons

Corresponding Author: Professor R. Jeroen Pasterkamp

This file contains all reviewer reports in order by version, followed by all author rebuttals in order by version.

Version 0:

Reviewer comments:

Reviewer #1

(Remarks to the Author)
Synapse impairment at an early stage in ALS has been very much in the spotlight in the last few years. The molecular mechanisms that drive this impairment are not well understood, in part due to the current experimental limitations regarding interrogation of the synapse.

This paper uses a clever technique to circumvent that difficulty, by segregating axo-synaptic terminals from a cell body/dendritic neuronal compartment in microfluidic devices.

The authors employ a 3'UTR RNA sequencing approach to simultaneously identify upregulated mRNA expression and differentially expression of 3'UTR isoforms. Comparison of axonal RNA from FUS-ALS model MNs initially with healthy controls, and then with isogenic controls, identified SLITRK2 as a consistently upregulated gene in FUS-ALS axons. SLITRK2 knockdown was found to rescue axonal deficits in FUS-ALS motor neurons. Additionally, by using a novel and innovative co-culture NMJ model, the authors identify that SLITRK2 knockdown can rescue NMJ transmission. The authors do not describe a molecular mechanism to describe the role of SLITRK2 in this rescue.

This manuscript is clever, extremely interesting and very well written. I think this is an excellent addition to the knowledge in the field, and will be of interest to a large body of neurodegenerative disease researchers as well as those interested in axo-synaptic physiology.

However there are a few points that I feel need to be addressed before this manuscript is suitable for publication, one of which is quite a big revision. If the team can address this point then I think this is very suitable for publication.

The major point that needs to be addressed is the elephant in the axon-chamber; the protein of interest, SLITRK2, in the axo synaptic chamber (a chamber which can only contain presynaptic terminals) is “a post synaptic protein” (line 234). As it stands in the paper this cannot be accurate. The sentence needs to be significantly reworded. However as this will be challenging previous understanding of the biology of this protein, it needs to be conclusively shown that the mRNA is localised in the axo-synaptic compartment, and ideally shown visually using specific probes that the 3'UTR isoforms are located in different axonal locations (proximal versus distal). The evidence in the manuscript is only presented at the protein level in axons. There are several ways the researchers could show this- most conclusive would be FISH for mRNA coupled with IF for an axon marker, or a presynaptic marker. Further proof would be to combine FISH for the mRNA combined with IF for a postsynaptic marker, showing conclusively that postsynaptic staining is present in the cell body chamber and not in the axo-synaptic chamber.

Minor points:

Figure 1C and line 130 “MAP2, a dendritic marker, was only detected in the proximal compartment.”

In Figure 1C, merged image. The ISL1 and MAP2 representative colours are too close in colour to be distinguished. This makes it hard to verify that MAP2 is only present in the proximal chamber. Additionally, the bottom inlay needs to be moved further down- I can't tell if those are red or magenta puncta at the bottom of the image (I can't tell if there are MAP2 positive puncta visible in the grooves between compartments). Alternatively, an image of just MAP2 staining from the axo-synaptic compartment would prove this statement.

Figure 1D; this schematic is not accurate. It's clear in the representative image from 1C that there are multiple axons in the cell body compartment (TUBB3 positive, MAP2 negative). Indeed, the authors do acknowledge this in the text (lines 165 and 166), and it is a known and accepted feature of using these devices. However, this should be represented in the schematic.

Figure 3A The schematic for Fig 3A is very helpful. It would be even more helpful if the nomenclature used for the rest of this figure was applied here (for example HC1, FUS-iso 1, etc).

Figure 4 H. This figure is not mentioned in the manuscript text. I think this is due to a typo on line 281 (which should read Figure 4 G-H?). SLITRK2 counts are at 0 in 4 of 6 samples for the axon, and in 2 of 6 samples for the soma. I'm not sure this evidence is enough to say that "differential localization may suggest compartment-specific functions of the two SLITRK2 isoforms in MN".

Fig 5 F and G. What happens when SLITRK2 is knocked down in HC cells?

Reviewer #2

(Remarks to the Author)
Jongh and Wiersema etc al., investigated compartment-specific changes in RNA expression and regulation, identifying altered 3' UTR isoforms in FUS- ALS, including in genes SNCA and SLITRK2. They found SLITRK2 expression was upregulated in axonal compartment of FUS MNs, and its knockdown normalized axonal defects. Using a new neuromuscular microfluidics protocol, they showed SLITRK2 knockdown rescued neuromuscular junction (NMJ) signalling without affecting innervation or MN excitability.

Following comments should be addressed.

1) Mechanistic insight into how FUS mutations affect expression of axonal SLITRK2; is it mediated via transcriptional regulation and / or transport of mRNA? Given this is the novel finding of this study, this should be further investigated.

2) Figure 3J -L : Independent validation of SLITRK2 at mRNA and protein expression, which are crucial for downstream experiments. (3K) Are these expressed in fold change? If so, the changes appear to be modest, although numerically significant. Additionally, there is a confusion regarding whether 2^-dCt (as mentioned in Figure) or 2^-ddCt (as mentioned in Methods) is being used. Figure 3K-L: Could you please confirm if the authors have performed validation of SLITRK2 immunostaining? The representative images provided are difficult to interpret. Additionally, could you please provide information on the number of images and cells analyzed per biological replicate (legend)? Furthermore, it is intriguing that FUS2 iPS MN, which exhibited the least amount (or no substantial change) of SLITRK2 at the mRNA level, showed the highest level of change at the protein level. Authors should comment on this. Does the SLITRK2 alteration affect only the axonal compartment? The authors should investigate the expression (mRNA and protein) of SLITRK2 in whole cell lysate.

3) Figure 4: SLITRK2 isoform usage. It is intriguing that the test compartmentalized usage of the isoform, however, the data presented suggested that in some of the samples, it is below the detection limit, making it challenging to interpret. Could the authors enhance the robustness of their findings by including additional samples and confirming the results via alternative methods, such as qPCR?

4) Figure 7/8 - Given that this is a novel NMJ model developed, it is imperative to characterize the innervated AChR clusters that are co-localized with SYN and BTX. In this study, ICC was performed with a combined antibody of TUB and SYN, which makes it challenging to precisely measure the percentage of innervated AChR clusters. Did SLITRK2 kd alter the number of innervated AchRs?

Reviewer #3

(Remarks to the Author)
In the present manuscript, de Jongh and colleagues use in vitro patient-derived motor neurons to investigate compartment-specific changes in RNA expression. Here, they focus primarily on the ALS-triggering FUS mutation, known to cause changes in axonal transport and synaptic transmission. The authors use microfluidic devices combined with CEL-seq2 to analyse the transcriptome expression from either somato-dendritic or axonal compartments. Moreover, the CEL-seq2 approach allowed them to investigate alternative splicing in both compartments. DEG analysis revealed several differentially expressed genes between the two cell compartments (which were also compared with previously published datasets obtained with mouse cells). Comparisons between multiple control and FUS MNs revealed a consistent upregulation of the SLITRK2 gene in ALS MNs. The effects of SLITRK2 downregulation were then assessed in MN cultures alone, as well as in a fully humanised MN-NMJ culture using both morphological and Ca2+ functional analysis.
The majority of the experiments performed in this study is elegant, of high technical quality, and novel. However, the role of SLITRK2 in ALS dysregulation and synaptic dysfunction remains rather puzzling. Below are some points that the authors should address:

1) SLITRK2, as the authors mention in the manuscript, is a postsynaptic protein. Changes in its expression have been previously shown to primarily affect postsynaptic processes as well as the expression and density of presynaptic proteins, e.g., reduced vGlut1, SYP and SV2 (all of these found on the presynaptic neurons). In the present study, it is unclear, what the MNs expressing SLITRK2 are postsynaptic to. Here, the MNs are presynaptic, and this makes the NMJ experiments (both the morphological and the functional ones) very difficult to interpret. To my knowledge, SLITRK2 is expressed in cholinergic neurons, although its function is better understood in cortical glutamatergic neurotransmission. For clarity and to avoid confusions between the cortical and the spinal processes – which are already too many in the ALS field - the role of SLITRK2 at the NMJ should be elucidated.

2) The age of the donors at the time of the biopsy shown in Supplementary Figure 2 and Supplementary Table 5 should be addressed. The FUS-2 male appeared to be 17 at the time of biopsy and the control male, whose cells were used to generate the knock-in FUS line, was 8. However, all the control donors are age 60+. Could the authors explain such age mismatch? SLITRK2 is an X-linked gene which has been previously associated to neurodevelopmental diseases affecting preferentially males. The study linking C9orf72 mutations and SLITRK2 (Barbier M et al 2021) shows that SLITRK2 is associated with earlier onset. In line 226, the authors state "Comparison of the three different datasets showed limited overlap, perhaps due to the technical challenge of assessing low-count genes in axons and differences in the genetic background of the lines, but identified SLITRK2 as a consistently upregulated gene in FUS-ALS axons." Could this be also due to the very early onset of the donor chosen for the study?

Version 1:

Reviewer comments:

Reviewer #1

(Remarks to the Author)
Following revision of the manuscript, I am satisfied that the authors have with extensive efforts addressed all my concerns. I recommend this interesting and novel manuscript for publication.

Reviewer #2

(Remarks to the Author)
Authors have done a good job reading across all 3 reviews in responding with extra details especially around pre-post localisation, attempts at getting at MoA and textual clarification / moderation.

Reviewer #3

(Remarks to the Author)
The revised version of the manuscript is improved. The authors have addressed my concerns and I do not have further comments.

Open Access This Peer Review File is licensed under a Creative Commons Attribution 4.0 International License, which permits use, sharing, adaptation, distribution and reproduction in any medium or format, as long as you give appropriate credit to the original author(s) and the source, provide a link to the Creative Commons license, and indicate if changes were made.
In cases where reviewers are anonymous, credit should be given to 'Anonymous Referee' and the source.
The images or other third party material in this Peer Review File are included in the article's Creative Commons license, unless indicated otherwise in a credit line to the material. If material is not included in the article's Creative Commons license and your intended use is not permitted by statutory regulation or exceeds the permitted use, you will need to obtain permission directly from the copyright holder.
To view a copy of this license, visit https://creativecommons.org/licenses/by/4.0/

Point-to-point rebuttal - NCOMMS-25-75345 - "*SLITRK2* knockdown in human motor neurons rescues axonal and functional neuromuscular deficits in FUS-ALS" by De Jongh, Wiersema et al.

We would like to thank the Reviewers for their careful and positive assessment of our work and for giving us the opportunity to further improve our study. We have revised the manuscript according to the Reviewers' suggestions and have performed new microfluidic cultures, mouse studies, RNA sequencing, RNAscope in situ hybridization, immunohistochemical stainings, and other experiments and analyses (e.g. quantifications). These include, but are not limited to, experiments to confirm the axonal localization of mouse and human SLITRK2 (e.g. through RNAscope experiments), and to determine the functional pathogenic effects of enhanced SLITRK2 in axons and at the NMJ. As a result of additional experiments, main and Supplemental Figures have been modified, and new data have been added to several main and Supplementary Figures (Figure 1, 3, 4, 5, 8, S5-7, S12-14). Finally, we have made numerous additional textual revisions throughout the manuscript in response to the Reviewers' comments. Further, we have included a 'Conclusion and limitations' section to discuss important points raised by the Reviewers. Below we include a detailed response to the Reviewers' comments (in blue text):

Reviewer #1:

Synapse impairment at an early stage in ALS has been very much in the spotlight in the last few years. The molecular mechanisms that drive this impairment are not well understood, in part due to the current experimental limitations regarding interrogation of the synapse.

This paper uses a clever technique to circumvent that difficulty, by segregating axo-synaptic terminals from a cell body/dendritic neuronal compartment in microfluidic devices.

The authors employ a 3'UTR RNA sequencing approach to simultaneously identify upregulated mRNA expression and differentially expression of 3'UTR isoforms. Comparison of axonal RNA from FUS-ALS model MNs initially with healthy controls, and then with isogenic controls, identified SLITRK2 as a consistently upregulated gene in FUS-ALS axons. SLITRK2 knockdown was found to rescue axonal deficits in FUS-ALS motor neurons. Additionally, by using a novel and innovative co-culture NMJ model, the authors identify that SLITRK2 knockdown can rescue NMJ transmission. The authors do not describe a molecular mechanism to describe the role of SLITRK2 in this rescue.

This manuscript is clever, extremely interesting and very well written. I think this is an excellent addition to the knowledge in the field, and will be of interest to a large body of neurodegenerative disease researchers as well as those interested in axo-synaptic physiology.
We thank the reviewer for this positive assessment.

However there are a few points that I feel need to be addressed before this manuscript is suitable for publication, one of which is quite a big revision. If the team can address this point then I think this is very suitable for publication.

The major point that needs to be addressed is the elephant in the axon-chamber; the protein of interest, SLITRK2, in the axo synaptic chamber (a chamber which can only contain presynaptic terminals) is "a post synaptic protein" (line 234). As it stands in the paper this cannot be accurate. The sentence needs to be significantly reworded.

The Reviewer is correct that SLITRK2 is not only a post-synaptic protein. However, it is well known for it's role in the post-synaptic compartment in the context of CNS synapse development and function. To address the Reviewer's point, we have reworded the indicated sentence and provide further experimental evidence for a pre-synaptic localization of SLITRK2 in human motor neurons (see below).

However as this will be challenging previous understanding of the biology of this protein, it needs to be conclusively shown that the mRNA is localised in the axo-synaptic compartment, and ideally shown visually using specific probes that the 3'UTR isoforms are located in different axonal locations (proximal versus distal). The evidence in the manuscript is only presented at the protein level in axons. There are several ways the researchers could show this- most conclusive would be FISH for mRNA coupled with IF for an axon marker, or a presynaptic marker. Further proof would be to combine FISH for the mRNA combined with IF for a postsynaptic marker, showing conclusively that postsynaptic staining is present in the cell body chamber and not in the axo-synaptic chamber.
In the original manuscript, we indeed confirmed the expression of SLITRK2 protein in the axo-synaptic compartment. Further, our RNAseq analysis of axonal samples supported this observation at the mRNA level. To further address the Reviewer's concern, we have performed new RNAscope *in situ* hybridization experiments to detect *SLITRK2* mRNA in combination with immunocytochemistry for axonal markers (TUBB3) in our microfluidic motor neuron cultures (Figure 4G-H). For this we used a probe targeting the long 3'UTR isoform as this resulted in robust and specific labeling. These experiments confirmed the axonal localization of *SLITRK2* transcripts.

To further support the axonal localization of SLITRK2, we have performed immunohistochemistry for SLITRK2 on sciatic nerve tissue of adult WT mice to confirm axonal localization of SLITRK2 *in vivo* (Figure 4I). These additional data provide further support for an axonal/presynaptic localization of SLITRK2 in motor neurons.

Minor points:

Figure 1C and line 130 "MAP2, a dendritic marker, was only detected in the proximal compartment." In Figure 1C, merged image. The ISL1 and MAP2 representative colours are too close in colour to be distinguished. This makes it hard to verify that MAP2 is only present in the proximal chamber.
We have changed the colours for the ISL1 and MAP2 immunostaining to improve the contrast between both signals (Figure 1C).

Additionally, the bottom inlay needs to be moved further down- I can't tell if those are red or magenta puncta at the bottom of the image (I can't tell if there are MAP2 positive puncta visible in the grooves between compartments). Alternatively, an image of just MAP2 staining from the axo-synaptic compartment would prove this statement.
We have added an inlay to clearly visualize the axonal compartment, confirming the absence of MAP2 signal in the axonal compartment (Figure 1C).

Figure 1D; this schematic is not accurate. It's clear in the representative image from 1C that there are multiple axons in the cell body compartment (TUBB3 positive, MAP2 negative). Indeed, the authors do acknowledge this in the text (lines 165 and 166), and it is a known and accepted feature of using these devices. However, this should be represented in the schematic.
We agree with the Reviewer. We have updated the schematic representation to indicate the

presence of axons in the soma/cell body compartment (indicate compartment now as ‘ whole cell’; Figure 1D). We also now emphasize in the text that the ‘somatodendritic’ compartment contains axons (p. 7).

Figure 3A The schematic for Fig 3A is very helpful. It would be even more helpful if the nomenclature used for the rest of this figure was applied here (for example HC1, FUS-iso 1, etc).
We have revised the schematic to align with the nomenclature (e.g., HC1, FUS-1) used in the other parts of the figure (Figure 3A).

Figure 4 H. This figure is not mentioned in the manuscript text. I think this is due to a typo on line 281 (which should read Figure 4 G-H?). SLITRK2 counts are at 0 in 4 of 6 samples for the axon, and in 2 of 6 samples for the soma. I’m not sure this evidence is enough to say that “differential localization may suggest compartment-specific functions of the two SLITRK2 isoforms in MN”.
We acknowledge the Reviewer's concern regarding the variability and low read counts in our initial dataset. To address this, we performed RNA sequencing on additional HC somatodendritic and axonal samples. This analysis did not reveal robust differences in isoform localization between the somatodendritic and axonal compartments (Supplementary Figure S6G-H). We have therefore revised the text regarding compartment-specific isoform functions (p.13).

Fig 5 F and G. What happens when SLITRK2 is knocked down in HC cells?
To address this question, we performed SLITRK2 knockdown in HC MN and quantified axonal outgrowth (Supplementary Figure S7E-F). SLITRK2 knockdown did not induce changes in axonal outgrowth from HC MN, most likely because of the relatively low expression of SLITRK2 in HC motor neuron axons. We have included a ‘Conclusion and limitations’ section in which we describe this result and indicate that future studies are needed to further identify the physiological role of SLITRK2 (if any) in the axonal and presynaptic compartments.

Given the upregulation of SLITRK2 in FUS-ALS and to further address the Reviewer’s point, we next performed a gain-of-function experiment by overexpressing *SLITRK2* in HC MN (Figure 5G-H). This demonstrated that elevated levels of *SLITRK2* are sufficient to promote axonal outgrowth in HC MN and support a functional pathogenic role for enhanced SLITRK2 in increased axonal growth in FUS-ALS.

Reviewer #2:

Jongh and Wiersema etc al., investigated compartment-specific changes in RNA expression and regulation, identifying altered 3' UTR isoforms in FUS- ALS, including in genes SNCA and SLITRK2. They found SLITRK2 expression was upregulated in axonal compartment of FUS MNs, and its knockdown normalized axonal defects. Using a new neuromuscular microfluidics protocol, they showed SLITRK2 knockdown rescued neuromuscular junction (NMJ) signalling without affecting innervation or MN excitability.
We thank the reviewer for this assessment.

Following comments should be addressed.

1) Mechanistic insight into how FUS mutations affect expression of axonal SLITRK2; is it

mediated via transcriptional regulation and / or transport of mRNA? Given this is the novel finding of this study, this should be further investigated.
In line with the Reviewer's suggestion we have further studied how FUS mutations may affect the expression of SLITRK2. We exploited several of the reported mechanisms-of-action for FUS. Given the fact that FUS is a RNA binding protein, we first analyzed publicly available FUS CLIP-seq data for the presence of *SLITRK2* mRNA. Although we identified *SLITRK2* mRNA in three datasets (in raw data files), FUS-*SLITRK2* interactions were not validated in these studies or lacked statistical significance. We therefore performed FUS immunoprecipitation (IP) experiments from MN and SH-SY5Y samples but failed to show specific binding between FUS and *SLITRK2* mRNA (Supplementary Figure 14A–F). We therefore conclude that FUS does not bind *SLITRK2* mRNA in human motor neurons. Given the reported role of FUS in miRNA biology, we next assessed whether miRNAs predicted to target *SLITRK2* mRNA are dysregulated in FUS-ALS. miRNA prediction was performed using different algorithms (miRTargetLink 2.0, targetscan.org, miRDB and MiRTarBase) and the expression of miRNA candidates was examined in an unpublished dataset describing small non-coding RNA in FUS-ALS axons. However, none of the miRNAs predicted to target *SLITRK2* mRNA was altered in FUS-ALS samples (data not shown). Finally, we examined the axonal distribution of FUS and observed pronounced accumulation of FUS in axons in FUS-ALS as compared to FUS-iso MN (Supplementary Figure 7G-H). These observations suggest that rather than directly binding SLITRK2 mRNA FUS mutations may indirectly affect SLITRK2, for example by changing the localization of upstream gene expression regulators, several of which have been shown to bind FUS and mislocalize in FUS-ALS (e.g., Groen et al., 2013, Human Molecular Genetics; Blokhuis et al., 2016, Acta Neuropathologica). We have included these data and a discussion of the results in the Discussion section (p. 23-24). In addition, we have now included a few limitations of our study in a 'Conclusion and limitations' section in which describe these results and indicate that further work is needed to dissect the precise molecular mechanisms through which mutant FUS upregulates SLITRK2.

2) Figure 3J -L : Independent validation of SLITRK2 at mRNA and protein expression, which are crucial for downstream experiments. (3K) Are these expressed in fold change? If so, the changes appear to be modest, although numerically significant.
Additionally, there is a confusion regarding whether 2^-dCt (as mentioned in Figure) or 2^-ddCt (as mentioned in Methods) is being used.
The data shown in Figure 3J are presented as $2^{-\Delta Ct}$ values, representing relative mRNA expression levels normalized to our reference genes, rather than fold changes ($2^{-\Delta\Delta Ct}$).

We apologize for the confusion regarding the Methods section. We describe both the $2^{-\Delta Ct}$ and $2^{-\Delta\Delta Ct}$ calculations in the Methods because we utilize the $2^{-\Delta\Delta Ct}$ method in parts of the manuscript (e.g., Figure 1 and Supplementary Figure 14) to report fold changes. We chose $2^{-\Delta Ct}$ for Figure 3J in order to show baseline relative expression levels across the different cell lines.

Figure 3K-L: Could you please confirm if the authors have performed validation of SLITRK2 immunostaining? The representative images provided are difficult to interpret.
To address this point, we performed additional validation experiments in HC MN cultures (Supplementary Figure S5A). Omission of the primary SLITRK2 antibody did not result in labeling, while HC MN overexpressing SLITRK2 exhibited enhanced fluorescent signals compared to non-transfected cells.

Additionally, could you please provide information on the number of images and cells analyzed per biological replicate (legend)?
We have added the requested information to the legend.

Furthermore, it is intriguing that FUS2 iPS MN, which exhibited the least amount (or no substantial change) of SLITRK2 at the mRNA level, showed the highest level of change at the protein level. Authors should comment on this.
The indicated analysis of SLITRK2 at the mRNA level (Figure 3J) was performed on whole-cell lysates. At the whole-cell level the increase in SLITRK2 mRNA expression in the FUS-2 line appeared indeed modest. However, the RNAscope experiments that we performed in order to address a comment of Reviewer #1 confirm enrichment of *SLITRK2* transcripts in both the axonal and somatodendritic compartment of FUS-ALS MN for the FUS-2 (Figure 4G-H, Supplementary Figure S6). This observation is also in line with the increase in SLITRK2 protein expression in both the axonal and somatodendritic compartments (Figure 3K-L, Supplementary Figure S5B-E). While the different fold changes observed in the qPCR analysis of whole cell lysates may derive from technical or biological sample differences, our data show that also in the FUS2-2 line SLITRK2 is consistently upregulated. To address this point further, we now specifically acknowledge the variability of changes in SLITRK2 mRNA expression at the whole-cell level in the text (p. 12).

Does the SLITRK2 alteration affect only the axonal compartment? The authors should investigate the expression (mRNA and protein) of SLITRK2 in whole cell lysate.
To answer this question, we have studied SLITRK2 expression at both the mRNA and protein levels across the entire cell. At the mRNA level, our whole-cell RT-qPCR analysis (Figure 3J) demonstrates a significant increase in *SLITRK2* transcripts in FUS-ALS motor neurons compared to HC. This was further supported our RNAscope *in situ* hybridization data, which confirmed elevated SLITRK2 expression within the axonal and somatodendritic compartments (Supplementary Figure S6I-J). At the protein level, we quantified SLITRK2 expression in the axonal and somatodendritic compartment, demonstrating a corresponding and significant increase in FUS-ALS MN (Supplementary Figure S5D-E). Together, these data show that SLITRK2 is upregulated across the entire motor neuron.

3) Figure 4: SLITRK2 isoform usage. It is intriguing that the test compartmentalized usage of the isoform, however, the data presented suggested that in some of the samples, it is below the detection limit, making it challenging to interpret. Could the authors enhance the robustness of their findings by including additional samples and confirming the results via alternative methods, such as qPCR?
To address this point, we performed RNA sequencing on additional HC somatodendritic and axonal samples. This analysis did not reveal significant differences in isoform localization between the somatodendritic and axonal compartments (Supplementary Figure S6G-H). Therefore, we have revised the text regarding compartment-specific isoform functions. As an alternative method to visualize specific isoforms in distinct compartments we have performed RNAscope *in situ* hybridization for the long isoform of *SLITRK2* (Figure 4G-H and Supplementary Figure S6I-J). This showed that the long isoform is indeed present in the axons and that it is upregulated in both soma and axons in FUS-ALS MN as compared to controls.

4) Figure 7/8 - Given that this is a novel NMJ model developed, it is imperative to characterize the innervated AChR clusters that are co-localized with SYN and BTX. In this study, ICC was performed with a combined antibody of TUB and SYN, which makes it

challenging to precisely measure the percentage of innervated AChR clusters. Did SLITRK2 kd alter the number of innervated AchRs?
Combined immunostainings for TUBB3 and SYN are widely used to investigate the presynaptic part of the NMJ. However, to further validate our methodology and address the Reviewer's concern, we re-analyzed the co-cultures and independently investigated TUBB3 and SYP labelling (Supplementary Figure S13B). Quantification of innervated NMJs using this latter approach yielded results consistent with our original combined-channel (TUBB3+SYN) analysis (Supplementary Figure S13C).

SLITRK2 knockdown did not significantly alter the number of innervated AChR clusters (Supplementary Figure S13A).

Reviewer #3:

In the present manuscript, de Jongh and colleagues use in vitro patient-derived motor neurons to investigate compartment-specific changes in RNA expression. Here, they focus primarily on the ALS-triggering FUS mutation, known to cause changes in axonal transport and synaptic transmission. The authors use microfluidic devices combined with CEL-seq2 to analyse the transcriptome expression from either somato-dendritic or axonal compartments. Moreover, the CEL-seq2 approach allowed them to investigate alternative splicing in both compartments. DEG analysis revealed several differentially expressed genes between the two cell compartments (which were also compared with previously published datasets obtained with mouse cells). Comparisons between multiple control and FUS MNs revealed a consistent upregulation of the SLITRK2 gene in ALS MNs. The effects of SLITRK2 downregulation were then assessed in MN cultures alone, as well as in a fully humanised MN-NMJ culture using both morphological and Ca2+ functional analysis.
The majority of the experiments performed in this study is elegant, of high technical quality, and novel.
We thank the reviewer for this positive assessment.

However, the role of SLITRK2 in ALS dysregulation and synaptic dysfunction remains rather puzzling. Below are some points that the authors should address:

1) SLITRK2, as the authors mention in the manuscript, is a postsynaptic protein. Changes in its expression have been previously shown to primarily affect postsynaptic processes as well as the expression and density of presynaptic proteins, e.g., reduced vGlut1, SYP and SV2 (all of these found on the presynaptic neurons). In the present study, it is unclear, what the MNs expressing SLITRK2 are postsynaptic to. Here, the MNs are presynaptic, and this makes the NMJ experiments (both the morphological and the functional ones) very difficult to interpret. To my knowledge, SLITRK2 is expressed in cholinergic neurons, although its function is better understood in cortical glutamatergic neurotransmission. For clarity and to avoid confusions between the cortical and the spinal processes – which are already too many in the ALS field - the role of SLITRK2 at the NMJ should be elucidated.
The Reviewer righfully indicates that thus far SLITRK2 has predominantly been studied in the context of postsynaptic processes in studies focusing on CNS synapses. However, there is no data to support the idea that SLITRK2 is strictly a postsynaptic protein. As the Reviewer points our in our model motor neuron axons are the pre-synaptic compartment, whereas muscle cells are the post-synaptic cells. This is indeed different for the CNS neurons that have been studied thus far. Nevertheless, we agree with the Reviewer that this points required

further elucidation. Therefore, we revised the text in response to a comment of Reviewer #1 to ensure that we do not claim that SLITRK2 is solely a postsynaptic protein. We also performed new RNAscope *in situ* hybridization experiments to localize SLITRK2 mRNA in motor neuron axons and performed immunohistochemistry on adult mouse sciatic nerve sections to show axonal SLITRK2 expression (Figure 4G-I). Immunocytochemistry also confirmed that in our co-culture model, SLITRK2 localizes to the presynaptic terminal at the NMJ, co-localizing with the presynaptic vesicle marker SYP and acetylcholine receptor marker BTX (Figure 8B). At the functional level, overexpression of SLITRK2 in HC MN resulted in increased axonal outgrowth (Figure 5G-H), in line with its reported role in neurite outgrowth. We further demonstrated that SLITRK2 overexpression in HC MN significantly impaired neuromuscular transmission efficacy without altering NMJ integrity (Figure 8D-H). Together, these novel data establish a presynaptic localization and novel role for increased (pathogenic) SLITRK2 in motor neuron axons and at the NMJ (i.e. in neurotransmission). These overexpression phenotypes mimick the defects in FUS-ALS axons and the NMJ, thereby defining the pathogenic role of increased SLITRK2 expression in ALS. To further address this point, we describe in the Discussion section how SLITRK2 may exert its effects at the NMJ. Knockdown of endogenous SLITRK2 in HC MN did not result in obvious phenotypes in the assays tested in our study, most likely due to the low levels of SLITRK2 in HC motor neuron axons. To further address this point, we have now included a few limitations of our study in a 'Conclusion and limitations' section.

2) The age of the donors at the time of the biopsy shown in Supplementary Figure 2 and Supplementary Table 5 should be addressed. The FUS-2 male appeared to be 17 at the time of biopsy and the control male, whose cells were used to generate the knock-in FUS line, was 8. However, all the control donors are age 60+. Could the authors explain such age mismatch?
FUS-ALS (and especially C-terminal FUS mutations) is characterized by an early onset and enhanced disease progression. Such individuals are frequently diagnosed in our clinic but also in clinics around the world, and therefore included in our study as they are representative of the FUS-ALS patient population. To compensate for this young ALS patient we also included a young control. Further, we exploited isogenic controls to compensate for for example age differences.

SLITRK2 is an X-linked gene which has been previously associated to neurodevelopmental diseases affecting preferentially males. The study linking C9orf72 mutations and SLITRK2 (Barbier M et al 2021) shows that SLITRK2 is associated with earlier onset. In line 226, the authors state "Comparison of the three different datasets showed limited overlap, perhaps due to the technical challenge of assessing low-count genes in axons and differences in the genetic background of the lines, but identified SLITRK2 as a consistently upregulated gene in FUS-ALS axons." Could this be also due to the very early onset of the donor chosen for the study?
To address this point, we have evaluated the effect of donor age on the overall transcriptional variance in our dataset (see Extra Figure below). We stratified our samples by age group (<18 years, 18-59 years, >60 years) and performed a principal component analysis (PCA). As shown in our new eigencorplot (Extra Figure A), the principal component capturing the disease variance (PC9) is associated with the disease condition (ALS vs. Ctrl) but shows no significant correlation with the donor age group. Furthermore, PCA visualization (Extra Figure B) confirms that sample segregation along PC9 is driven by the FUS-ALS condition, with an absence of age-related clustering. Therefore, while differences in individual genetic backgrounds likely contribute to variability across datasets, these analyses confirm that the transcriptional variance we observe, including the consistent upregulation of SLITRK2, is

driven by the FUS-ALS disease status itself, rather than being a consequence of the donor's early age of onset.

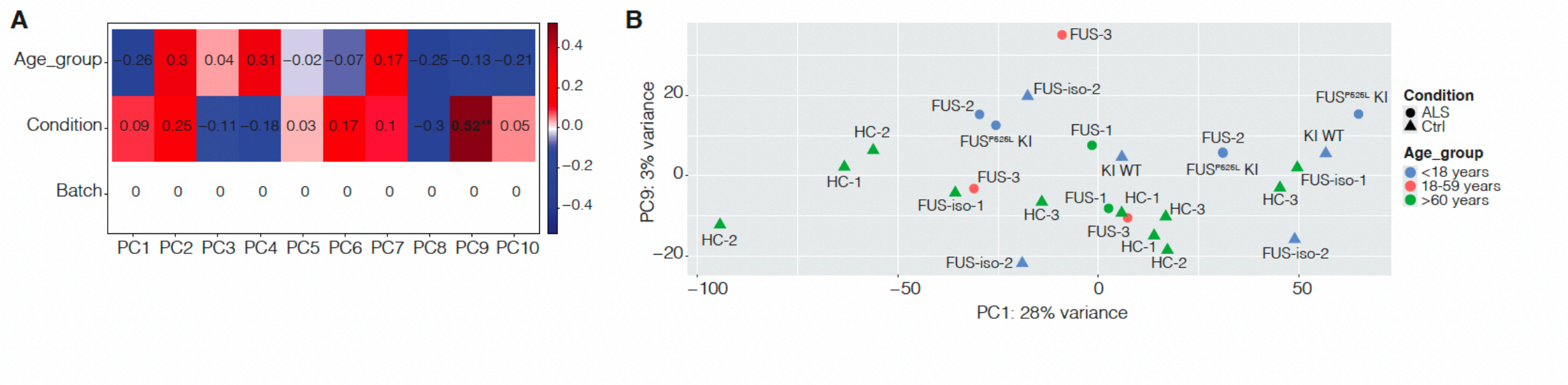

Extra figure: Evaluation of the effect of age on disease status.

A. Eigenccorplot illustrating the correlation between principal components (PC) 1–10 and biological variables: Condition (ALS vs. Ctrl) and Age_group (<18 years, 18-59 years, >60 years) after batch effect correction. The analysis identifies PC9 as a component with a strong association with the 'Condition' variable, while showing no significant correlation with 'Age_group'.

B. PCA plot visualizing the distribution of samples along PC1 and PC9. Samples are coded by Condition (ALS vs. Ctrl) and Age_group (<18 years, 18-59 years, >60 years). The segregation of samples by condition along PC9, in the absence of age-related clustering, confirms that the identified transcriptional variance is driven by disease status rather than donor age.
