# Supplementary material for: SLITRK2 knockdown rescues axonal and functional neuromuscular junction deficits in human iPSC-derived mutant FUS-ALS motor neurons
Source: Nat Commun. 2026 Oct 15;17:11662. doi: 10.1038/s41467-026-78419-7 (PMC13759529; doi:10.1038/s41467-026-78419-7)
Supplement: Supplementary file 2 [file 41467_2026_78419_MOESM1_ESM.pdf]

Supplementary Information

SLITRK2 knockdown rescues axonal and functional neuromuscular junction deficits in human iPSC-derived mutant FUS-ALS motor neurons.

Rianne de Jongh[1,5,7], Anna F. Wiersema[1,7], Charlotte H. van Dijk[1], Roos C. Verdegaal[1], Mareine M. J. Smulders[1], Onur Basak[1], Jelle van Os[1], Sofie Peeters[1], Laura Olmedo-Martínez[2], Lauri M. Bloemenkamp[1], Keith M. Garner[1], Sandrine Da Cruz[2], Max Koppers[3,6], Alessandro Iuliano[4], W.W.M. Pim Pijnappel[4], Kevin P. Kenna[1], and R. Jeroen Pasterkamp[1*]

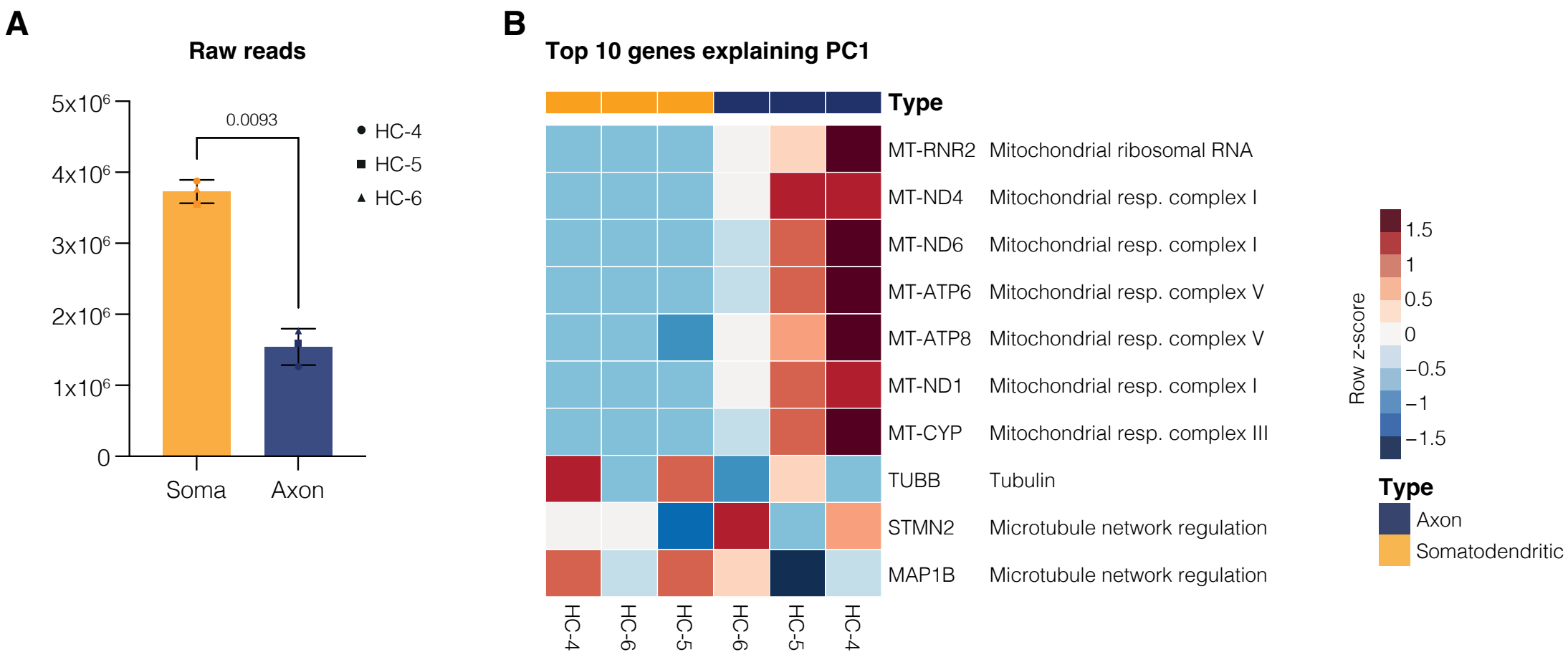
A
Raw reads
5x10⁶
4x10⁶
3x10⁶
2x10⁶
1x10⁶
0
0.0093
HC-4
HC-5
HC-6
Soma
Axon
B
Top 10 genes explaining PC1
Type
MT-RNR2 Mitochondrial ribosomal RNA
MT-ND4 Mitochondrial resp. complex I
MT-ND6 Mitochondrial resp. complex I
MT-ATP6 Mitochondrial resp. complex V
MT-ATP8 Mitochondrial resp. complex V
MT-ND1 Mitochondrial resp. complex I
MT-CYP Mitochondrial resp. complex III
TUBB Tubulin
STMN2 Microtubule network regulation
MAP1B Microtubule network regulation
HC-4
HC-6
HC-5
HC-6
HC-5
HC-4
Row z-score
1.5
1
0.5
0
−0.5
−1
−1.5
Type
Axon
Somatodendritic

Supplementary Figure S1: Quality control and characterization of the axonal versus somatodendritic transcriptome in HC motor neurons.

A. Number of mapped reads in somatodendritic and axonal samples. Data is presented as mean ± SD (*n*=3 biological replicates, *t*=1 technical replicate). Paired two-tailed T-test. **B.** Heatmap displaying normalized expression levels of the top 10 genes explaining PC1 shown in Figure 2A. Each gene is annotated with its corresponding functional class. Source data are provided as a Source Data file.

A

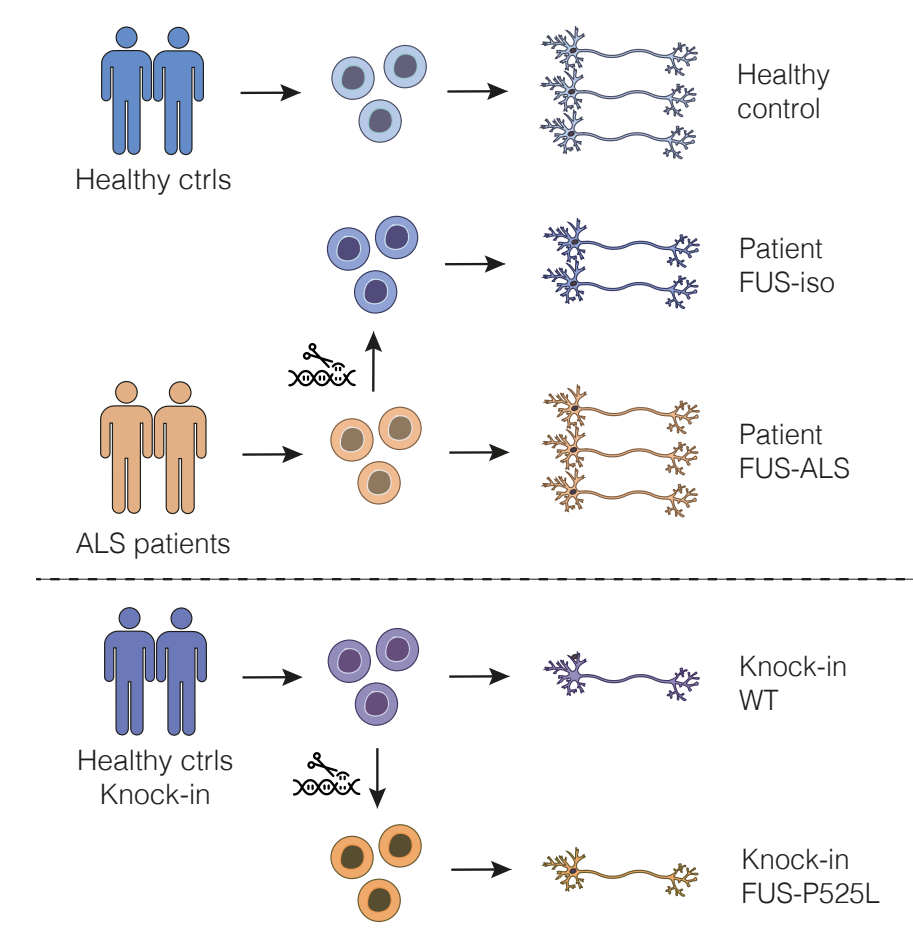

B

Line	Mutation	Gender	Age of Biopsy	Repro-gramming	Gene editing
HC-1	-	Female	60	Sendai	-
HC-2	-	Male	63	Sendai	-
HC-3	-	Male	62	Sendai	-
FUS-iso-1	-	Male	71	Sendai	CRISPR/Cas9
FUS-iso-2	-	Male	17	Sendai	CRISPR/Cas9
FUS-1	R521H	Male	71	Sendai	-
FUS-2	P525L	Male	17	Sendai	-
FUS-3	R521C	Female	32	Sendai	-
Knock-in WT	-	Male	8	Lentivirus	-
P525L Knock-in	P525L	Male	8	Lentivirus	TALEN

C

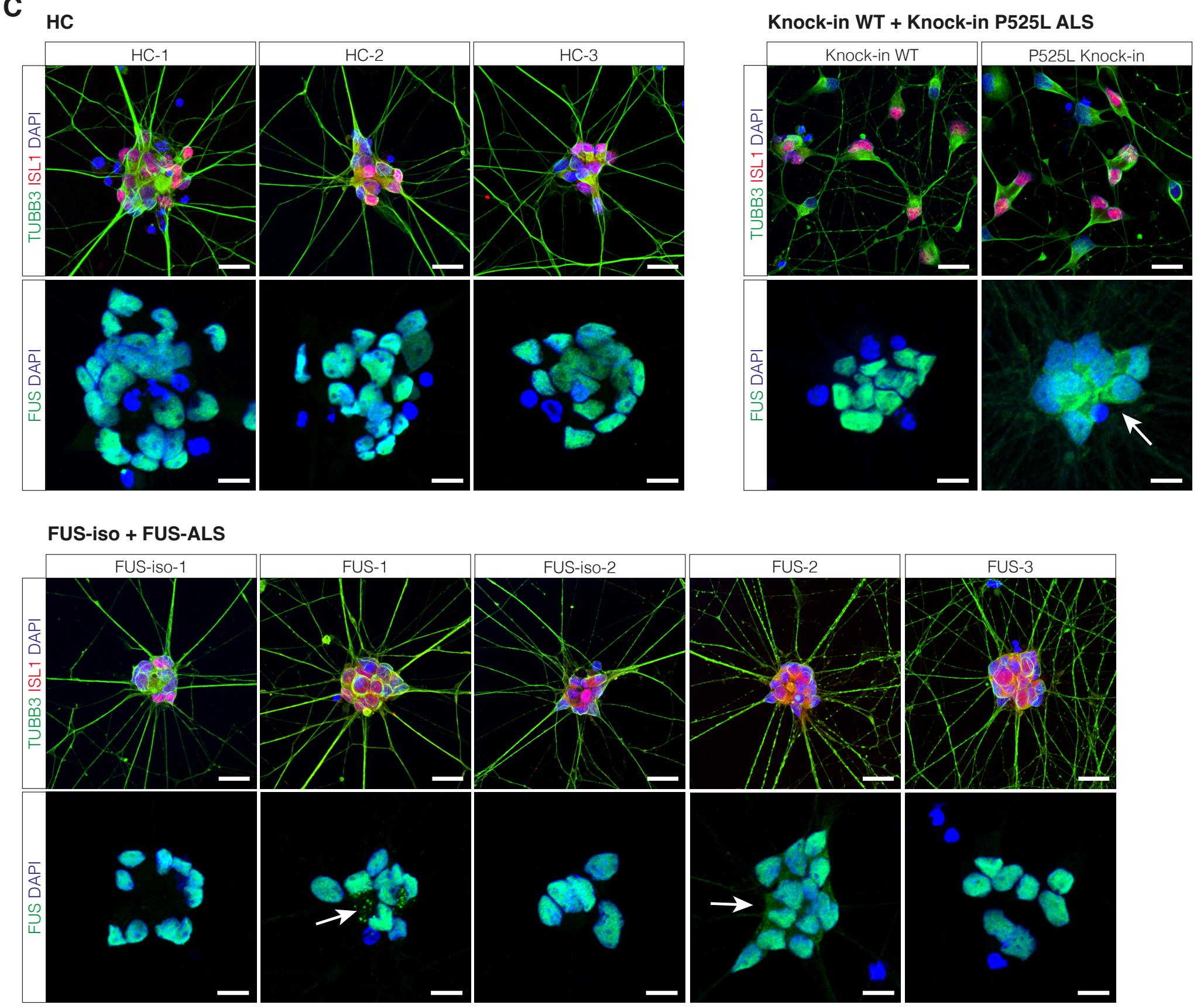

Supplementary Figure S2: Characterization iPSC lines.

A. Schematic overview of iPSC-derived lines used in this study, including HC, FUS-ALS, FUS-iso, FUS^{P525L} knock-in and knock-in WT lines. **B.** Table summarizing genetic and clinical information for the iPSC-derived lines presented in A, including details on *FUS* mutation, gender of the donor, age at biopsy, reprogramming method and gene editing method (if applicable). **C.** Immunocytochemical analysis of MN markers TUBB3 and ISL1 (upper) and FUS expression (lower) in MN derived from all iPSC lines. Arrows indicate cytoplasmic mislocalization of FUS. Scale bars: 20 µm (upper), 10 µm (lower).

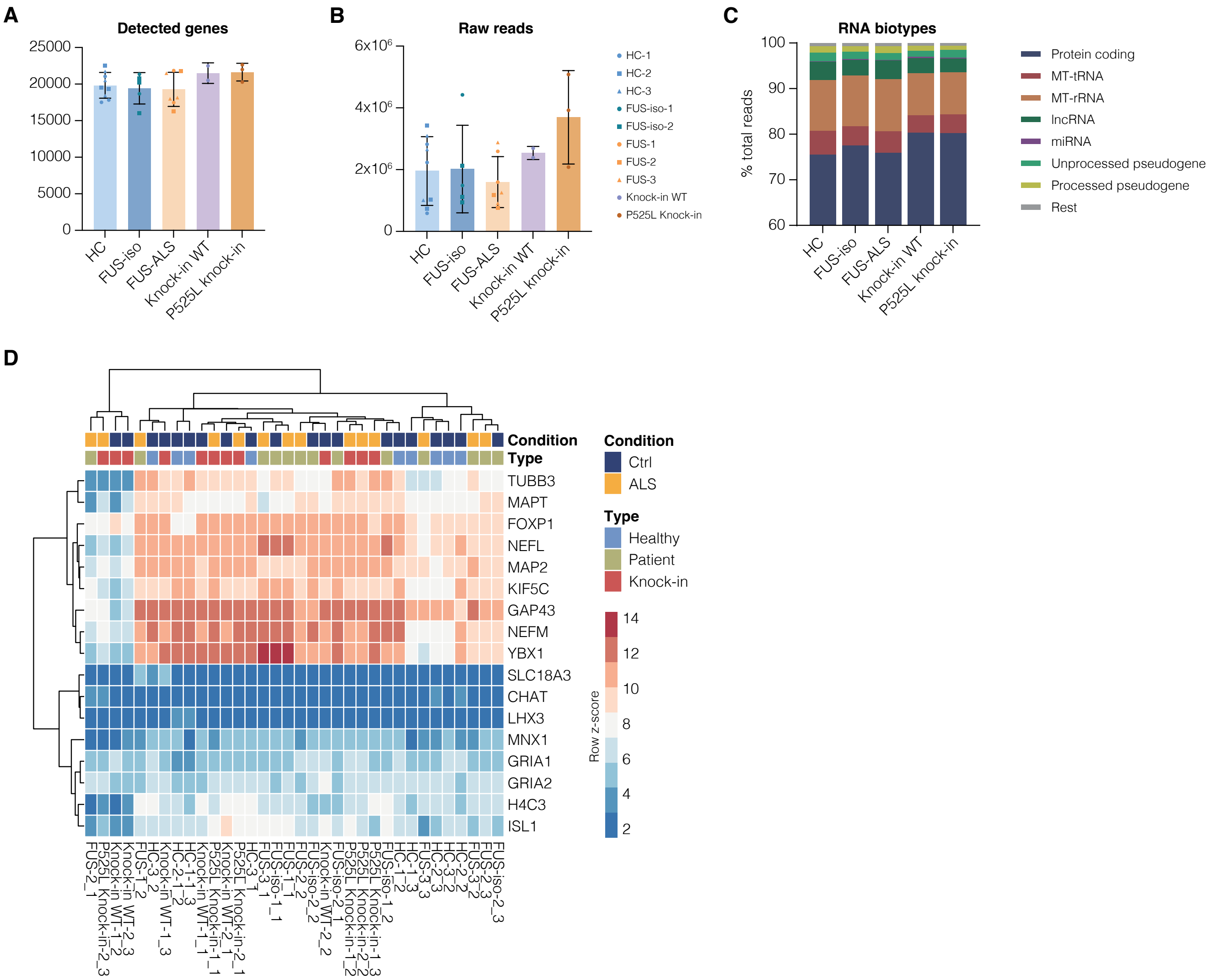

A
Detected genes
25000
20000
15000
10000
5000
0
HC
FUS-iso
FUS-ALS
Knock-in WT
P525L knock-in
B
Raw reads
6x10⁶
4x10⁶
2x10⁶
0
HC-1
HC-2
HC-3
FUS-iso-1
FUS-iso-2
FUS-1
FUS-2
FUS-3
Knock-in WT
P525L Knock-in
C
RNA biotypes
% total reads
100
90
80
70
60
Protein coding
MT-tRNA
MT-rRNA
lncRNA
miRNA
Unprocessed pseudogene
Processed pseudogene
Rest
D
Condition
Type
Ctrl
ALS
Healthy
Patient
Knock-in
TUBB3
MAPT
FOXP1
NEFL
MAP2
KIF5C
GAP43
NEFM
YBX1
SLC18A3
CHAT
LHX3
MNX1
GRIA1
GRIA2
H4C3
ISL1
Row z-score
14
12
10
8
6
4
2
FUS-2_1
P525L Knock-in-2_3
Knock-in WT-1_2
Knock-in WT-2_3
FUS-1_2
HC-3_2
Knock-in WT-1_3
HC-2_2
HC-1_3
Knock-in WT-1_1
P525L Knock-in-1_1
Knock-in WT-2_1
P525L Knock-in-2_1
HC-3_1
FUS-3_1
FUS-iso-1_1
FUS-1_1
FUS-2_2
FUS-iso-2_2
Knock-in WT-2_2
FUS-iso-2_1
P525L Knock-in-2_2
P525L Knock-in-1_3
P525L Knock-in-1_2
FUS-iso-1_2
HC-1_2
HC-1_3
FUS-3_3
HC-2_3
HC-3_2
HC-2_1
FUS-3_2
FUS-2_3
FUS-iso-2_3

Supplementary Figure S3: Quality control and characterization of axonal RNA-seq data.

A-B. Number of detected genes (A) and number of mapped reads (B) in axonal samples from different experimental conditions. Data is presented as mean ± SD. Biological (*n*) and technical (*t*) replicates per group: HC (n=3, t=3,3,3), FUS-iso (n=2, t=2,3), FUS-ALS (n=3, t=2,2,3), Knock-in WT (n=1, t=2), P525L knock-in (n=1, t=3). One-Way ANOVA (p = 0.3524). **C.** RNA biotype classification in axonal samples from different experimental conditions. Data is presented as mean. HC (n=3, t=3,3,3), FUS-iso (n=2, t=2,3), FUS-ALS (n=3, t=2,2,3), Knock-in WT (n=1, t=2), P525L knock-in (n=1, t=3). Two-way ANOVA (p = 0.1363). **D.** Heatmap showing normalized expression levels of selected established somatodendritic and axonal marker genes across all individual axonal samples. Source data are provided as a Source Data file.

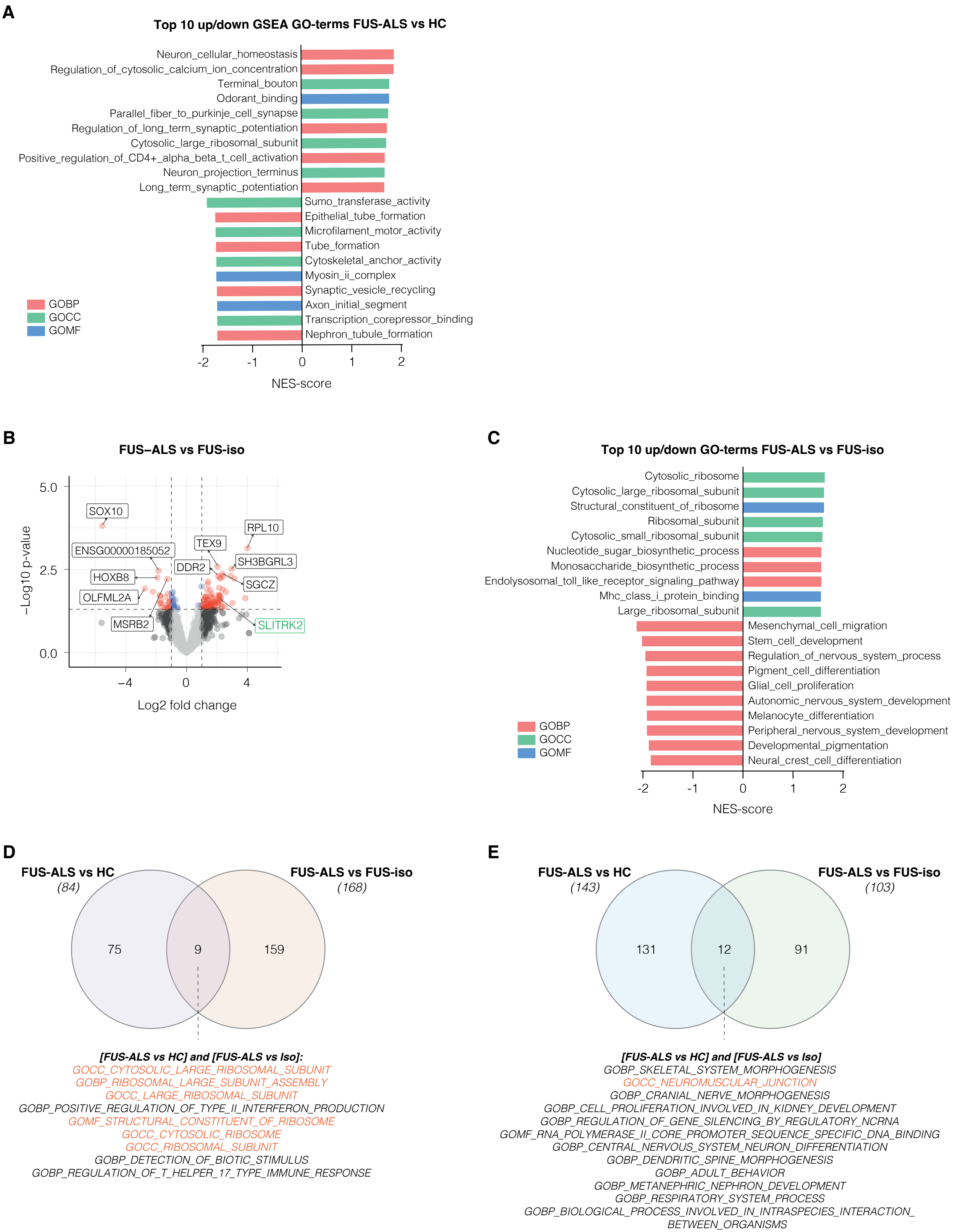
A
Top 10 up/down GSEA GO-terms FUS-ALS vs HC
Neuron_cellular_homeostasis
Regulation_of_cytosolic_calcium_ion_concentration
Terminal_bouton
Odorant_binding
Parallel_fiber_to_purkinje_cell_synapse
Regulation_of_long_term_synaptic_potentiation
Cytosolic_large_ribosomal_subunit
Positive_regulation_of_CD4+_alpha_beta_t_cell_activation
Neuron_projection_terminus
Long_term_synaptic_potentiation
Sumo_transferase_activity
Epithelial_tube_formation
Microfilament_motor_activity
Tube_formation
Cytoskeletal_anchor_activity
Myosin_ii_complex
Synaptic_vesicle_recycling
Axon_initial_segment
Transcription_corepressor_binding
Nephron_tubule_formation
GOBP
GOCC
GOMF
-2
-1
0
1
2
NES-score
B
FUS–ALS vs FUS-iso
5.0
2.5
0.0
−Log10 p-value
SOX10
ENSG00000185052
HOXB8
OLFML2A
MSRB2
TEX9
DDR2
RPL10
SH3BGRL3
SGCZ
SLITRK2
−4
0
4
Log2 fold change
C
Top 10 up/down GO-terms FUS-ALS vs FUS-iso
Cytosolic_ribosome
Cytosolic_large_ribosomal_subunit
Structural_constituent_of_ribosome
Ribosomal_subunit
Cytosolic_small_ribosomal_subunit
Nucleotide_sugar_biosynthetic_process
Monosaccharide_biosynthetic_process
Endolysosomal_toll_like_receptor_signaling_pathway
Mhc_class_i_protein_binding
Large_ribosomal_subunit
Mesenchymal_cell_migration
Stem_cell_development
Regulation_of_nervous_system_process
Pigment_cell_differentiation
Glial_cell_proliferation
Autonomic_nervous_system_development
Melanocyte_differentiation
Peripheral_nervous_system_development
Developmental_pigmentation
Neural_crest_cell_differentiation
GOBP
GOCC
GOMF
-2
-1
0
1
2
NES-score
D
FUS-ALS vs HC
(84)
FUS-ALS vs FUS-iso
(168)
75
9
159
[FUS-ALS vs HC] and [FUS-ALS vs Iso]:
GOCC_CYTOSOLIC_LARGE_RIBOSOMAL_SUBUNIT
GOBP_RIBOSOMAL_LARGE_SUBUNIT_ASSEMBLY
GOCC_LARGE_RIBOSOMAL_SUBUNIT
GOBP_POSITIVE_REGULATION_OF_TYPE_II_INTERFERON_PRODUCTION
GOMF_STRUCTURAL_CONSTITUENT_OF_RIBOSOME
GOCC_CYTOSOLIC_RIBOSOME
GOCC_RIBOSOMAL_SUBUNIT
GOBP_DETECTION_OF_BIOTIC_STIMULUS
GOBP_REGULATION_OF_T_HELPER_17_TYPE_IMMUNE_RESPONSE
E
FUS-ALS vs HC
(143)
FUS-ALS vs FUS-iso
(103)
131
12
91
[FUS-ALS vs HC] and [FUS-ALS vs Iso]
GOBP_SKELETAL_SYSTEM_MORPHOGENESIS
GOCC_NEUROMUSCULAR_JUNCTION
GOBP_CRANIAL_NERVE_MORPHOGENESIS
GOBP_CELL_PROLIFERATION_INVOLVED_IN_KIDNEY_DEVELOPMENT
GOBP_REGULATION_OF_GENE_SILENCING_BY_REGULATORY_NCRNA
GOMF_RNA_POLYMERASE_II_CORE_PROMOTER_SEQUENCE_SPECIFIC_DNA_BINDING
GOBP_CENTRAL_NERVOUS_SYSTEM_NEURON_DIFFERENTIATION
GOBP_DENDRITIC_SPINE_MORPHOGENESIS
GOBP_ADULT_BEHAVIOR
GOBP_METANEPHRIC_NEPHRON_DEVELOPMENT
GOBP_RESPIRATORY_SYSTEM_PROCESS
GOBP_BIOLOGICAL_PROCESS_INVOLVED_IN_INTRASPECIES_INTERACTION_
BETWEEN_ORGANISMS

Supplementary Figure S4: DEGs and comparative GSEA of axonal transcriptomes from FUS-ALS, HC and FUS-iso motor neurons.

A. GSEA results showing the top 10 positive and negatively enriched GO-terms in the axonal transcriptome of FUS-ALS compared to HC MN. Terms are color-coded by GO domain: biological processes (red), cellular components (green) and molecular functions (blue). **B.** Volcano plot showing DEGs in the axonal transcriptome of FUS-ALS compared to FUS-iso MN. Differential expression analysis was performed using DESeq2 (two-sided Wald test with Benjamini–Hochberg FDR correction; $p<0.05$, $|Log2FC|>1$). The top up- and downregulated proteins are labeled. **C.** GSEA results showing the top 10 positive and negatively enriched GO-terms in the axonal transcriptome of FUS-ALS compared to FUS-iso MN. Terms are color-coded by GO domain: biological processes (red), cellular components (green) and molecular functions (blue). **D-E.** Venn diagram depicting the shared significantly positively enriched (D) and negatively enriched (E) GO-terms identified in GSEA of FUS-ALS vs HC MN axons and FUS-ALS vs FUS-iso MN axons. GO terms corresponding to the representative pathways highlighted in Figure 3F-G are indicated in red.

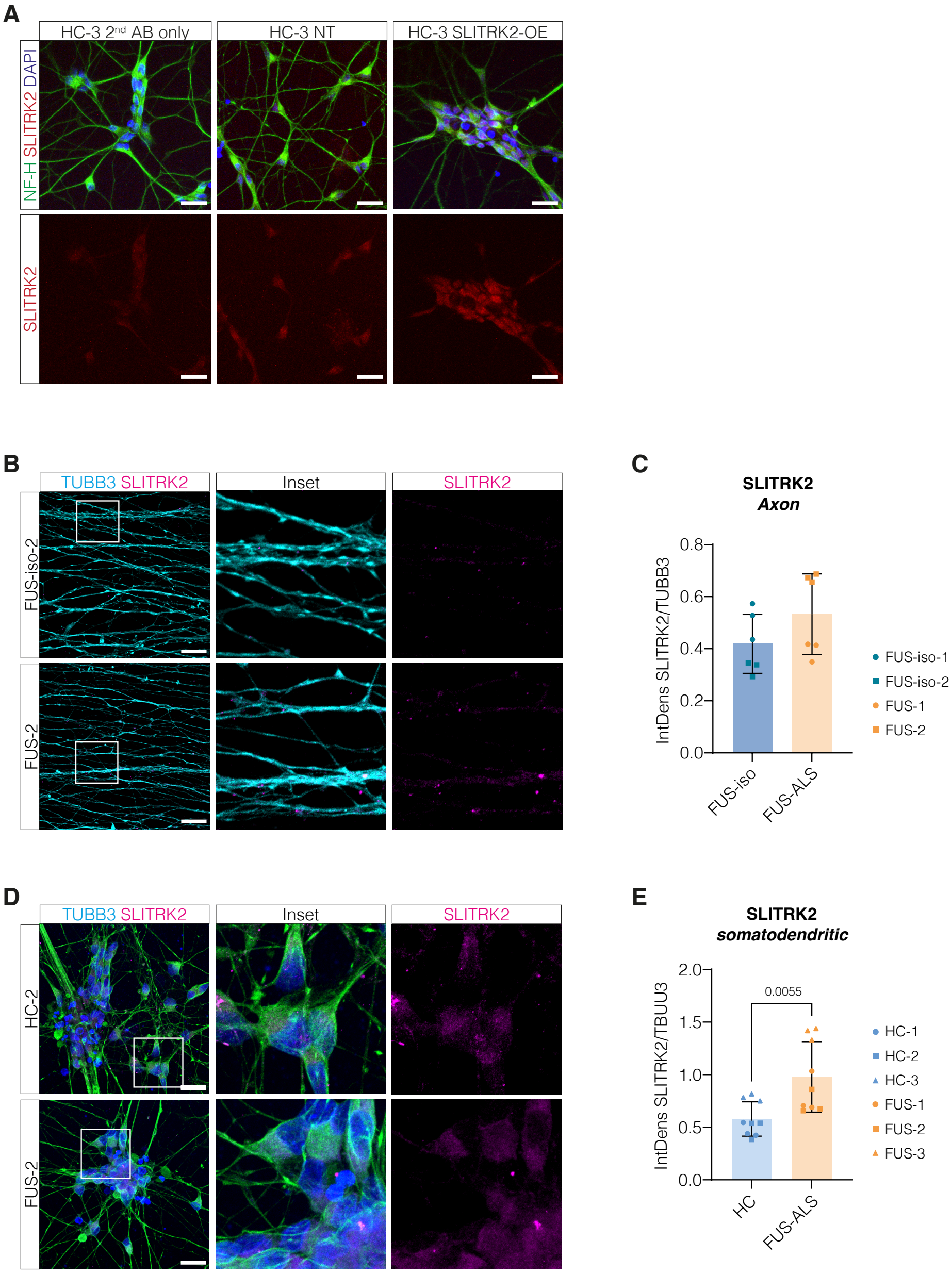
A
HC-3 2nd AB only
HC-3 NT
HC-3 SLITRK2-OE
NF-H SLITRK2 DAPI
SLITRK2
B
TUBB3 SLITRK2
Inset
SLITRK2
FUS-iso-2
FUS-2
C
SLITRK2
Axon
IntDens SLITRK2/TUBB3
FUS-iso
FUS-ALS
FUS-iso-1
FUS-iso-2
FUS-1
FUS-2
D
TUBB3 SLITRK2
Inset
SLITRK2
HC-2
FUS-2
E
SLITRK2
somatodendritic
IntDens SLITRK2/TBUU3
0.0055
HC
FUS-ALS
HC-1
HC-2
HC-3
FUS-1
FUS-2
FUS-3

Supplementary Figure S5: SLITRK2 protein expression in somatic and axonal compartments of motor neurons.

A. Representative immunofluorescence images validating SLITRK2 antibody staining in HC MN. Shown are negative controls (left: secondary antibody only), HC NT (center) and SLITRK2-OE in HC (right). MN were co-stained with NF-H and DAPI. No staining was observed in the absence of primary antibody and overexpression of SLITRK2 induced enhanced immunostaining, confirming the specificity of the staining. Scale bar: 20 µm. **B.** SLITRK2 protein expression within axons of TUBB3+ MN axons from FUS-iso and FUS-ALS patients. Inset shows magnification of SLITRK2 protein expression. Scale bar: 20 µm. **C.** Quantification of axonal SLITRK2 expression in TUBB3+ FUS-iso and FUS-ALS MN normalized to TUBB3 expression using integrated density measurements. Data is presented as mean ± SD (n=2 biological replicates, t=3 technical replicates). Paired two-tailed T-test (p = 0.3367). **D.** SLITRK2 protein expression within the soma of TUBB3+ MN from HC and FUS-ALS patients. Inset shows magnification of SLITRK2 protein expression. Scale bar: 20 µm. **E.** Quantification of somatic SLITRK2 expression in TUBB3+ FUS-ALS and HC MN normalized to TUBB3 expression using integrated density measurements. Data is presented as mean ± SD (n=3, t=3). Unpaired two-tailed T-test. Source data are provided as a Source Data file.

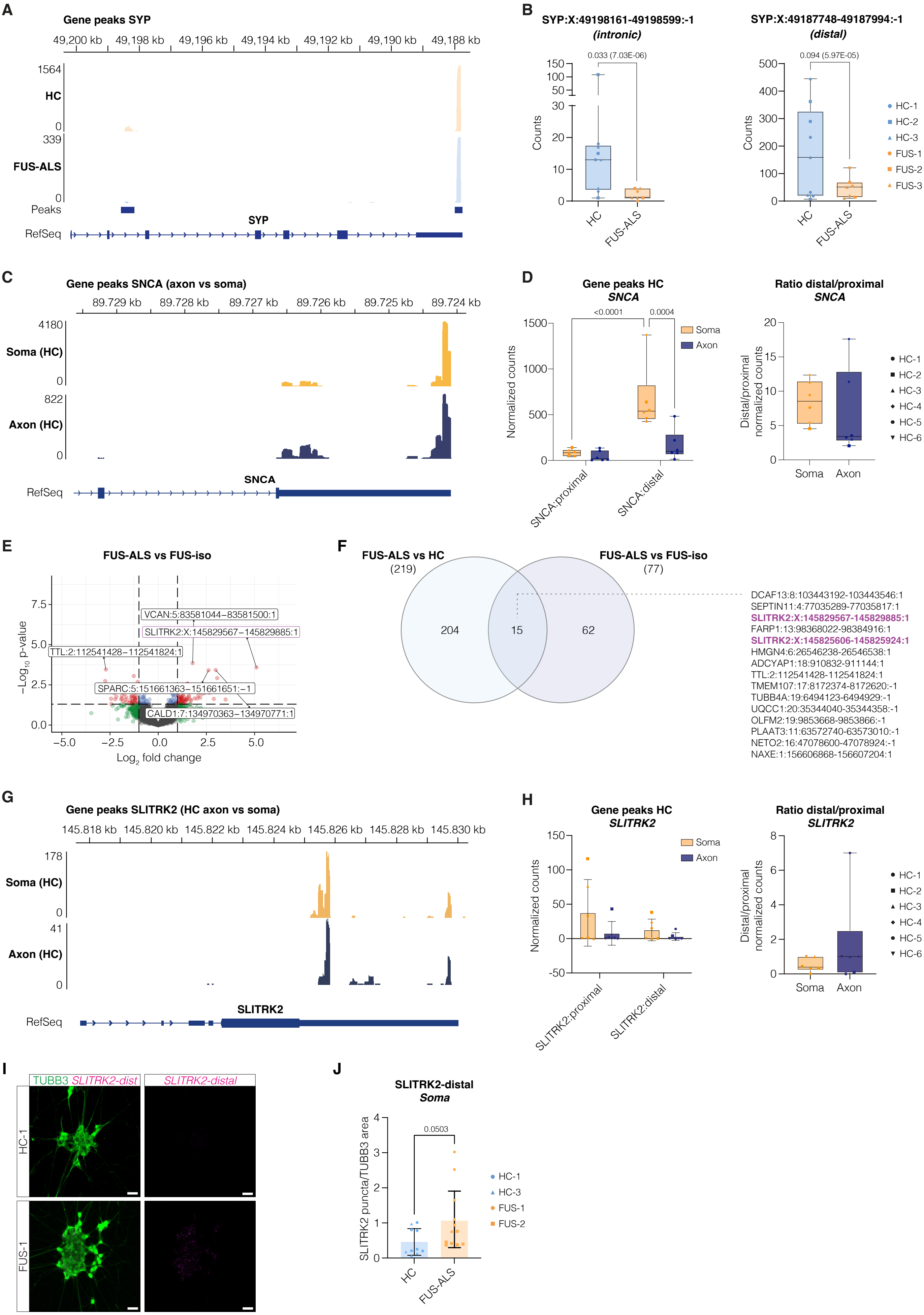

A
Gene peaks SYP
49,200 kb
49,198 kb
49,196 kb
49,194 kb
49,192 kb
49,190 kb
49,188 kb
1564
HC
0
339
FUS-ALS
0
Peaks
SYP
RefSeq
B
SYP:X:49198161-49198599:-1
(intronic)
0.033 (7.03E-06)
Counts
HC
FUS-ALS
SYP:X:49187748-49187994:-1
(distal)
0.094 (5.97E-05)
HC-1
HC-2
HC-3
FUS-1
FUS-2
FUS-3
C
Gene peaks SNCA (axon vs soma)
89.729 kb
89.728 kb
89.727 kb
89.726 kb
89.725 kb
89.724 kb
4180
Soma (HC)
822
Axon (HC)
SNCA
RefSeq
D
Gene peaks HC
SNCA
<0.0001
0.0004
Soma
Axon
Normalized counts
SNCA:proximal
SNCA:distal
Ratio distal/proximal
SNCA
Distal/proximal normalized counts
Soma
Axon
HC-1
HC-2
HC-3
HC-4
HC-5
HC-6
E
FUS-ALS vs FUS-iso
VCAN:5:83581044−83581500:1
SLITRK2:X:145829567−145829885:1
TTL:2:112541428−112541824:1
SPARC:5:151661363−151661651:−1
CALD1:7:134970363−134970771:1
−Log10 p-value
Log2 fold change
F
FUS-ALS vs HC
(219)
FUS-ALS vs FUS-iso
(77)
204
15
62
DCAF13:8:103443192-103443546:1
SEPTIN11:4:77035289-77035817:1
SLITRK2:X:145829567-145829885:1
FARP1:13:98368022-98384916:1
SLITRK2:X:145825606-145825924:1
HMGN4:6:26546238-26546538:1
ADCYAP1:18:910832-911144:1
TTL:2:112541428-112541824:1
TMEM107:17:8172374-8172620:-1
TUBB4A:19:6494123-6494929:-1
UQCC1:20:35344040-35344358:-1
OLFM2:19:9853668-9853866:-1
PLAAT3:11:63572740-63573010:-1
NETO2:16:47078600-47078924:-1
NAXE:1:156606868-156607204:1
G
Gene peaks SLITRK2 (HC axon vs soma)
145.818 kb
145.820 kb
145.822 kb
145.824 kb
145.826 kb
145.828 kb
145.830 kb
178
Soma (HC)
41
Axon (HC)
SLITRK2
RefSeq
H
Gene peaks HC
SLITRK2
Soma
Axon
Normalized counts
SLITRK2:proximal
SLITRK2:distal
Ratio distal/proximal
SLITRK2
Distal/proximal normalized counts
Soma
Axon
HC-1
HC-2
HC-3
HC-4
HC-5
HC-6
I
TUBB3 SLITRK2-dist
SLITRK2-distal
HC-1
FUS-1
J
SLITRK2-distal
Soma
0.0503
SLITRK2 puncta/TUBB3 area
HC
FUS-ALS
HC-1
HC-3
FUS-1
FUS-2

Supplementary Figure S6: Supporting data for APA analysis in FUS-ALS motor neurons.

A. IGV visualization of *SYP* gene peaks mapped to the RefSeq-Select annotation, demonstrating peaks corresponding to an intronic and distal 3' UTR. **B.** Boxplot showing counts of the intronic (*SYP:X:49198161-49198599:-1*) and distal (*SYP:X:49187748-49187994:-1*) *SYP* gene peaks in HC and FUS-ALS MN axons. Data is presented as mean ± SD. Biological (n) and technical (t) replicates per group: HC (n=3, t=3,3,3), FUS-ALS (n=3, t=2,2,3). Statistical analysis by DESeq2 (two-sided Wald test with Benjamini–Hochberg FDR correction). p-values are shown with adjusted p-values in brackets. **C.** IGV visualization of *SNCA* gene peaks mapped to the RefSeq-Select annotation in HC axon and soma samples. **D.** Boxplot showing counts of the proximal and distal *SNCA* gene peaks (left) and the ratio distal/proximal counts (right) in HC axon and soma samples (n=6, t=1). Two-Way ANOVA (left), Paired two-tailed T-test (right; p = 0.3579). **E.** Volcano plot showing differential usage of 3' UTR isoforms comparing the axonal transcriptome of FUS-ALS versus FUS-iso MN analyzed by DESeq2 (two-sided Wald test with Benjamini–Hochberg FDR correction; p<0.05, |Log2FC|>1). The top up- and downregulated gene peaks are labeled. *SLITRK2* peak is indicated in magenta. **F.** Venn diagram depicting the shared differentially identified gene peaks in FUS-ALS vs HC and FUS-ALS vs FUS-iso comparisons. **G.** IGV visualization of *SLITRK2* gene peaks mapped to the RefSeq-Select annotation in HC axon versus soma samples. **H.** Boxplot showing counts of the proximal and distal *SLITRK2* gene peaks (left) and the ratio distal/proximal counts (right) in HC axon and soma samples (n=6, t=1). Two-Way ANOVA (left; p = 0.3720), Wilcoxon test (right; p = 0.5000). **I.** Representative RNAscope images showing the expression of the distal (long) SLITRK2 3' UTR isoform within TUBB3+ MN soma from HC and FUS-ALS lines. Scale bar: 20 µm. **J.** Quantification of the distal (long) *SLITRK2* isoform expression in HC and FUS-ALS MN soma, normalized to the TUBB3+ area. Data is presented as mean ± SD. HC (n=2, t=6,4), FUS-ALS (n=2, t=6,6). Two-tailed Mann-Whitney test. Source data are provided as a Source Data file.

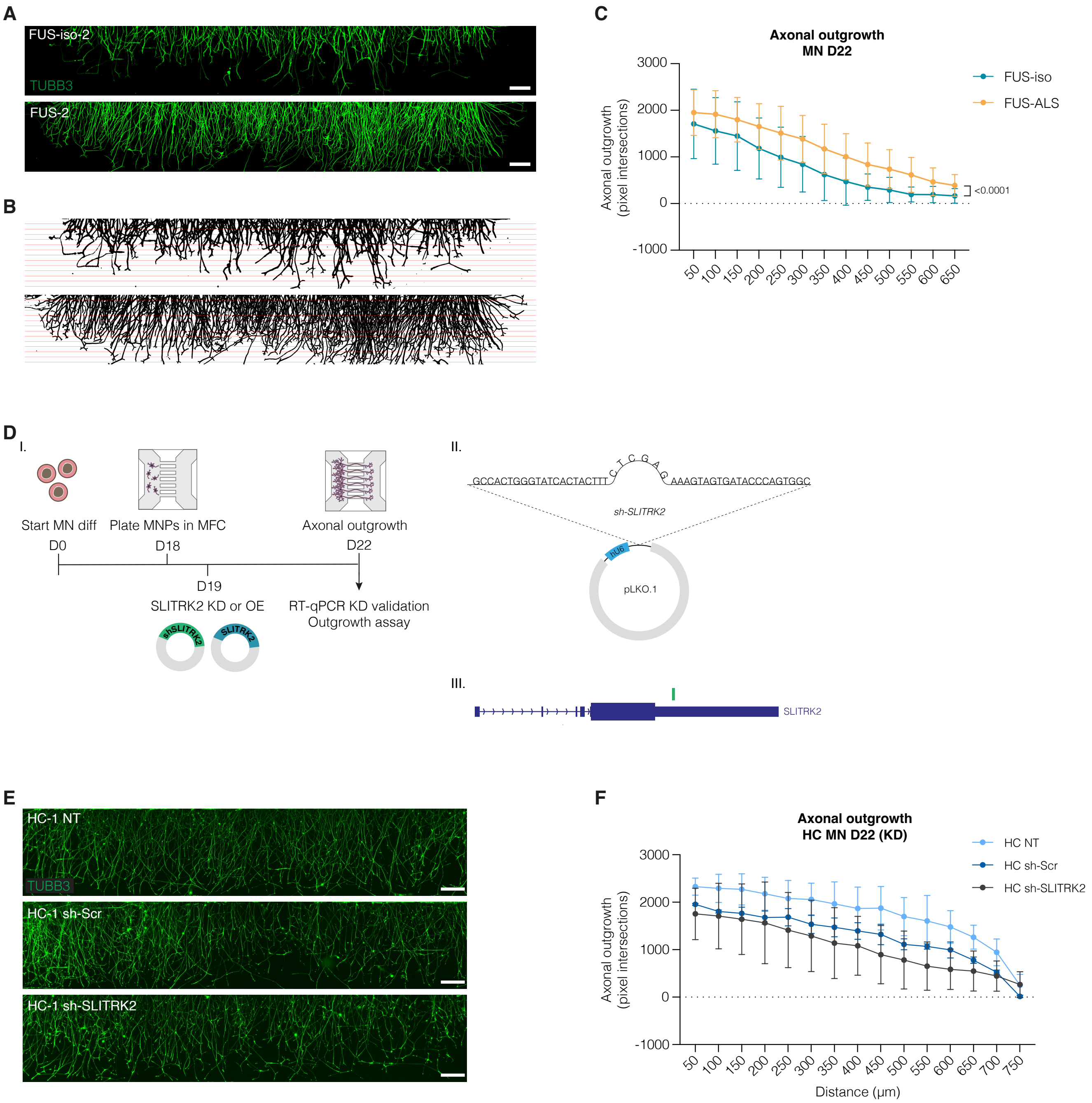

A
FUS-iso-2
TUBB3
FUS-2
B
C
Axonal outgrowth
MN D22
Axonal outgrowth
(pixel intersections)
3000
2000
1000
0
-1000
50
100
150
200
250
300
350
400
450
500
550
600
650
FUS-iso
FUS-ALS
<0.0001
D
I.
Start MN diff
D0
Plate MNPs in MFC
D18
D19
SLITRK2 KD or OE
shSLITRK2
SLITRK2
Axonal outgrowth
D22
RT-qPCR KD validation
Outgrowth assay
II.
GCCACTGGGTATCACTACTTT
CTCGAG
AAAGTAGTGATACCCAGTGGC
sh-SLITRK2
hU6
pLKO.1
III.
SLITRK2
E
HC-1 NT
TUBB3
HC-1 sh-Scr
HC-1 sh-SLITRK2
F
Axonal outgrowth
HC MN D22 (KD)
Axonal outgrowth
(pixel intersections)
3000
2000
1000
0
-1000
50
100
150
200
250
300
350
400
450
500
550
600
650
700
750
Distance (µm)
HC NT
HC sh-Scr
HC sh-SLITRK2

Supplementary Figure S7: Axonal outgrowth in FUS-ALS versus FUS-iso motor neurons.

A. Representative images of axonal outgrowth in the distal compartment of FUS-iso and FUS-ALS MN visualized by immunocytochemical staining for TUBB3. Scale bar: 200 µm. **B.** Masks used for linear Sholl analysis of TUBB3 immunostaining with intersection lines at 50 µm intervals, starting from the microgroove exit. **C.** Quantifications of axonal outgrowth, measured as the number of pixel intersections with Sholl intersection lines every 50 µm, in FUS-iso and FUS-ALS MN. Data is presented as mean ± SD (*n*=2 biological replicates, *t*=3 technical replicates). Paired two-tailed T-test. **D.** Schematic overview of *SLITRK2* knockdown (KD) or overexpression (OE) in iPSC-derived MN cultures. (i) MN were treated with lentivirus for SLITRK2 KD or OE one day after plating in microfluidics devices (at D19 of differentiation) and axonal outgrowth is assessed 72h later (at D22). (ii) The shSLITRK2 construct, with sense sequence GCCACTGGGTATCACTACTTTCTCGAGAAAGTAGTGATAC CCAGTGGC, was inserted into the pLKO.1 lentiviral backbone under control of a human U6 promoter, and (iii) targets the proximal 3' UTR region of *SLITRK2*, thereby knocking down both isoforms. **E.** Representative images of axonal outgrowth in the distal compartment of NT, sh-Scr and sh-SLITRK2 treated HC MN at D22, visualized by immunocytochemical staining for TUBB3. Scale bar: 200 µm. **F.** Quantification of axonal outgrowth, measured as the number of pixel intersections with Sholl intersection lines every 50 µm, in NT, sh-Scr and sh-SLITRK2 treated HC MN at D22. Data are presented as mean ± SD (*n*=3, *t*=1). Kruskal-Wallis test. Source data are provided as a Source Data file.

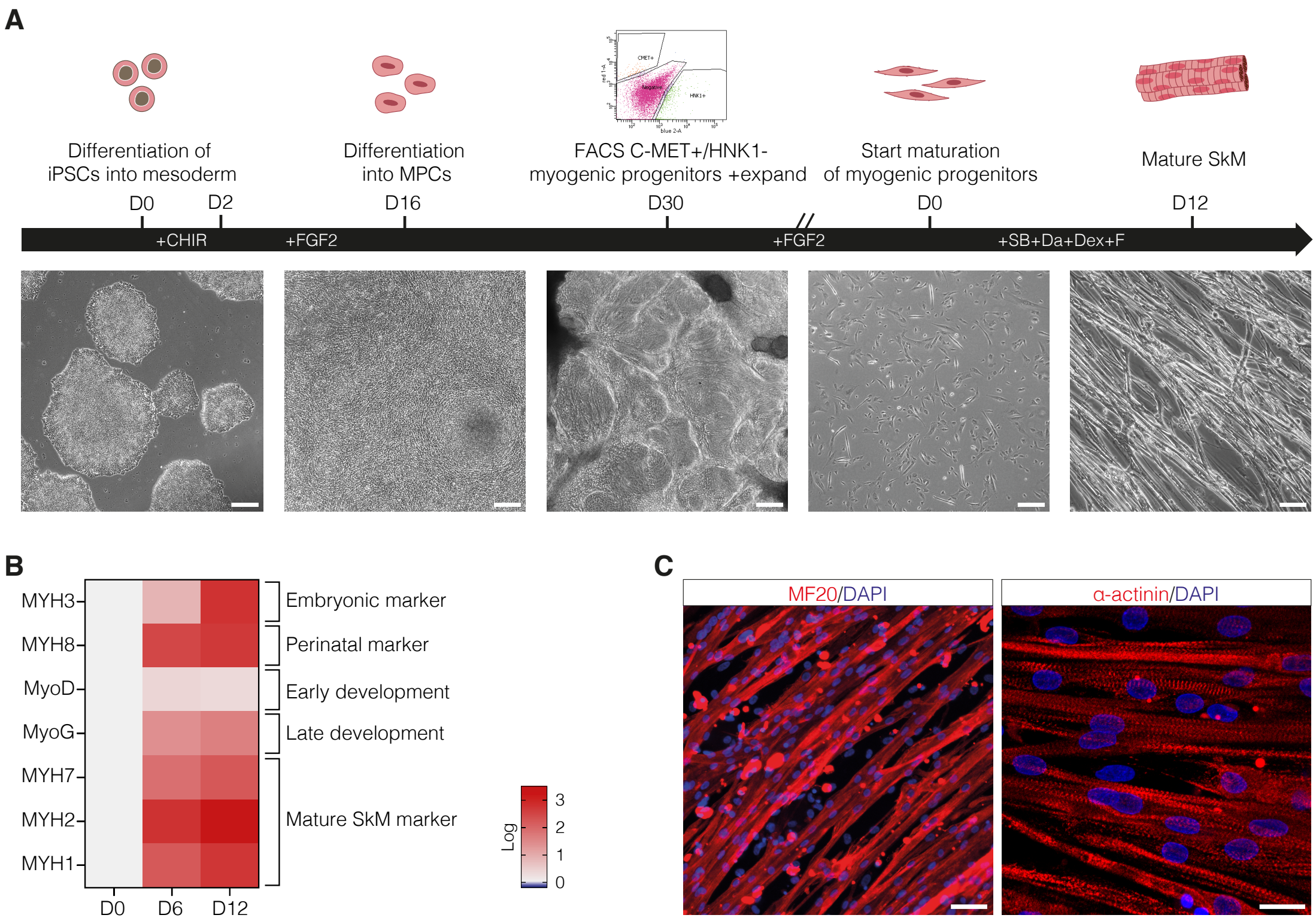
A
Differentiation of iPSCs into mesoderm
Differentiation into MPCs
FACS C-MET+/HNK1- myogenic progenitors +expand
Start maturation of myogenic progenitors
Mature SkM
D0
D2
D16
D30
D0
D12
+CHIR
+FGF2
+FGF2
+SB+Da+Dex+F
B
MYH3
MYH8
MyoD
MyoG
MYH7
MYH2
MYH1
D0
D6
D12
Embryonic marker
Perinatal marker
Early development
Late development
Mature SkM marker
Log
3
2
1
0
C
MF20/DAPI
α-actinin/DAPI

Supplementary Figure S8: Generation and characterization of iPSC-derived skeletal muscle cells. **A.** Schematic showing the differentiation of iPSCs into skeletal muscle cells including representative brightfield pictures at different time point. Scale bar D0: 200 µm, scale bar D30: 250 µm, scale bar D12 after maturation: 100 µm. **B.** mRNA expression levels of different developmental and mature (skeletal muscle) markers at different time points (n=1). **C.** Expression of skeletal muscle markers MF20 and α-actinin in iPSC-derived skeletal muscle cells on D12 after maturation. Scale bar left: 50 µm, right: 20 µm. Source data are provided as a Source Data file.

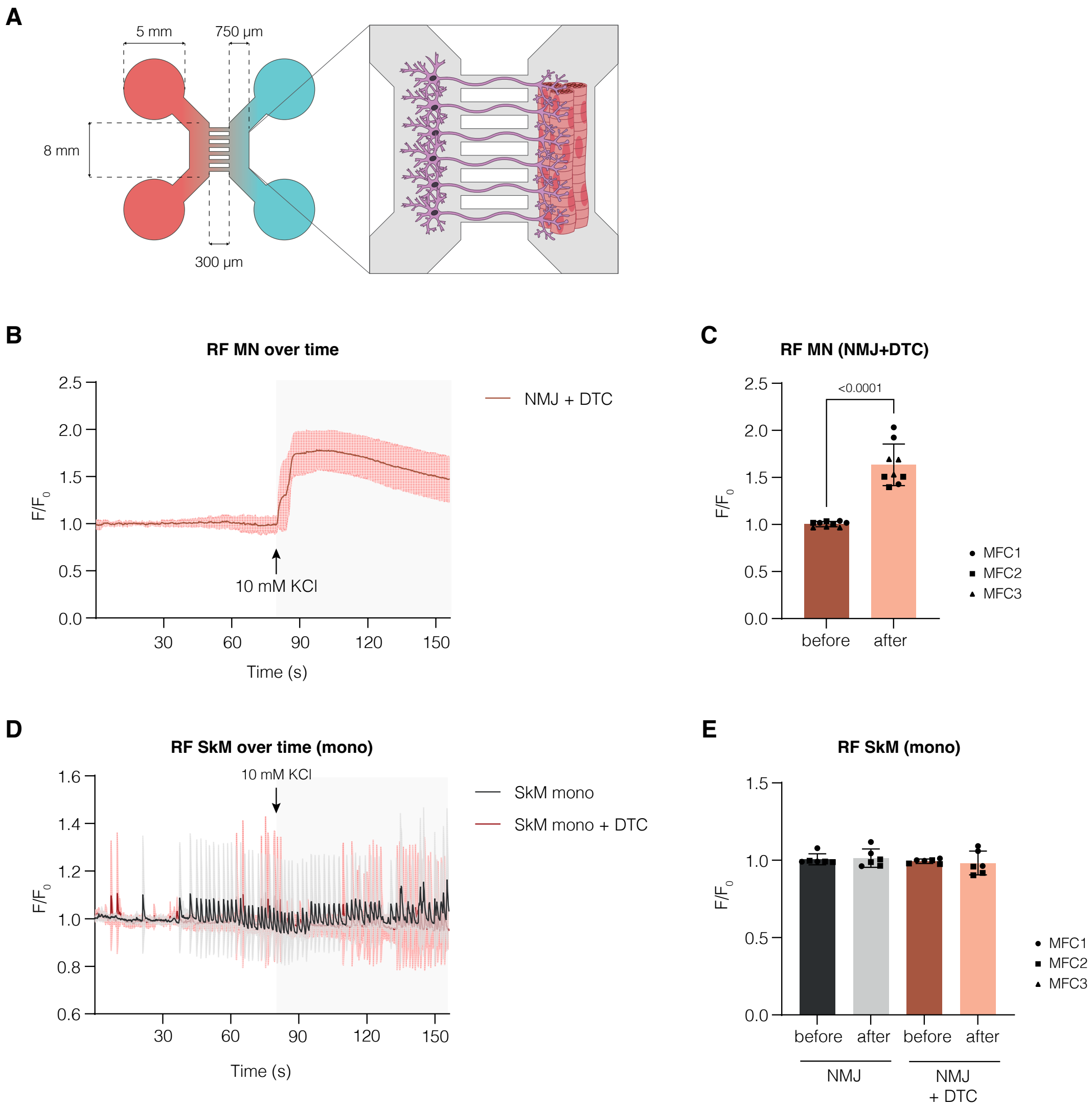
A
5 mm
750 µm
8 mm
300 µm
B
RF MN over time
2.5
2.0
1.5
1.0
0.5
0.0
F/F0
10 mM KCl
30
60
90
120
150
Time (s)
NMJ + DTC
C
RF MN (NMJ+DTC)
<0.0001
before
after
MFC1
MFC2
MFC3
D
RF SkM over time (mono)
1.6
1.4
1.2
1.0
0.8
0.6
10 mM KCl
Time (s)
SkM mono
SkM mono + DTC
E
RF SkM (mono)
1.5
1.0
0.5
0.0
before
after
before
after
NMJ
NMJ
+ DTC
MFC1
MFC2
MFC3

Supplementary Figure S9: DTC treatment of motor neuron and skeletal muscle monocultures. A. Schematic overview of compartmentalized microfluidic devices, including dimensions. **B.** Relative fluorescence of Ca^{2+} intensity in healthy MN, after 10 min treatment of the skeletal muscle with DTC, before and after (grey box) MN stimulation with 10mM KCl (arrow). **C.** Quantification of relative fluorescence in MN, after 10 min treatment of the skeletal muscle with DTC, before and after stimulation with 10mM KCl. **D.** Relative fluorescence of Ca^{2+} intensity in skeletal muscle monocultures before and after (grey box) stimulation with 10mM KCl (arrow) in the MN compartment, without and with 10 min treatment of the skeletal muscle with DTC. **E.** Quantification of relative fluorescence in skeletal muscle monocultures before and after stimulation in the MN compartment with 10mM KCl, and without or with treatment with DTC. (p = 0.7412). *All data in B-E is presented as mean ± SD of 2 cultures (same biological donor for MN and myotubes), 3 ROIs/culture. B: Paired two-tailed T-test, D: One-way ANOVA. Source data are provided as a Source Data file.

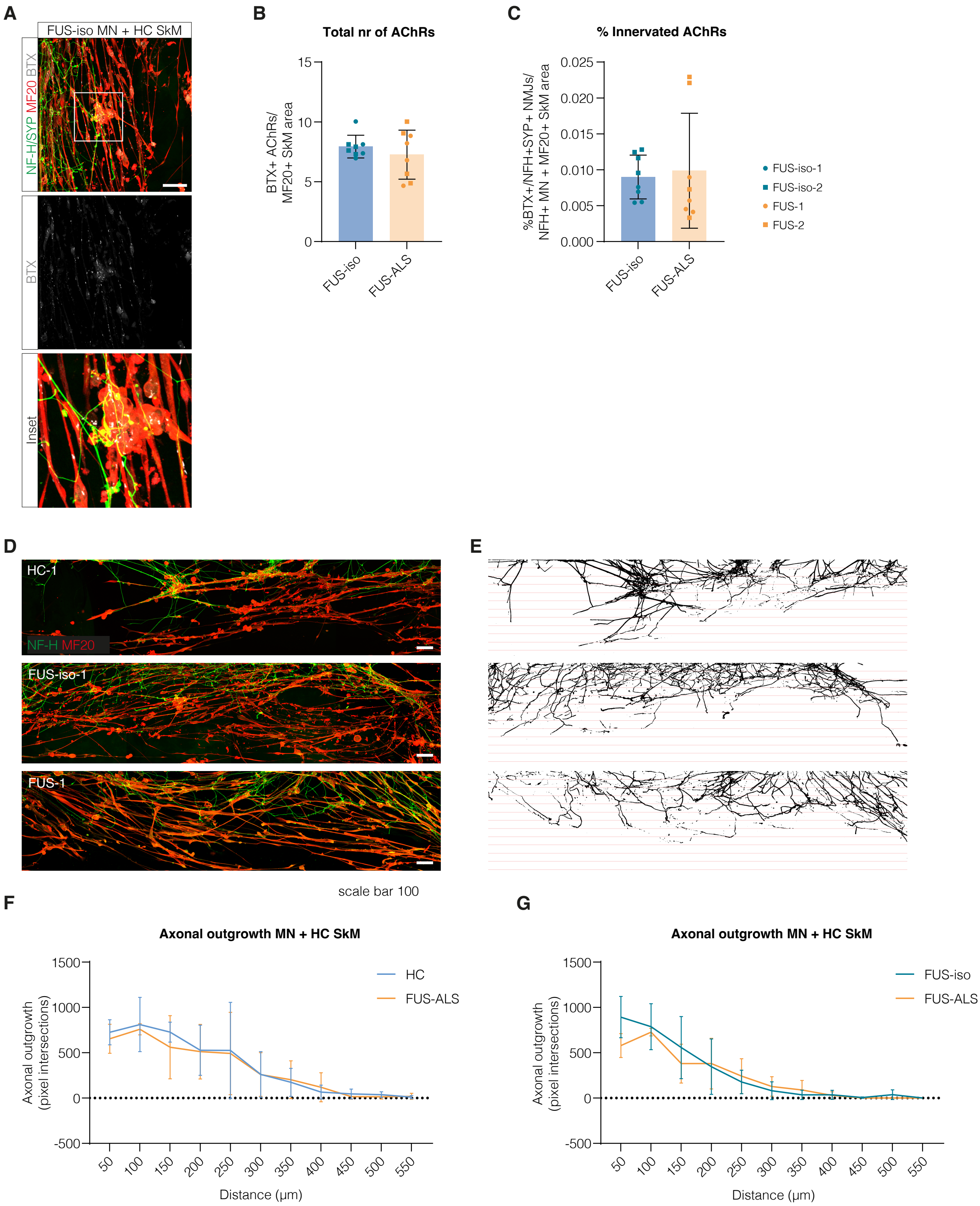

A
FUS-iso MN + HC SkM
NF-H/SYP MF20 BTX
BTX
Inset
B
Total nr of AChRs
BTX+ AChRs/
MF20+ SkM area
15
10
5
0
FUS-iso
FUS-ALS
C
% Innervated AChRs
%BTX+/NFH+SYP+ NMJs/
NFH+ MN + MF20+ SkM area
0.025
0.020
0.015
0.010
0.005
0.000
FUS-iso
FUS-ALS
FUS-iso-1
FUS-iso-2
FUS-1
FUS-2
D
HC-1
NF-H MF20
FUS-iso-1
FUS-1
E
scale bar 100
F
Axonal outgrowth MN + HC SkM
Axonal outgrowth
(pixel intersections)
1500
1000
500
0
-500
50
100
150
200
250
300
350
400
450
500
550
Distance (µm)
HC
FUS-ALS
G
Axonal outgrowth MN + HC SkM
Axonal outgrowth
(pixel intersections)
Distance (µm)
FUS-iso
FUS-ALS

Supplementary Figure S10: NMJ characterization of FUS-iso co-cultures and analysis of axonal outgrowth in iNMJ-on-a-chip model.

A. Immunofluorescence image of co-culture containing FUS-iso MN with HC myotubes. MN are positive for NF-H and SYP (green), myotubes are positive MF20 (magenta) and AChRs are visualized with BTX (cyan). Inset showing the formation of NMJs as defined by BTX and NF-H-SYP colocalization. Scale bar: 100 µm. **B.** Quantification of total nr of AChRs on HC myotubes co-cultured FUS-iso MN normalized to the total % area of MF20. Two-tailed Mann-Whitney test ($p = 0.7002$). **C.** Quantification of innervated NMJs, defined as BTX and NF-H/SYP colocalizations in FUS-iso MN co-cultures with HC myotubes, normalized to the sum of the total % area of NF-H/SYP and MF20. Data is presented as mean ± SD (n=2 biological replicates, t=4 technical replicates). Two-tailed Mann-Whitney test ($p = 0.5054$). **D.** Representative images of axonal outgrowth in the muscle compartment of HC, FUS-iso and FUS-ALS MN co-cultured with HC myotubes, visualized by immunocytochemical staining for NF-H (MN) and MF20 (myotubes). Scale bar: 200 µm. **E.** Masks used for linear Sholl analysis of NF-H immunostaining with intersection lines at 50 µm intervals, starting from the microgroove exit. **F.** Quantifications of axonal outgrowth, measured as the number of pixel intersections with Sholl intersection lines every 50 µm, in HC and FUS-ALS MN, and **G.** FUS-iso and FUS-ALS MN co-cultured with HC myotubes. Data is presented as mean ± SD (F: n=3, t=1; G: n=2, t=1). F: Paired two-tailed T-test ($p = 0.1472$), G: Two-tailed Mann-Whitney test ($p = 0.6063$). Source data are provided as a Source Data file.

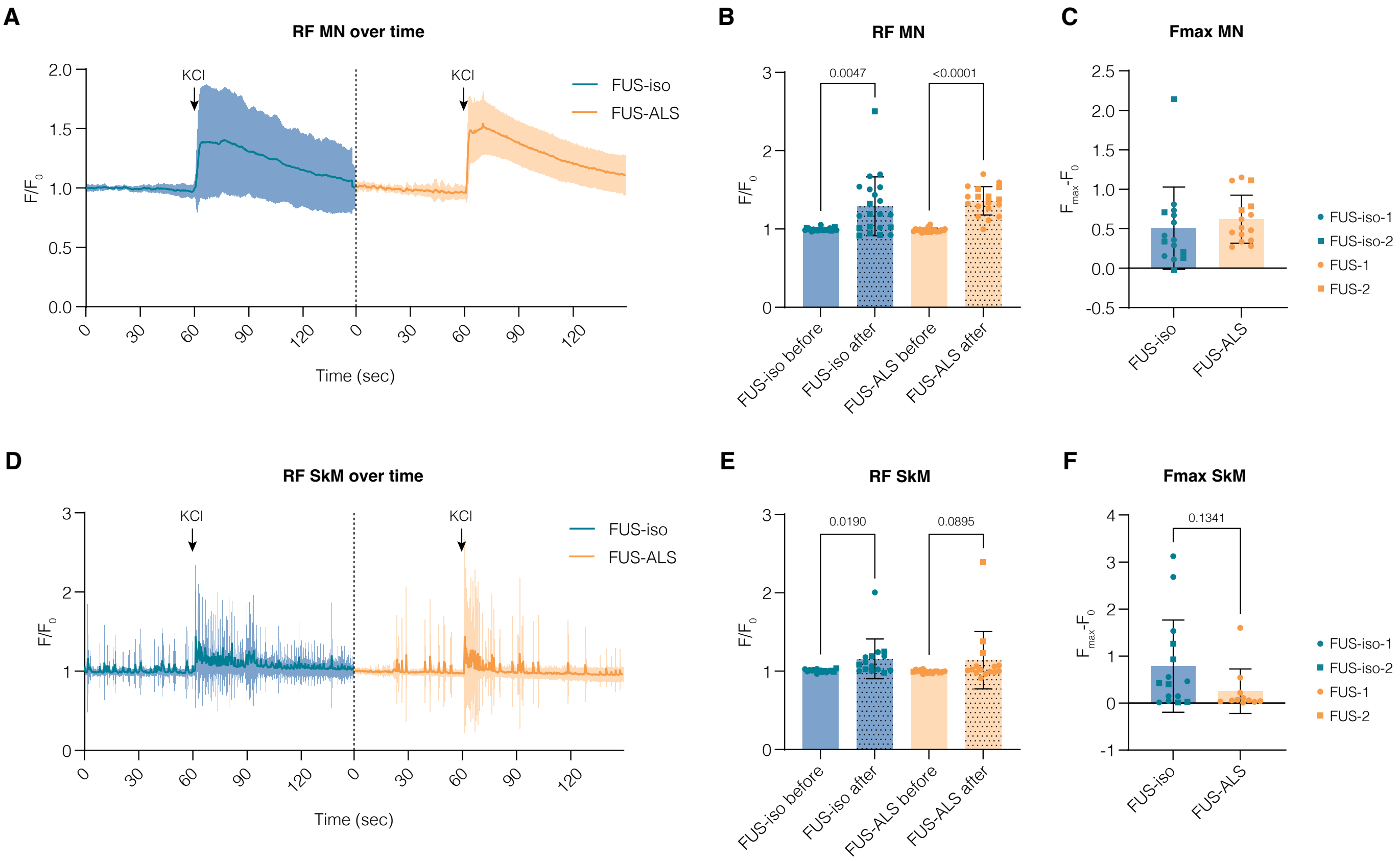
A
RF MN over time
KCl
KCl
FUS-iso
FUS-ALS
F/F0
Time (sec)
B
RF MN
0.0047
<0.0001
FUS-iso before
FUS-iso after
FUS-ALS before
FUS-ALS after
C
Fmax MN
Fmax-F0
FUS-iso
FUS-ALS
FUS-iso-1
FUS-iso-2
FUS-1
FUS-2
D
RF SkM over time
E
RF SkM
0.0190
0.0895
F
Fmax SkM
0.1341

Supplementary Figure S11: Neuromuscular transmission in FUS-ALS versus FUS-iso co- cultures. **A.** Relative fluorescence (F/F_0) of Ca^{2+} intensity over time in FUS-iso and FUS-ALS MN co-cultured with HC myotubes, before and after MN stimulation with 10 mM KCl (arrow). **B.** Quantification of the relative fluorescence (F/F_0) in FUS-iso and FUS-ALS MN before and after MN stimulation. Data is presented as mean ± SD. Biological (*n*) and technical *(t*) replicates per group: FUS-iso (*n*=2, *t*=12,9), FUS-ALS (*n*=2, *t*=12,6). Kruskal-Wallis test. **C.** Quantification of *F*max (F_{max}-F_0) in FUS-iso and FUS-ALS MN co-cultured with HC myotubes. Data is presented as mean ± SD. FUS-iso (*n*=2, *t*=9,6), FUS-ALS (*n*=2, *t*=12,3). Unpaired two-tailed T-test (p = 0.4750). **D.** Relative fluorescence (F/F_0) of Ca^{2+} intensity over time in HC myotubes co-cultured with FUS-iso and FUS-ALS MN, before and after MN stimulation with 10 mM KCl (arrow). **E.** Quantification of the relative fluorescence (F/F_0) in HC myotubes co-cultured with FUS-iso and FUS-ALS MN. Data is presented as mean ± SD. FUS-iso (*n*=2, *t*=9,6), FUS-ALS (*n*=2, *t*=12,3). Kruskal-Wallis test. **F.** Quantification of Fmax (F_{max}-F_0) in HC myotubes co-cultured with FUS-iso and FUS-ALS MN. Data is presented as mean ± SD. FUS-iso (*n*=2, *t*=9,6), FUS-ALS (*n*=1, *t*=11). Two-tailed Mann-Whitney test. Source data are provided as a Source Data file.

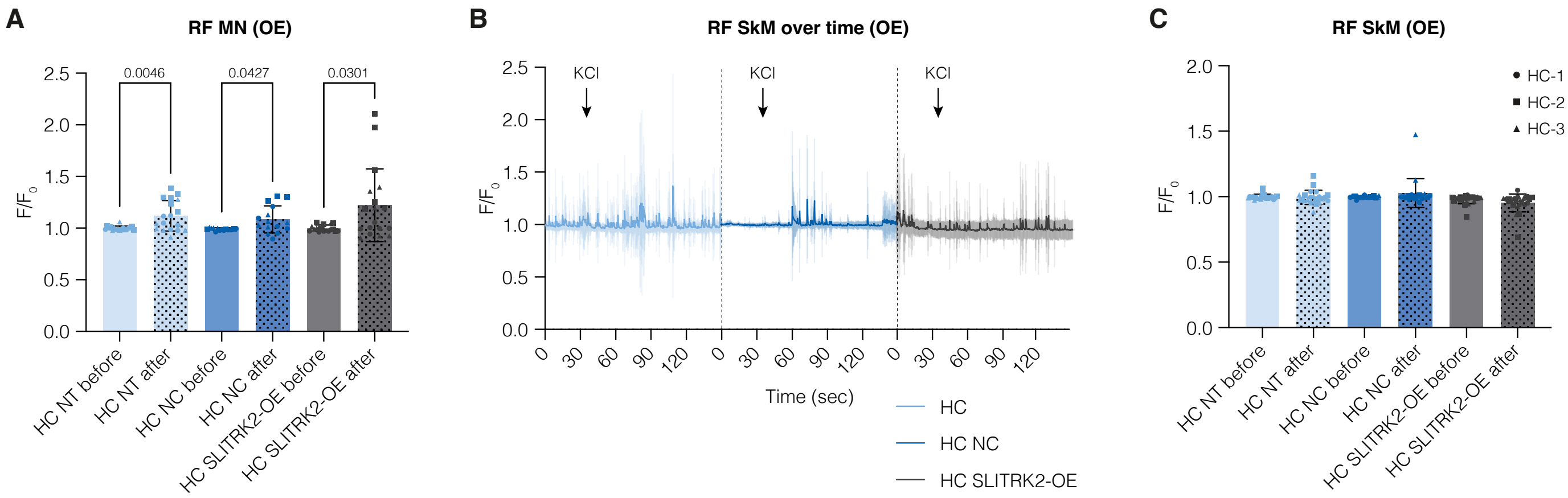
A
RF MN (OE)
0.0046
0.0427
0.0301
F/F0
HC NT before
HC NT after
HC NC before
HC NC after
HC SLITRK2-OE before
HC SLITRK2-OE after
B
RF SkM over time (OE)
KCl
KCl
KCl
Time (sec)
HC
HC NC
HC SLITRK2-OE
C
RF SkM (OE)
HC-1
HC-2
HC-3

Supplementary Figure S12: Effect of SLITRK2 overexpression in HC MN on axonal neuromuscular transmission.

A. Quantification of the relative fluorescence (F/F_0) in NT, NC and SLITRK2-OE treated HC MN before and after MN stimulation. Data is presented as mean ± SD. Biological (*n*) and technical (*t*) replicates per group: HC (*n*=3, *t*=6,6,6), HC NC (*n*=3, *t*=6,3,6), HC SLITRK2 OE (*n*=3, *t*=6,6,6). Mixed-effect analysis. **B.** Relative fluorescence (F/F_0) of Ca^{2+} intensity over time in HC myotubes co-cultured with in NT, NC and SLITRK2-OE treated HC MN, before and after MN stimulation with 10 mM KCl (arrow). Data is presented as mean ± SD. **C.** Quantification of the relative fluorescence (F/F_0) in HC myotubes co-cultured with in NT, NC and SLITRK2-OE treated HC MN. Data is presented as mean ± SD. HC (*n*=3, *t*=7,8,8), HC NC (*n*=3, *t*=8,4,8), HC SLITRK2 OE (*n*=3, *t*=8,8,8). Kruskal-Wallis test. Source data are provided as a Source Data file.

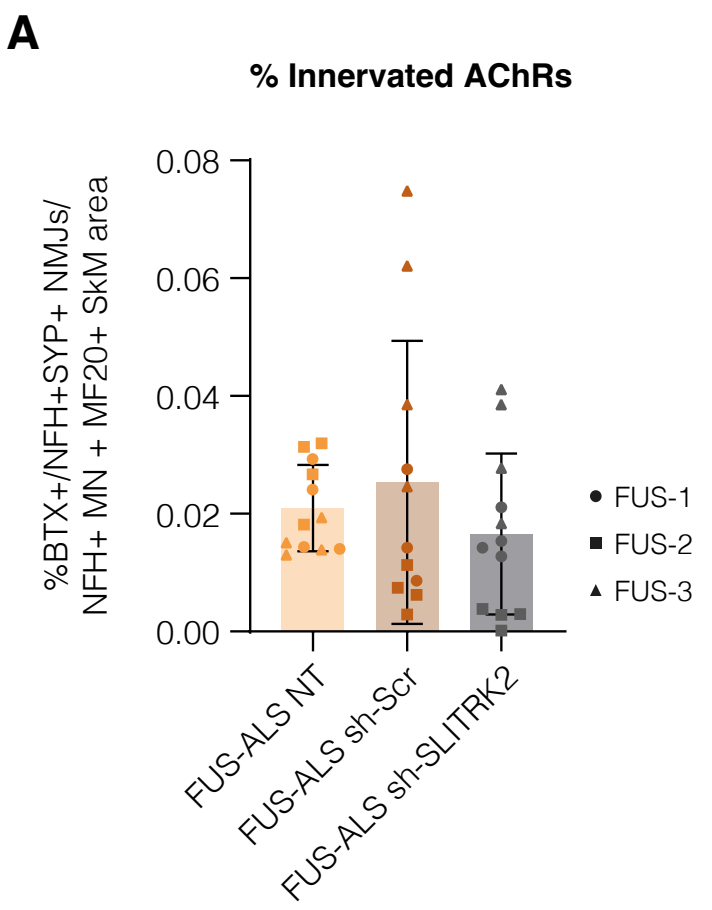
A
% Innervated AChRs
%BTX+/NFH+SYP+ NMJs/
NFH+ MN + MF20+ SkM area
0.08
0.06
0.04
0.02
0.00
FUS-1
FUS-2
FUS-3
FUS-ALS NT
FUS-ALS sh-Scr
FUS-ALS sh-SLITRK2

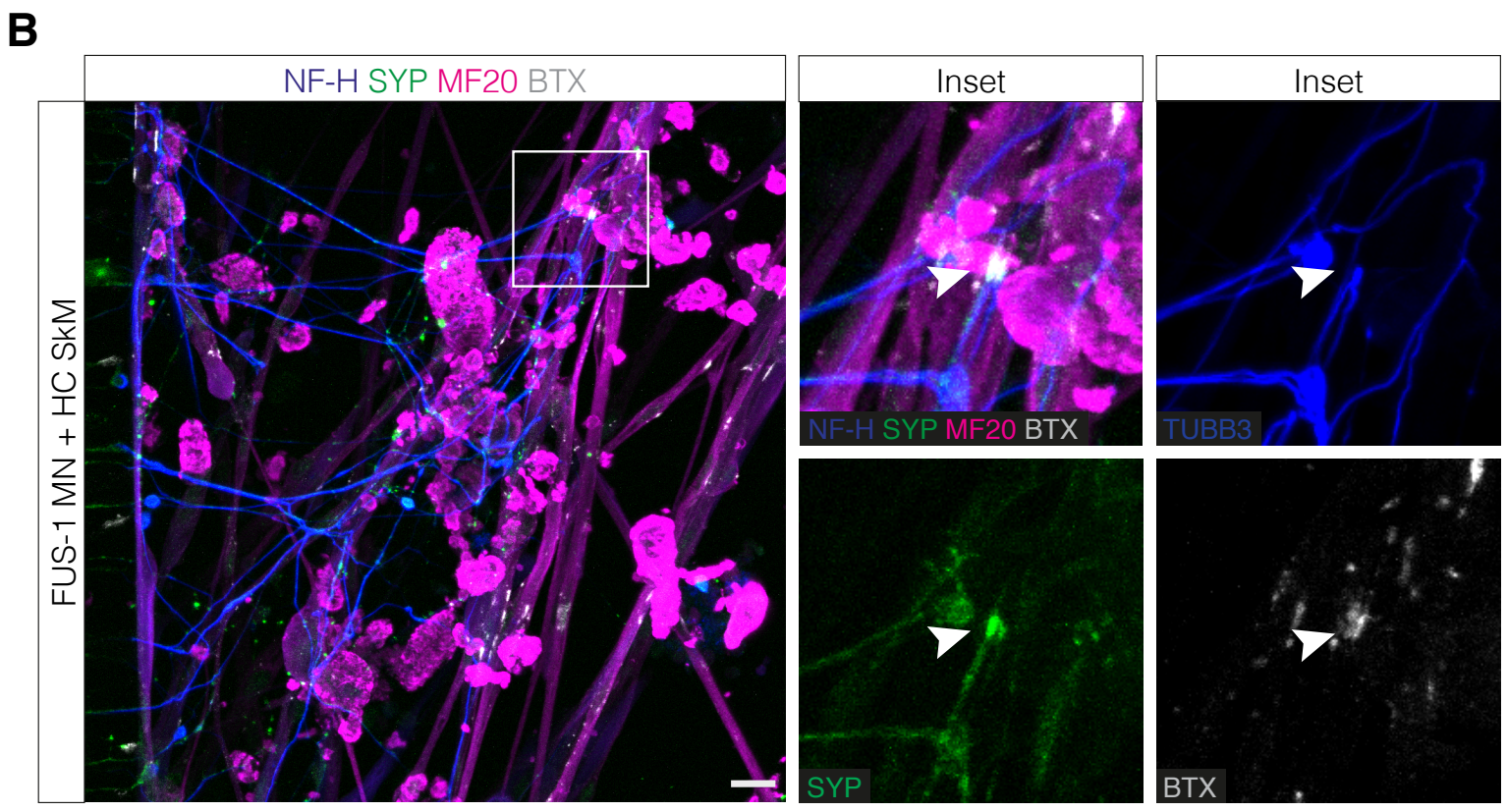
B
NF-H SYP MF20 BTX
Inset
Inset
FUS-1 MN + HC SkM
NF-H SYP MF20 BTX
TUBB3
SYP
BTX

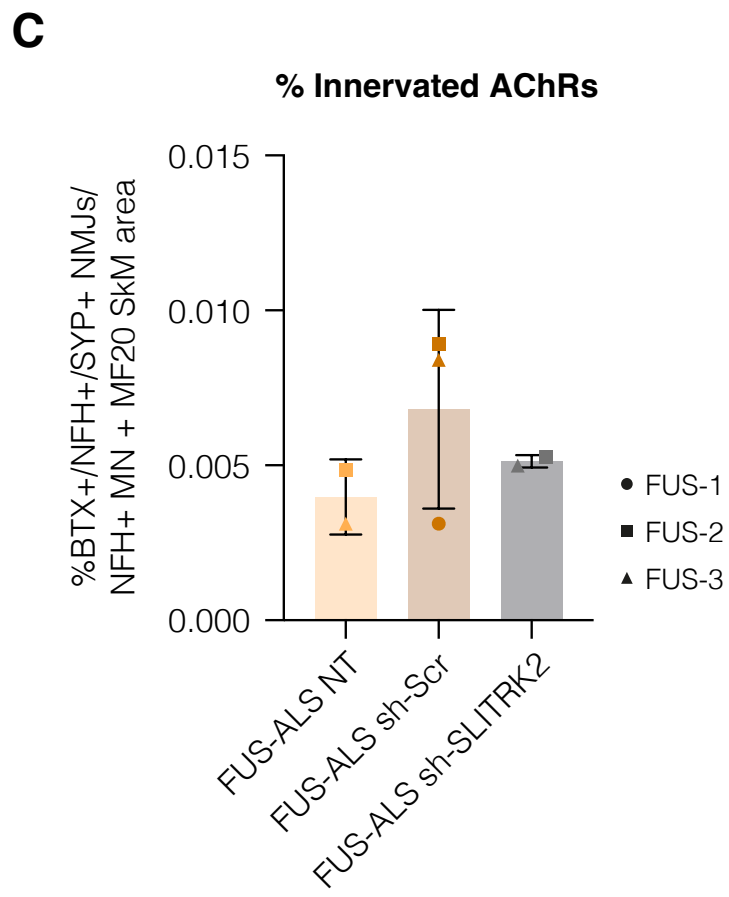
C
% Innervated AChRs
%BTX+/NFH+/SYP+ NMJs/
NFH+ MN + MF20 SkM area
0.015
0.010
0.005
0.000
FUS-1
FUS-2
FUS-3
FUS-ALS NT
FUS-ALS sh-Scr
FUS-ALS sh-SLITRK2

Supplementary Figure S13: Number of innervated NMJs in sh-SLITRK2 treated FUS-ALS co-cultures.

A. Quantification of innervated NMJs, defined as BTX and NF-H/SYP colocalizations in NT, sh-Scr treated of sh-SLITRK2 treated FUS-ALS MN co-cultures with HC myotubes, normalized to the sum of the total % area of NF-H/SYP and MF20. Data is presented as mean ± SD. Biological (*n*) and technical (*t*) replicates per group: FUS-ALS NT (*n*=3, *t*=4,4,4), FUS-ALS scr (*n*=3, *t*=3,4,4), FUS-ALS shSLITRK2 (*n*=3, *t*=4,4,4). Mixed effect analysis (p = 0.1804). **B.** Representative immunofluorescence images of FUS-ALS MN and HC myotube co-cultures. MNs are positive for NF-H (blue) and SYP (green), myotubes are positive for MF20 (magenta), and AChRs are visualized with BTX (grey). The inset displays the formation of NMJs, defined by the colocalization of BTX, NF-H and SYP. Scale bar: 100 µm. **C.** Quantification of innervated NMJs as in (A), confirming that similar results are obtained when assessing colocalization across separate NF-H and SYP imaging channels. Data is presented as mean ± SD. FUS-ALS NT (*n*=2, *t*=1), FUS-ALS scr (*n*=3, *t*=1), FUS-ALS shSLITRK2 (*n*=2, *t*=1). Kruskal-Wallis test (p = 0.4952). Source data are provided as a Source Data file.

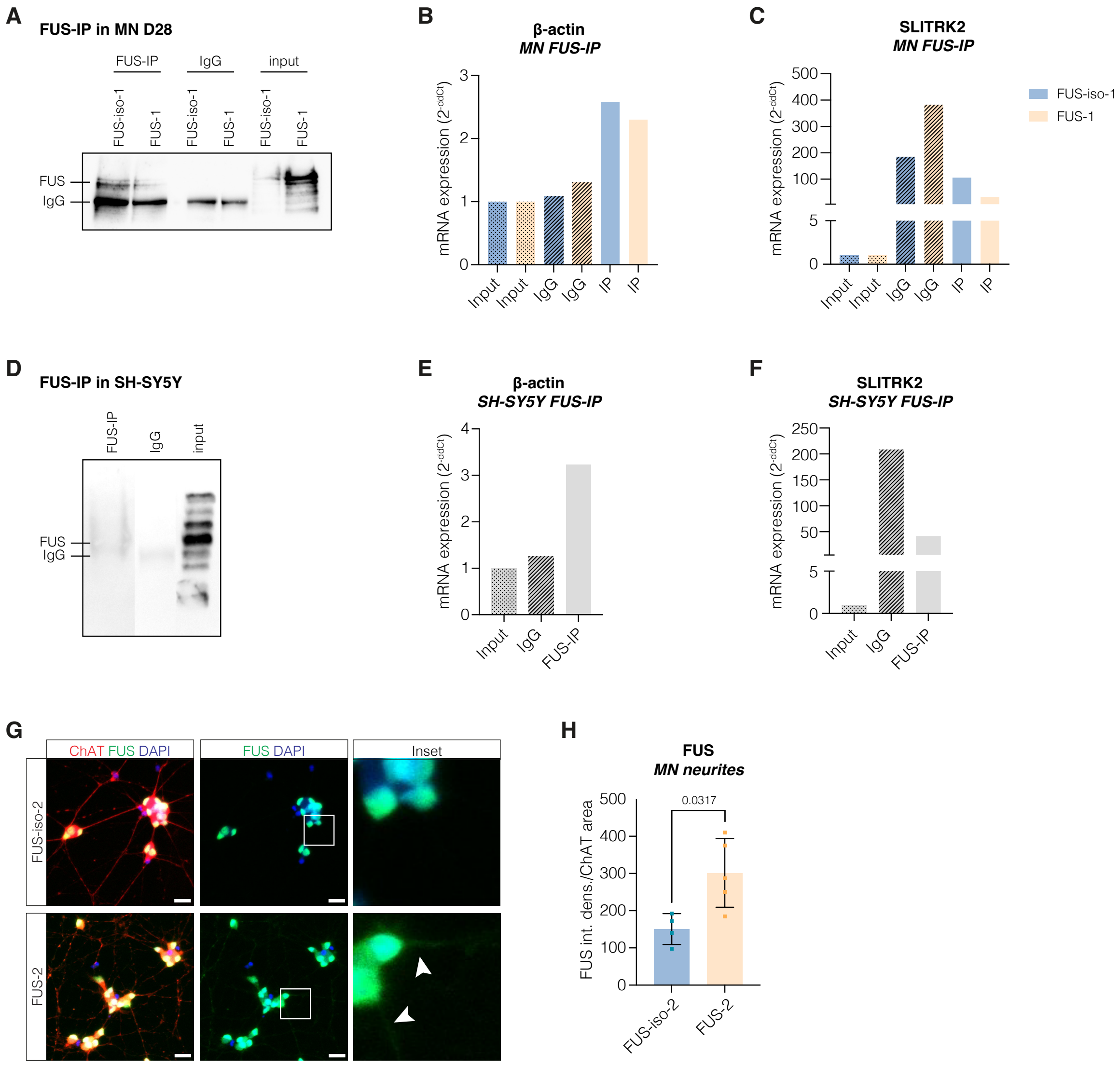

A
FUS-IP in MN D28
FUS-IP
IgG
input
FUS-iso-1
FUS-1
FUS
IgG
B
β-actin
MN FUS-IP
mRNA expression (2^-ddCt)
Input
IgG
IP
C
SLITRK2
MN FUS-IP
FUS-iso-1
FUS-1
D
FUS-IP in SH-SY5Y
E
β-actin
SH-SY5Y FUS-IP
F
SLITRK2
SH-SY5Y FUS-IP
G
ChAT FUS DAPI
FUS DAPI
Inset
FUS-iso-2
FUS-2
H
FUS
MN neurites
0.0317
FUS int. dens./ChAT area

Supplementary Figure S14: Analysis of FUS-SLITRK2 interactions in FUS-ALS MN and SH-SY5Y cells.

A. Western blot analysis of FUS immunoprecipitation (IP) in MN at D28. Samples were stained for FUS. Shown are input, IgG and IP fractions for both FUS-iso and FUS-ALS MN. **B.** mRNA expression levels (2^{-ddCT}) of *β-actin* and **C.** *SLITRK2* in input, IgG, and FUS-IP fractions from FUS-iso and FUS-ALS MNs. Data is presented as mean (*n*=1 biological replicate, *t*=1 technical replicate). **D-F.** Same experimental setup as A-C, performed in SH-SY5Y cells. **G.** Representative immunofluorescence staining for FUS in FUS-ALS and FUS-iso MN at D28, co-stained with ChAT and DAPI. Arrows indicate FUS mislocalization in neurites of FUS-ALS MNs. Scale bar: 20 µm. **H.** Quantification of FUS integrated density normalized to ChAT area in the neurites of FUS-iso and FUS-ALS MNs. Data is presented as mean ± SD. FUS-iso (*n*=1, *t*=4), FUS-ALS (*n*=1, *t*=5). Two-tailed Mann-Whitney test. Source data are provided as a Source Data file.

Supplementary Table S1: Genetic and clinical information of iPSC lines used in this study.

iPSC ID	Genotype	Sex	Age of biopsy (years)	Reference
HC-1	Healthy control	Female	60	[113]
HC-2	Healthy control	Male	63	[114]
HC-3	Healthy control	Male	62	[114]
HC-4	Healthy control	Male	62	[115]
HC-5	Healthy control	Male	61	[116]
HC-6	Healthy control	Female	60	[115]
HC-7	Healthy control	Male	72	[117]
FUS-1	FUS R521H	Male	71	[81]
FUS-iso-1	Isogenic ctrl FUS-1	Male	71	[81]
FUS-2	FUS P525L	Male	17	[81]
FUS-iso-2	Isogenic ctrl FUS-2	Male	17	[81]
FUS-3	FUS R521C	Female	32	N/A
Knock-in WT	WT knockin	Male	8	[70]
P525L knock-in	FUS P525L	Male	8	[70]

iPSC = induced pluripotent stem cell; ID = identity; iso = isogenic, N/A = not applicable.

Supplementary Table S2: Primers used in this study.

Gene	5' – 3' forward primer	5' – '3 Reverse Primer
FUS_outer	GGAACATAGGGGAATGGGA	TAGCTGGGCAAATTTAGGC
FUS	CCGGGGTGGTGGGGACAGAGGTGG	-
GAPDH	TGCACCACCAACTGCTTAGC	GGCATGGACTGTGGTCATGA
B-actin	GTGGACATCCGCAAAGACCT	TCTGCATCCTGTCGGCAAT
RPS2	CGCACCAGGTTCAAGGCAT	AGCACTTAACACCCAGACCG
NANOG	GCCTGTGATTTGTGGGCCTGA	GTGGAAGAATCAGGGCTGTCCTG
OCT4	GGAGAAGCTGGAGCAAAACC	TGGCTGAATACCTTCCCAAA
SOX2	CGAGGGAAATGGGAGGGGTGC	TGCAGCTGTCATTTGCTGTGGGT
PAX6	AGTTCTTCGCAACCTGGCTA	ATTCTCTCCCCCTCCTTCCT
Ki67	AGAAGAAGTGGTGCTTCGGAA	AGTTTGCGTGGCCTGTACTAA
OLIG2	CCAGAGCCCGATGACCTTTTT	CACTGCCTCCTAGCTTGTCC
ISL1	AAGGACAAGAAGCGAAGCAT	TTCCTGTCATCCCCTGGATA
Hb9	TGCCTAAGATGCCCGACTT	TTGAGCTTGAACTGGTGCTC
ChAT	GACGTCTGACGGGAGGAG	TCAATCATGTCCAGCGAGTC
MAP2	TGCCTCAGAACAGACTGTCA	GGCTCTTGGTTACTCCGTCA
NeuN	GACGCAATGGTTCAGCCTTTT	GCGTACTTCCGTAGAGTGTCAG
Synapsin	AGTTCTTCGGAATGGGGTGAA	CAAACTGCGGTAGTCTCCGTT
VAChT	CTGCTAGTGAACCCCTTGAGC	CAGGACTGTAGAGGCGAACAT
SMI-32	GCAGTCCGAGGAGTGGTTC	CGCATAGCGTCTGTGTTCA
SLITRK2	CCCCCGGGAAATCAGGTTTG	GTCTGTAAGATCCCGGCCAC

FUS = Fused in Sarcoma; HPRT1 = Hypoxanthine Phosphoribosyltransferase 1; TBP = TATA-binding protein; GAPDH = Glyceraldehyde 3-phosphate dehydrogenase; RPS2 = Ribosomal Protein S2; OCT4 = POU Class 5 Homeobox 1 (POU51); SOX2 = SRY-Box Transcription Factor 2; PAX6 = Paired box protein Pax-6; Ki67 = Kiel 67; OLIG2 = Oligodendrocyte transcription factor; ISL1 = Islet 1; ChAT = Choline Acetyltransferase; MAP2 = Microtubule Associated Protein 2; NeuN = Neuronal Nuclei; VAChT = Vesicular Acetylcholine Transporter; SMI-32 = Small Integral Membrane Protein 32; SLITRK2 = SLIT And NTRK Like Family Member 2; MYH = Myosin Heavy Chain; MyoD = Myogenic differentiation 1; MyoG = Myogenin.

Supplementary Table S3: Medium composition for iPSC-derived lower motor neuron generation.

Medium	Company, catalog number	Concentration
**NDM Basal medium**		
DMEM/F-12	Gibco, 11320-074	1:2
Neurobasal	Gibco, 21103-049	1:2
Sodium pyruvate	Gibco, 11360-070	1 mM
Penicillin-Streptomycin	Gibco, 15140-122	100 units/mL;100 µg/mL
L-Glutamine	Gibco, 25030-024	2 mM
MEM NEAA	Gibco, 11140-035	1X
N-2 supplement	Gibco, 17502-001	1X
B-27 supplement	Gibco, 17504-001	1X
**NDM1**		
NDM Basal Medium		1X
Ascorbic Acid	Sigma-Aldrich, A4403	200 µM
LDN-193189	Miltenyi Biotec. 130-103-925	200 nM
CHIR99021	Sigma-Aldrich, SML1046	3 µM
SB-431542	Axon Biochemicals, AXON1661	10 µM
**NDM2**		
NDM Basal Medium		1X
Ascorbic Acid	Sigma-Aldrich, A4403	200 µM
LDN-193189	Miltenyi Biotec. 130-103-925	200 nM
CHIR99021	Sigma-Aldrich, SML1046	1 µM
SB-431542	Axon Biochemicals, AXON1661	10 µM
Retinoic acid	Sigma-Aldrich, R2625	200 nM
Purmorphamine	Stemgent, 04-0009	500 nM
cAMP	Sigma-Aldrich, A6885	0,5 µM
**NDM3**		
NDM Basal Medium		1X
Ascorbic Acid	Sigma-Aldrich, A4403	200 µM
Retinoic acid	Sigma-Aldrich, R2625	200 nM
Purmorphamine	Stemgent, 04-0009	500 nM
cAMP	Sigma-Aldrich, A6885	0,5 µM
**NDM4**		
NDM Basal Medium		1X
Ascorbic Acid	Sigma-Aldrich, A4403	200 µM
BrainFast Neuronal Maturation Supplement	BrainXell, BX-2000	1X
BDNF	Stemcell Technologies, 78005	10 ng/ml
GDNF	Stemcell Technologies, 78058	10 ng/ml
CNTF	Stemcell Technologies, 78010	10 ng/ml

NDM = neuronal differentiation medium; DMEM = Dulbecco's Modified Eagle Medium; MEM

= Minimum Essential Medium; NEAA = Non-Essential Amino Acids; cAMP = cyclic adenosine monophosphate; BNDF = brain-derived neurotrophic factor; GNDF = glial cell line-derived neurotrophic factor; CNTF = Ciliary neurotrophic factor.

Supplementary Table S4: Antibodies used in this study.

Antibody	Company, catalog number	Concentration
**Primary antibodies**		
CXCR4 (Rb)	Thermofisher, PA3-305	1:1000
SOX17 (Rb)	Thermofisher, MA5-24885	1:100
Brachyury (Rb)	Thermofisher, PA5-23405	1:500
PAX6 (Rb)	Thermofisher, 42-6600	1:100
Nestin (Ms)	Thermosfisher, MA1-110	1:100
TUBB3 (Rb)	Biolegend, 802001	1:500
NEFH (Rb)	Sigma-Aldrich, N4142	1:500
NEFH (Ch)	Millipore, AB5539	1:500
MAP2 (Ch)	Neuromics, CH22103	1:1000
ISL1 (Ms)	DSHB, 40.3A4	1:50
ChAT (Gt)	R&D Systems, AF3447	1:100
FUS (Rb)	Bethyl Laboratories, A300-293A	1:500
SLITRK2 (Rb)	Thermofisher, BS-11955R	1-:100-1:500
Synaptohysin (Gp)	Synaptic Systems, 101-004	1:500
MF20 (Ms)	DSHB, AB_2147781	1:50
α-actinin (Ms)	Sigma-Aldrich, A7811	1:100
α-BTX Alexa Fluor 647 Conjugate	Thermofisher, B35450	1:1000
**Secondary antibodies**		
Alexa fluor 405 Donkey anti Mouse	Abcam, ab175658	1:500
Alexa fluor 488 Donkey anti Rabbit	Lifetechnologies, A21206	1:500
Alexa fluor 488 Goat anti Rabbit	Lifetechnologies, A11034	1:500
Alexa fluor 488 Goat anti Guinea pig	Thermofisher, A-11073	1:500
Alexa fluor 568 Donkey anti Mouse	Lifetechnologies, A10037	1:500
Alexa fluor 568 Goat anti Mouse	Lifetechnologies, A11004	1:500
Alexa fluor 568 Donkey anti Goat	Abcam, ab175474	1:500
Alexa fluor 647 Donkey anti Chicken	Jackson ImmunoResearch, 703-605-155	1:500

Rb = Rabbit; Ch = Chicken; Ms = Mouse; Gt = Goat; Gp = Guinea Pig; CXCR4 = C-X-C chemokine receptor type 4; SOX17; SRY-Box Transcription Factor 17; PAX6 = Paired Box 6; TUBB3 =Beta-tubulin 3; NEFH = Neurofilament Heavy Chain; Map2 = Microtubule Associated Protein 2; Isl1 = Islet 1; ChAT = Choline Acetyltransferase; FUS = Fused in Sarcoma; SLITRK2 = SLIT And NTRK Like Family Member 2; BTX = Alpha-Bungarotoxin.

Supplementary Table S5: Medium composition for differentiation of SH-SY5Y cells.

SH-SY5Y differentiation medium	Company, catalog number	Concentration
Neurobasal	Life Technologies, 21103049	
Penicillin-Streptomycin	Thermo Fisher, 15140122	10000 units/mL
L-Glutamine	Life Technologies, 25030024	200 mM
B-27 supplement	Thermo Fisher, 17504-001	50X
cAMP	Sigma-Aldrich, A6885-1G	0.5 µM
Retinoic Acid	Sigma Alrich, R2625-50MG	10 µM
Human Recombinant BDNF	STEMCELL Technologies, 78005.1	10 ng/mL

cAMP = cyclic adenosine monophosphate; BNDF = brain-derived neurotrophic factor.

Supplementary Table S6: IP Lysis buffer composition.

IP Lysis buffer	Company, catalog number	Concentration
RNase free H2O		
NaCl	Merck, 1.064404.1000	300 mM
MgCl2	Merck, 1.05833.1000	5 mM
NP-40	Sigma, 74385-1L	0.1%
Tris-HCl (pH: 7.5)	Roche, 10708976001<br>Merck, 1.00317.2500	50 mM
RNAsin (add fresh)	Invitrogen, AM2696	100 U/mL
Complete mini (add fresh)	Roche, 11 836 170 001	1 tablet/10mL lysis buffer
tRNA (add fresh)	Sigma, R-6750	1 µg/mL

Supplementary Table S7: Medium composition for iPSC-derived skeletal muscle generation.

Medium	Company, catalog number	Concentration
**Myogenic differentiation medium (MDM)**		
DMEM/F-12	Gibco, 11320-074	1X
Penicillin-Streptomycin	Gibco, 15140-122	100 units/mL;100 µg/mL
L-Glutamine	Gibco, 25030-024	2 mM
ITS-X	Gibco, 51500-056	1X
**Myogenic proliferation medium (MPM)**		
DMEM high glucose	Gibco, 31966-047	1X
Penicillin-Streptomycin	Gibco, 15140-122	100 units/mL;100 µg/mL
L-Glutamine	Gibco, 25030-024	2 mM
FBS	Sigma-Aldrich, F7524	1:10
**Myogenic maturation medium (MMM)**		
DMEM high glucose	Gibco, 31966-047	1X
Penicillin-Streptomycin	Gibco, 15140-122	100 units/mL;100 µg/mL
L-Glutamine	Gibco, 25030-024	2 mM
ITS-X	Gibco, 51500-056	1X
Knock-out Serum Replacement	Gibco, 10828-028	1:100

DMEM = Dulbecco's Modified Eagle Medium; ITS-X = Insulin-Transferrin-Selenium-Ethanolamine; FBS = fetal bovine serum.

Supplementary Table S8: Oligonucleotide sequences for SLITRK2 shRNA knock down.

	**Oligonucleotide sequence**
pLKO.1_hSLITRK2_6_s	CCGGTGCCACTGGGTATCACTACTTTCTCGAGAAAGTAGTGATACCCAGTGGCTTTTTG
pLKO.1_hSLITRK2_6_as	AATTCAAAAAGCCACTGGGTATCACTACTTTCTCGAGAAAGTAGTGATACCCAGTGGCA

SLITRK2 = SLIT And NTRK Like Family Member 2.
