# Supplementary material for: SLITRK2 knockdown rescues axonal and functional neuromuscular junction deficits in human iPSC-derived mutant FUS-ALS motor neurons
Source: Nat Commun. 2026 Oct 15;17:11662. doi: 10.1038/s41467-026-78419-7 (PMC13759529; doi:10.1038/s41467-026-78419-7)
Supplement: Supplementary file 3 [file 41467_2026_78419_MOESM2_ESM.pdf]

Description of Additional Supplementary Files

Supplementary Data 1: DEG and APA analysis comparing axonal and somatodendritic compartments of HC iPSC-derived motor neurons.
Differential expression analysis (DEG) was performed using DESeq2 (v1.42.0) applying a two-sided Wald test with Benjamini–Hochberg FDR correction. $p$adj<0.05 (DEG) or p<0.05 (APA), |Log2FC|>1.

Supplementary Data 2: DEG analysis comparing the axonal transcriptome of HC versus FUS-ALS, and FUS-iso versus FUS-ALS iPSC-derived motor neurons.
Differential expression analysis (DEG) was performed using DESeq2 (v1.42.0) applying a two-sided Wald test with Benjamini–Hochberg FDR correction. p<0.05, |Log2FC|>1.

Supplementary Data 3: GSEA results comparing the axonal transcriptome of HC versus FUS-ALS, and FUS-iso versus FUS-ALS iPSC-derived motor neurons.
Gene Set Enrichment Analysis (GSEA v4.3.2) performed using MSigDB C5 GO biological process gene sets. Listed are the ranked lists, positive and negative NES scores for each comparison. p<0.05.

Supplementary Data 4: APA analysis comparing 3’ UTR isoforms identified in the axonal transcriptome of HC versus FUS-ALS, and FUS-iso versus FUS-ALS iPSC-derived motor neurons.
Differential 3′ UTR isoform abundance was determined using DESeq2 (v1.42.0) applying a two-sided Wald test with Benjamini–Hochberg FDR correction; p<0.05, |Log2FC|>1.

Supplementary Data 5: Statistical report

Supplementary Video S1: Timelapse of contracting skeletal myotubes at D12 of the maturation protocol. Speed: 3 frames/s. Scale bar: 100 µm.
