# Supplementary material for: SLITRK2 knockdown rescues axonal and functional neuromuscular junction deficits in human iPSC-derived mutant FUS-ALS motor neurons
Source: Nat Commun. 2026 Oct 15;17:11662. doi: 10.1038/s41467-026-78419-7 (PMC13759529; doi:10.1038/s41467-026-78419-7)
Supplement: Supplementary file 4 [file 41467_2026_78419_MOESM9_ESM.pdf]

nature portfolio

Corresponding author(s): R.J. Pasterkamp

Last updated by author(s): Sep 4, 2026

Reporting Summary

Nature Portfolio wishes to improve the reproducibility of the work that we publish. This form provides structure for consistency and transparency in reporting. For further information on Nature Portfolio policies, see our Editorial Policies and the Editorial Policy Checklist.

Statistics

For all statistical analyses, confirm that the following items are present in the figure legend, table legend, main text, or Methods section.

n/a	Confirmed	
☐	☒	The exact sample size (*n*) for each experimental group/condition, given as a discrete number and unit of measurement
☐	☒	A statement on whether measurements were taken from distinct samples or whether the same sample was measured repeatedly
☐	☒	The statistical test(s) used AND whether they are one- or two-sided<br>*Only common tests should be described solely by name; describe more complex techniques in the Methods section.*
☐	☒	A description of all covariates tested
☐	☒	A description of any assumptions or corrections, such as tests of normality and adjustment for multiple comparisons
☐	☒	A full description of the statistical parameters including central tendency (e.g. means) or other basic estimates (e.g. regression coefficient) AND variation (e.g. standard deviation) or associated estimates of uncertainty (e.g. confidence intervals)
☐	☒	For null hypothesis testing, the test statistic (e.g. *F*, *t*, *r*) with confidence intervals, effect sizes, degrees of freedom and *P* value noted<br>*Give P values as exact values whenever suitable.*
☒	☐	For Bayesian analysis, information on the choice of priors and Markov chain Monte Carlo settings
☒	☐	For hierarchical and complex designs, identification of the appropriate level for tests and full reporting of outcomes
☒	☐	Estimates of effect sizes (e.g. Cohen's *d*, Pearson's *r*), indicating how they were calculated

Our web collection on statistics for biologists contains articles on many of the points above.

Software and code

Policy information about availability of computer code

Data collection	For qRT-PCR data: QuantStudio 6 Flex Real-Time PCR system (Applied Biosystems). For microscopy imaging: Zen 3.3 image acquisition software (Zeiss), Olympus Fluoview Version 4.1 imaging software, Nikon Ti2 Cicero or Nikon C2 laser scanning confocal microscope or Thunder Imaging System (Leica). For RNA-seq: Illumina Nextseq 2000 or NovaSeq X Plus Series (PE150) (somatodendritic versus axonal samples) or an Illumina Novaseq 6000 platform (FUS-ALS vs HC/FUS-iso axonal samples).
Data analysis	For qRT-PCR data: Excel (Microsoft). For microscopy data: Fiji ImageJ v1.53t software and Excel (Microsoft). For RNAScope: Fiji ImageJ v2.14.0/1.54f software and Excel (Microsoft). Axonal outgrowth analysis: Ilastik 1.4.0 Pixel Classification software, FiJi ImageJ v1.53t software and Excel (Microsoft). Calcium imaging: FiJi ImageJ v1.53t software and Excel (Microsoft). Plotting and statistics: GraphPad Prism 9.5.1 or 10 and R Studio v4.3.1. RNA-seq: R studio v4.3.1, DESeq2 package v1.42.0, Sierra R package, ClusterProfiler package v4.10.0 and GSEA software v4.3.2.

For manuscripts utilizing custom algorithms or software that are central to the research but not yet described in published literature, software must be made available to editors and reviewers. We strongly encourage code deposition in a community repository (e.g. GitHub). See the Nature Portfolio guidelines for submitting code & software for further information.

Data

Policy information about availability of data

All manuscripts must include a data availability statement. This statement should provide the following information, where applicable:

- Accession codes, unique identifiers, or web links for publicly available datasets
- A description of any restrictions on data availability
- For clinical datasets or third party data, please ensure that the statement adheres to our policy

RNA-seq and single cell RNA-seq data generated in this study have been deposited in the Gene Expression Omnibus (GEO) database under accession code GSE308345. Microscopy data reported in this paper or any additional information to reanalyze the data reported in this paper is available from the lead contact upon request.

Research involving human participants, their data, or biological material

Policy information about studies with human participants or human data. See also policy information about sex, gender (identity/presentation), and sexual orientation and race, ethnicity and racism.

Reporting on sex and gender	iPSC cell line: HC-1 (female). Source: Van der Geest et al., 2024<br>iPSC cell line: HC-2 (male). Source: Zelina et al., 2024<br>iPSC cell line: HC-3 (male). Source: Zelina et al., 2024<br>iPSC cell line: HC-4 (male). Source: Harschnitz et al., 2016<br>iPSC cell line: HC-5 (male). Source: Ormel et al., 2018<br>iPSC cell line: HC-6 (female). Source: Harschnitz et al., 2016<br>iPSC cell line: HC-7 (female). Source: Meyer et al., 2019<br>iPSC cell line: FUS-iso-1 (female). Source: Wenting Guo et al., 2017<br>iPSC cell line: FUS-iso-2 (male). Source: Wenting Guo et al., 2017<br>iPSC cell line: FUS-1 (female). Source: Wenting Guo et al., 2017<br>iPSC cell line: FUS-2 (male). Source: Wenting Guo et al., 2017<br>iPSC cell line: FUS-3 (female). Source: this paper<br>iPSC cell line: Knock-in WT (male). Source: Lenzi et al., 2015<br>iPSC cell line: FUS-P525L Knock-in (male). Source: Lenzi et al., 2015
Reporting on race, ethnicity, or other socially relevant groupings	We were not informed about variables such as race, etnicity or other socially relevant groupings because this data was anonymized.
Population characteristics	All obtained information about the population characteristics can be found in Supplementary Figure S2.
Recruitment	Recruitment was completed before the start of this study.
Ethics oversight	All experimental procedures involving iPSCs were approved by the Medical Ethical Committee of the University Medical Center Utrecht through biobank protocol 16–436. Donors gave written informed consent.

Note that full information on the approval of the study protocol must also be provided in the manuscript.

Field-specific reporting

Please select the one below that is the best fit for your research. If you are not sure, read the appropriate sections before making your selection.

☒ Life sciences ☐ Behavioural & social sciences ☐ Ecological, evolutionary & environmental sciences

For a reference copy of the document with all sections, see nature.com/documents/nr-reporting-summary-flat.pdf

Life sciences study design

All studies must disclose on these points even when the disclosure is negative.

Sample size	No statistical method was used to determine sample size. Sample size was determined by combining data from existing literature that used similar methodologies together with pilot experiments performed by the investigators. Sample size information is provided in figure legends and/or methods sections.
Data exclusions	For DESeq2-analysis of RNA-seq data, lowly expressed genes were excluded. For other assays, the ROUT method was used to determine and exclude outliers.
Replication	Multiple technical and biological replicates were used for each experiment (numbers are indicated in figure legends and methods section).
Randomization	Samples were randomly assigned to experimental groups (e.g. treated vs control) within each genotype.

Blinding

All NMJ and RNA quantification images were blinded for the genotype and experimental condition. The rest of the analysis were performed without blinding, given the lack of technical feasibility (investigator performing the experiments also did the analysis) and the type of analysis (automated analysis or analysis based on established equations, e.g. dCt method for qRT-PCR).

Reporting for specific materials, systems and methods

We require information from authors about some types of materials, experimental systems and methods used in many studies. Here, indicate whether each material, system or method listed is relevant to your study. If you are not sure if a list item applies to your research, read the appropriate section before selecting a response.

Materials & experimental systems

n/a	Involved in the study	
☐	☒	Antibodies
☐	☒	Eukaryotic cell lines
☒	☐	Palaeontology and archaeology
☐	☒	Animals and other organisms
☒	☐	Clinical data
☒	☐	Dual use research of concern
☒	☐	Plants

Methods

n/a	Involved in the study	
☒	☐	ChIP-seq
☒	☐	Flow cytometry
☒	☐	MRI-based neuroimaging

Antibodies

Antibodies used	All antibodies can be found in Supplementary Table S8.
Validation	All the antibodies used in this study were previously validated by the manufacturer using immunofluorescence staining or immunoblot. Validation information can be found at the corresponding vendor's website.

Eukaryotic cell lines

Policy information about cell lines and Sex and Gender in Research

Cell line source(s)	Human Embryonic Kidney 293 (HEK293T) (ATCC® CRL-3216™; RRID:CVCL_0063)<br>Human Neuroblastoma (SHSY5Y) (ATCC® CRL-2266™; RRID:CVCL_0019)
Authentication	The cell lines were not authenticated.
Mycoplasma contamination	Cells were not tested for mycoplasma contamination.
Commonly misidentified lines (See ICLAC register)	None

Animals and other research organisms

Policy information about studies involving animals; ARRIVE guidelines recommended for reporting animal research, and Sex and Gender in Research

Laboratory animals	Mouse strains: Non-transgenic C57Bl6J/Ntac. Mice were maintained in standard housing conditions, with a temperature of 22 ± 2 °C and humidity of 40-60%, on a diurnal 12-h light/12-h dark cycle and with continuous access to food and water. The mice used in this study were 12 months old at time of perfusion.
Wild animals	No wild animals were used in the study.
Reporting on sex	Data collected from male mice.
Field-collected samples	No field collected samples were used in the study.
Ethics oversight	All experimental procedures were conducted according to the KU Leuven ethical guidelines and approved by the KU Leuven Ethical Committee for Animal Experimentation (ECD number: Neuro-021/2025).

Note that full information on the approval of the study protocol must also be provided in the manuscript.

Plants

Seed stocks	*Report on the source of all seed stocks or other plant material used. If applicable, state the seed stock centre and catalogue number. If plant specimens were collected from the field, describe the collection location, date and sampling procedures.*
Novel plant genotypes	*Describe the methods by which all novel plant genotypes were produced. This includes those generated by transgenic approaches, gene editing, chemical/radiation-based mutagenesis and hybridization. For transgenic lines, describe the transformation method, the number of independent lines analyzed and the generation upon which experiments were performed. For gene-edited lines, describe the editor used, the endogenous sequence targeted for editing, the targeting guide RNA sequence (if applicable) and how the editor was applied.*
Authentication	*Describe any authentication procedures for each seed stock used or novel genotype generated. Describe any experiments used to assess the effect of a mutation and, where applicable, how potential secondary effects (e.g. second site T-DNA insertions, mosiacism, off-target gene editing) were examined.*
